# ICF-based hearing and functioning assessment: validation and research outcomes of utilizing the HEAR-COMMAND tool for patients with mild to moderately severe hearing loss and individuals with normal hearing

**DOI:** 10.3389/fresc.2024.1389653

**Published:** 2024-08-26

**Authors:** Tahereh Afghah, Razan Alfakir, Markus Meis, Mahmoud Hammady, Mostafa Youssif, Mohamed Abd Al-Ghaffar, Sophia E. Kramer, Kirsten C. Wagener

**Affiliations:** ^1^ Research Department, Hörzentrum Oldenburg gGmbH and Cluster of Excellence Hearing4all, Oldenburg, Germany; ^2^ ABILITY Research Lab, Department of Speech-Language and Hearing Sciences, Auburn University, Auburn, AL, United States; ^3^ Research Department, Cochlear Deutschland GmbH & Co. KG, Hannover, Germany; ^4^ Department of Otolaryngology, Head and Neck Surgery, Audiovestibular Medicine Division, Sohag University Hospital, Sohag, Egypt; ^5^ Department of Otolaryngology, Head and Neck Surgery, Amsterdam University Medical Center, Amsterdam, Netherlands

**Keywords:** International Classification of Functioning Disability and Health (ICF), hearing impairment, hearing loss questionnaire, self-reported hearing loss, communication disability, functioning

## Abstract

**Objective:**

Current clinical assessments for Hearing Loss (HL) are often limited to controlled laboratory settings in which a narrow spectrum of hearing difficulties can be assessed. A majority of the daily life challenges caused by HL cannot be measured in clinical methodologies. To screen the individuals' needs and limitations, a questionnaire named the HEAR-COMMAND tool was developed and qualitatively validated through an international collaboration, aligning with the World Health Organization's International Classification of Functioning, Disability, and Health Framework (ICF) Core Sets for Hearing Loss. The tool empowers healthcare professionals (HCPs) to integrate the ICF framework into patient assessments and patient-reported outcomes (PRO) in clinical and non-clinical settings. The aim is to provide a general foundation and starting point for future applications in various areas including ENT and hearing acoustics. The outcome can be employed to define and support rehabilitation in an evidence-based manner. This article presents the validation and research outcomes of using the tool for individuals with mild to moderately severe HL in contrast to normal-hearing individuals.

**Design:**

Using a cross-sectional multicenter study, the tool was distributed among 215 participants in Germany, the USA, and Egypt, filled in German, English, or Arabic. Three outcome scores and the corresponding disability degree were defined: hearing-related, non-hearing-related, and speech-perception scores. The content and construct validation were conducted, and the tool's internal consistency was assessed.

**Results:**

The extracted constructs included “Auditory processing functionality”, “Sound quality compatibility”, “Listening and communication functionality”, “Interpersonal interaction functionality and infrastructure accessibility”, “Social determinants and infrastructure compatibility”, “Other sensory integration functionality”, and “Cognitive functionality”. Regarding content validity, it was demonstrated that normal-hearing participants differed significantly from individuals with HL in the hearing-related and speech-perception scores. The reliability assessment showed a high internal consistency (Cronbach's alpha = 0.9).

**Conclusion:**

The outcome demonstrated the HEAR-COMMAND tool's high content and construct validity. The tool can effectively represent the patient's perspective of HL and hearing-related functioning and enhance the effectiveness of the treatment plans and rehabilitation. The broad range of targeted concepts provides a unique overview of daily life hearing difficulties and their impact on the patient's functioning and quality of life.

## 1 Introduction

Hearing loss (HL) adversely affects speech perception and communication abilities, giving rise to various psychosocial reactions, and is significantly influenced by personal and environmental factors. When assessing outcomes related to HL, a clear distinction is typically made between hearing (audiological) and non-hearing (non-audiological) considerations. Hearing aspects encompass both audiological test procedures, such as pure-tone audiometry and speech audiometry (in noise), as well as tools for self-reported states or outcomes regarding hearing difficulties and communication challenges. In contrast, non-hearing aspects encompass a wide range of parameters, including the patient's subjective quality of life, mental and psychological well-being, social interactions, relationships, and, in some cases, other sensory and motor functions (1–7).

The multifaceted aspects of HL align with the biopsychosocial model underpinning the World Health Organization's (WHO) International Classification of Functioning, Disability, and Health Framework (ICF) Core Sets for Hearing Loss (CSHL), including the Brief and Comprehensive versions (8–15). The “Brief ICF CSHL” and “Comprehensive ICF CSHL” serve distinct purposes in providing standardized descriptions of individuals' experiences with HL. The “Brief ICF CSHL” functions as a set of minimal requirements for reporting in audiological practices, offering a standardized depiction of individuals' HL experiences. On the other hand, the “Comprehensive ICF CSHL” includes a broader range of categories to offer a more detailed and thorough description of the functional problems associated with HL making it particularly useful in multidisciplinary assessments, providing a more nuanced understanding of the diverse impacts of HL on an individual's life.

The ICF is widely used in healthcare, rehabilitation, and disability studies to provide a standardized and universally applicable framework for assessing and addressing health-related issues (8, 16). The ICF comprises multiple domains to offer a comprehensive perspective on an individual's well-being and functioning. The ICF consists of two main components: (1) Functioning and Disability and (2) Contextual Factors. The functioning and disability component focuses on an individual's functioning, which includes their physical, mental, and social well-being. It encompasses various domains, including body functions (BF), body structures (BS), and activities and participation (AP). The contextual component focuses on personal factors (PF) and environmental factors (EF) essential for understanding the complete picture of an individual's health and functioning. An ICF category refers to a detailed concept listed within the hierarchical structure of the ICF domains which targets a specific aspect related to the addressed health condition. It is particularly valuable in understanding the multifaceted impact of conditions like HL and in tailoring interventions to meet the individual needs of patients. By considering all these domains and factors, healthcare professionals (HCPs) can provide more comprehensive and person-centered care for individuals with hearing-related conditions.

In clinical practice, systematically evaluating both hearing and non-hearing aspects of HL can be complex. The report “Hearing Health Care for Adults: Priorities for Improving Access and Affordability” (2016) by the National Academies of Sciences, Engineering, and Medicine emphasizes a significant issue in audiological test procedures (17). It underscores a frequent weak correlation or mismatch between the measured HL and the reported difficulties. When the reported challenges do not align with the severity of clinically measured HL, it becomes crucial to investigate non-hearing aspects, such as psychosocial factors, and their determinants, including the unique environments in which individuals are functioning. This recognition highlights the importance of a comprehensive approach to understanding and addressing hearing health beyond the purely audiological test procedures. Numerous studies have demonstrated that evaluating both hearing and non-hearing aspects, as outlined in the ICF CSHL, enables the development of a foundational set of questions. These questions can be established and standardized for diverse populations across different countries and, subsequently, can serve as a conduit for transcending borders. This is particularly crucial for delivering comprehensive and patient-centered aftercare (5, 6, 17–26). The initial step in the rehabilitation process is documentation. Two studies (6, 25) investigated the overlap and non-overlap between the current Otology and Audiology intake documentation in multiple centers in the Netherlands and the Mayo Clinic in the USA. Both studies revealed that while many ICF categories were considered in various measures, each one missed some essential categories.

The HEAR-COMMAND Tool^1^ is a self-assessment questionnaire that draws its foundation from the ICF CSHL framework (27). The tool was developed with a broad targeted potential usage, having in mind all degrees of hearing loss, primarily to enhance the patient-centeredness of audiological services, treatment, and rehabilitation for adult patients dealing with HL as a basis for future applications and research. Conceptually, it specifically evaluates the challenges individuals face in hearing, daily functioning, communicating, and engaging in conversations. The tool was designed to address these gaps by covering all the essential aspects necessary for comprehensive ICF-based documentation. By ensuring that all relevant ICF categories are included, the HEAR-COMMAND Tool provides a more complete and standardized approach to documenting patient information, thereby enhancing the quality and consistency of patient management in the rehabilitation process. This tool is especially valuable for tracking patient progress and outcomes in both aural and non-aural rehabilitation, making it a critical component for clinicians working with individuals with HL.

The tool development procedure was a collaborative effort involving a team from multiple countries, including Germany, the USA, the Netherlands, and Egypt. This collaboration resulted in the creation of versions in English, German, and Arabic. The tool's availability in three languages makes it applicable in various linguistic and cultural contexts and ensures that it can be utilized effectively in diverse patient populations, further advancing its potential to improve the quality of care for individuals with HL universally. The tool development encompassed 5 steps; 1. ICF categories selection. 2. Item creation and terminology adjustment for the selected categories. 3. Specific item formulation, scoring method, and experts' feedback. 4. The development of the questionnaire's beta (initial) version; experts' perspectives. The authors of this paper were all actively involved in the development process and evaluated the tool content from a broad perspective drawing on their diverse backgrounds. 5. The development of the questionnaire's revised version; patients' perspectives. In this step, the qualitative analysis of the initial version of the tool was performed. The responses of 109 participants including 25 individuals with normal hearing and 84 individuals with different HL degrees, either unaided or hearing aid users, in Germany, the USA, and Egypt were collected. As a result, two new items were added to the initial version of the tool, and the terminology of 27 items was modified. For the details of each step and the terminology modifications applied to the initial version, please refer to (27, section 2.3). Accordingly, a revised version of the tool was created and used in this study.

The questionnaire consists of 90 items designed to collect information on functioning in the ICF BF, AP, and EF domains. Responses are rated on a scale from 0 to 4, with the following terminology assigned to each number: 0 (No), 1 (Mild), 2 (Moderate), 3 (Severe), and 4 (Profound/Complete). Given that PFs are not specifically coded in the ICF because of the wide variability among cultures, the tool encompasses an additional 30 thirty items designed to collect a range of individual characteristics related to hearing health and functioning, such as age, gender, education level, employment status, lifestyle, social background, and personal experiences. By including these items, the questionnaire aims to provide a more holistic understanding of the individual's context and how these factors may interact with their functioning and health outcomes. Except for the BS domain, the brief ICF CSHL categories were covered, encompassing a total of 85% of these categories. Furthermore, the items addressed 44% of the comprehensive ICF CSHL categories, including 73% of BF, 55% of AP, and 27% of EF domains.

Patient-reported outcomes (PROs) based on the ICF CSHL have gained importance and attention worldwide. Table 1 summarizes the information regarding the questionnaires developed based on the ICF CSHL. In contrast to the HEAR-COMMAND tool that covers a significant portion of the comprehensive ICF CSHL categories (44%), the questionnaires mentioned in Table 1 were created based on the brief ICF CSHL. As the HEAR-COMMAND tool was grounded upon 52 categories, it offers a holistic approach to integrating hearing health-related factors for individuals with HL. With a substantial coverage of 73% of the categories in the BF domain of the comprehensive ICF CSHL, the HEAR-COMMAND tool delves into various non-audiological aspects relevant to hearing health. This detailed exploration allows for a nuanced understanding of the intricate interplay between the HEAR-COMMAND scales and subscales. Besides hearing and functioning, this tool focuses on the challenges brought on by HL on the daily communication and conversation tasks which the corresponding ICF categories are excluded from the brief ICF CSHL.

**Table 1:** An overview of the questionnaires developed based on the ICF core sets for hearing loss.

#	Publication year	Questionnaire title	Number of items	Overall number of participants -	Countries	Languages	ICF core sets	Intended applications
1	2017	Self-assessments ICF core sets for hearing loss questionnaire	17	131	United States	English	Brief (excluding BS)	"Audiological services, treatment, and rehabilitation efficiency enhancement” (20).
2	2020	ICF-based e-intake tool	62	11	Netherlands	Dutch	Brief (including BS)	"To be used as an intake assessment in otology and audiology practice” (21).
3	2022	HEAR-COMMAND tool	120	324 [109 in qualitative analysis represented in (27) and an additional 215 included in this study]	Germany, United States, Netherlands, Egypt	English German Arabic	Brief (excluding BS) And Comprehensive (44% of the categories)	To evaluate hearing, functioning, communication and conversation disability and to be used for rehabilitation, treatment, and audiological services (27).
4	2023	Hearing and functioning in everyday life questionnaire (HFEQ)	30	30	India, South Africa, United States	English	Brief (excluding BS)	"To evaluate everyday functioning of patients with hearing loss in rehabilitation” (24).

BS, body structures.

The “ICF-Based e-Intake Tool”, developed in the Netherlands in 2020, consists of 62 items linked to 39 ICF categories (21). Apart from personal factors and the ICF categories included in the Brief and Comprehensive CSHL, “ICF-Based e-Intake Tool” and the HEAR-COMMAND tool contain the following additional ICF categories which are not included in the CSHL; “Sleep functions” (b134), “Taste function” (b250), and “Smell function” (b255) (21, 27). With regards to “Taste functions” and “Smell functions”, multiple sensory losses are common in individuals with HL. On one hand, HL is frequently described as a consequence or side effect of defined entities such as ontological, cardiovascular, infectious, and neurological diseases. On the other hand, eating and drinking are common behaviors during family or social gatherings, which may influence how a person with HL functions when engaged in communication and conversation activities.

Furthermore, the Quality of Life in People with Hearing Loss Questionnaire (HLQoL) (28) was developed in Germany and consists of 21 items. It was validated in a group of cochlear implant users. This questionnaire contains ICF categories not included in the ICF CSHL, such as “Making decisions” (d177), “Carrying out daily routine” (d230), and “Moving around in different locations” (d460).

The primary objective of this study is to validate the HEAR-COMMAND Tool and assess its reliability across multiple centers. The validation and reliability assessments spanned both individuals with normal hearing and those with mild to moderately severe HL. To ensure broad applicability, the research incorporates versions of the tool in English, German, and Arabic, catering to the linguistic diversity of the study participants. The validity assessment was conducted by performing content and construct validity and the reliability was evaluated by measuring Cronbach's alpha (29, 30). The results section includes the following information. (1) An analysis of the participants' responses to the ICF-based items of the tool. (2) The results of the tool's content and construct validity evaluation. (3) The outcome of the reliability assessment by measuring Cronbach's alpha.

## 2 Material and method

### 2.1 HEAR-COMMAND tool questionnaire

The English version of the questionnaire can be found in Supplementary Material S1. The initial set of 30 demographic items was developed following a literature review on worldwide available HL questionnaires, functioning impairment assessment tools, general health inquiry surveys, and various questionnaires developed by the WHO in this area. The items identified in these sources were selected by experts and modified or adapted based on their opinions. For detailed information regarding these sources, please refer to the tool's development paper (27), page 4, Table 1. Furthermore, certain items were newly developed based on the expert's recommendation. Table 2 summarizes the concepts targeted in each ICF-based item for convenient access.

**Table 2:** The summary of the targeted concept of ICF-based items of the HEAR-COMMAND tool.

Body functions		Body functions		Activities and participation		Environmental factors	
#	Item content	#	Item content	#	Item content	#	Item content
H.1	Mood swings	H.25	Detecting noise in household	H.49	Dealing with stressful situations	H.75	Support received from society
H.2	Sleeping	H.26	Discriminating the sound of a car/bus	H.50	Interacting with people in a socially	H.76	Emotional support from family/friends
H.3	Focusing attention	H.27	Recognizing musical instruments	H.51	Socializing with people in your community	H.77	Support from family/friends in daily functioning
H.4	Maintaining focus	H.28	Detecting where a sound comes from	H.52	Dealing with unknown people	H.78	Support from health services/systems
H.5	Remember information	H.29	Telling a bus/truck is getting close or far	H.53	Having formal relationships with people in authority	H.79	Support from healthcare professional
H.6	Recall new information	H.30	Detecting corner of a room when one is talking	H.54	Socializing with your family or friends	H.80	Communication services/systems usefulness
H.7	Sadness or depression	H.31	Telling how far away a bus/truck is	H.55	Making new friends	H.81	Design of workplace as a barrier
H.8	Seeing across the road	H.32	Telling where a human is when he screams/dog barks	H.56	Having an argument or debate	H.82	Insufficient light as a barrier
H.9	Seeing over an arm length	H.33	Detecting whether the person on left/right starts talking	H.57	Understanding a statement during communication	H.83	Low volume of speech as a barrier
H.10	Taste loss	H.34	Hearing a single jumbled sound when hearing more than one sound	H.58	Maintaining relationships with immediate family	H.84	Background noise as a barrier
H.11	Smell loss	H.35	Understanding the speech over distance	H.59	Joining in community activities	H.85	Reverberant environment as a barrier
H.12	Dizziness	H.36	Understanding the speech in a quiet environment	H.60	Engaging in any hobby or pleasurable activity	H.86	Unclear sound considered a barrier
H.13	Loss of balance	H.37	Understanding the speech in a noisy environment	H.61	Continuing relationships in an appropriate manner	H.87	Hearing aid usefulness in normal daily routines
H.14	Pain (general)	H.38	Understanding news presenter on TV	H.62	Performing communication techniques	H.88	Hearing aid usefulness in conversation activities
H.15	Pain (head & neck)	H.39	Understanding what one is saying while the TV is on	H.63	Your day-to-day tasks	H.89	Hearing aid usefulness while using phone
H.16	Understanding meaning of a message	H.40	Understanding the news presenter and someone else	H.64	Doing your most important tasks well	H.90	Hearing aid usefulness while watching TV
H.17	Producing a meaningful message	H.41	Having health conditions causing speech impairment	H.65	Getting done all the tasks		
H.18	Ringing/buzzing in ears	H.42	Making sounds other than speech	H.66	Getting your tasks done quickly		
H.19	Pressure in ear	H.43	Changing pitch of sounds other than speech	H.67	Conversation or speaking with someone		
H.20	Irritation in ear	H.44	Changing volume of sounds other than speech	H.68	Conversation or speaking with many people		
H.21	Distinguishing pitch	H.45	Pronunciation	H.69	Carrying on a conversation during a crowded meeting		
H.22	Distinguishing tone	H.46	Regulating the volume of speech	H.70	Carrying on a conversation in a bus or car		
H.23	Distinguishing volume	H.47	Regulating the speed of speech	H.71	Following a conversation in a busy restaurant		
H.24	Detecting a sound in environment	H.48	Telling stories or reporting	H.72	Carrying a phone call in a quiet room		
				H.73	Telling what one is saying when conversation switches		
				H.74	Listening to the TV/Radio/Music		

### 2.2 Participants' recruitment and data collection

The data were collected from 215 participants in three countries. The participants were provided with the tool in their native language (German in Germany, Arabic in Egypt, and English in the United States) from Oct 2022 to Aug 2023 in pencil-paper format. In all countries, based on the measured pure-tone average (PTA) recorded in the database of volunteers or former patients, the participants who were classified as individuals with mild to moderately severe HL, whether they were hearing aid users or not, were recruited. Additionally, overall, a total of 58 participants with no reported HL were recruited. Recruitment was performed with and without consideration of the participants' knowledge of the cause of their HL or the previous clinical diagnostics. On average, it takes approximately 15–20 min to complete the questionnaire.

Data collection in Germany was performed in Hörzentrum Oldenburg gGmbH, an institute at the University of Oldenburg. The participants were selected from the research volunteer's database, which includes records of the most recent pure tone audiometry measurements performed on the research volunteers. The printed questionnaire and the consent form were given either in person or by postal service to the participants. The filled questionnaire was either brought back in the follow-up visit or sent back by mail. Upon arrival at the institute's clinic, an audiologist performed the pure tone audiometry measurement.

Data collection in the United States was conducted within the Ability Lab at Auburn University's Department of Speech-Language and Hearing Sciences, in partnership with the Hearing and Speech Clinic. Data collection in Egypt was performed in the Department of Otolaryngology, Head and Neck Surgery, Audiovestibular Medicine Division, Sohag University Hospital, Sohag, Egypt. In the United States and Egypt, the consent form and the tool were filled in by the participant during the visit to the clinic after the standard of care audiologic procedures.

### 2.3 Respondents' characteristics

#### 2.3.1 General demographics

Table 3 demonstrates the summary of the participant's demographic information. For detailed responses, please refer to Supplementary Material S2, Figures A.1–A.14. Furthermore, a summary of the results of performing pure-tone audiometry is given in this table and the details are shown in Supplementary Material S2, Figures P.1–P.5.

**Table 3:** The characteristics of the participants in each country.

Characteristics		Overall	Germany	Egypt	USA
Number of participants	*N*	215	89	54	72
Gender	Male, *N* (%)	121 (56)	46 (51)	24 (44)	51 (71)
	Female, *N* (%)	94 (44)	43 (49)	30 (56)	21 (29)
Age (years)	Range	20–84	31–84	20–70	22–80
	M ± SD	58.4 ± 18.8	68.5 ± 12.7	40.6 ± 16.3	59.2 ± 17.3
Educational level (years)	M ± SD	15.4 ± 3.3	14.4 ± 3.8	15.5 ± 3.3	16.2 ± 3.1
Normal hearing	*N* (%)	58 (27)	30 (34)	10 (19)	18 (25)
Hearing impaired, unaided	*N* (%)	87 (41)	29 (33)	33 (61)	25 (35)
Hearing impaired, aided (hearing aid users)	*N* (%)	70 (32)	30 (33)	11 (20)	29 (40)
Pure-Tone Average (dB HL)	Range	−2 to 64	−1.2 to 60	10–55	−2 to 64
	M ± SD	31. 0 ± 16.1	29.7 ± 16.9	33.8 ± 14.4	30.5 ± 16.0

Mean and standard deviations are given by M and SD, respectively. PTA was calculated for the better ear across 0.5, 1, 2, 4 KHz.

Fifty percent of the participants were over 65 years old. Eighty percent of them had postsecondary education (more than 12 years of education). Only one participant had attended a special school for children with hearing impairment. Sixty-six percent of them were currently married or cohabiting and living with a partner (with or without children). Forty-eight percent of the participants were retired.

The HL classification was conducted according to the guidelines and recommendations of the “Global Burden of Disease Expert Group on Hearing Loss Classification” (31, page 38; 32) and the PTA is calculated across 0.5, 1, 2, and 4 kHz. The participants were classified into three major groups. (1) “Normal hearing individuals” refers to those with a PTA value below 20 dB HL. (2) “Individuals with HL, unaided” refers to those with a PTA above 20 dB HL who currently do not use any hearing aids. (3) “Individuals with HL aided” refers to those with a PTA above 20 dB HL who currently use hearing aids. This group was asked to respond to the items considering their condition while using their hearing aid(s). Accordingly, 73% of the included participants were categorized as individuals with mild to moderately severe HL (either aided or unaided). Among the unaided group, 54% of them had mild HL (PTA in the range of 20–35 dB HL), 39% had moderate HL (PTA in the range of 35–50 dB HL), and 7% had moderately severe HL (PTA in the range of 50–65 dB HL). Among the aided group, 13% had mild HL, 53% had moderate HL, and 34% had moderately severe HL. For the exact participants' degree of HL in each country, refer to Supplementary Material S2, Figure P.5. The average PTA difference between better and worse ears was 6.1 dB HL (SD = 5.9). Among all the participants, the highest PTA of the worst ear was 85 dB HL. Nine participants had better and worse ear PTA differences equal to or larger than 15 dB HL.^2^

#### 2.3.2 Co-morbid health conditions

Overall, 53% currently have a diagnosed condition, and 9% have an undiagnosed condition. In our diverse cohort, the prevalence of diagnosed health conditions among participants varied, with Hypertension ranking highest at 31% overall. Broken down by country, the percentages were 37% in Germany, 13% in Egypt, and 38% in the USA. Diabetes Mellitus (DM) was the second most reported condition, with 15% of participants in Egypt, 4% in Germany, and 19% in the USA indicating a diagnosis. Pain and tension in the neck area were reported by 15% of the overall participants (based on the responses to item H.15).

#### 2.3.3 General ear and hearing inquiries

In the following, a summary of the responses regarding the current and past ear and hearing health conditions is provided. Detailed responses can be found in Supplementary Material S2, Figures A.15–A.26. Surgical treatment of the ear was performed on twelve participants in the past and is planned on three participants. Middle ear infections and running ears were reported by 26% and 6% of participants, respectively. The hearing of 62% of participants was evaluated less than a year ago. Sudden HL was experienced by 16% of the participants. The participants were asked to indicate if they knew the cause of their HL where 48% responded yes, 31% responded no, and 20% responded not applicable. Age-related HL was the most self-reported main cause of HL, followed by Noise-induced HL, and HL caused by exposure to a bang, explosion, or shot. Evaluating the potential impacts of HL etiologies on responses to the ICF-based items is beyond the scope of this paper. However, future studies could investigate the correlation between the HEAR-COMMAND Tool outcome and the causes of HL. For instance, the responses of patients with sudden HL can be compared to those exposed to loud noise for a long time or those diagnosed with age-related HL (Presbyacusis). Having a family history of HL was reported by 43% of the participants.

Detailed responses to the last four items which only target hearing aid users are provided in Supplementary Material S2, Figures A.27–A.30. Eighty-eight percent of the users wear hearing aids in both ears. On average, the users started using hearing aids 9 years ago for the first time (SD = 8 years). Sixty-three percent of the users wear the aid for more than 8 h a day.

### 2.4 Statistical data analysis

Data analysis was performed using the IBM “Statistical Package for the Social Sciences” (SPSS) v.25 (33) and MATLAB R2021b. The content validity was performed via the following calculation: The quantitative analysis, descriptive statistics, and the floor and ceiling effects (34) with a 15% cut-off were measured for individual items. The Mann–Whitney U test (35) was performed at the significance level of *p *= 0.05 to find significant differences. In the construct validity assessment, Principal axis factoring was estimated for the Exploratory Factor Analysis (EFA) with Promax rotation (36, 37). The eigenvalues above unity were considered. The suitability of performing EFA was evaluated based on the Kaiser-Meyer-Olkin (KMO) Measure of Sampling Adequacy (38, 39) and Bartlett's test of sphericity (40). The reliability of the tool is demonstrated by measuring the internal consistency using the Cronbach Coefficient alpha statistic (29, 30).

## 3 Results

### 3.1 Content validity

#### 3.1.1 Quantitative analysis

For an overview of the distribution of response options for each ICF-based item, refer to Supplementary Material S2, Table 1. The minimum response [0 in the Likert scale (41)] was chosen for all the items except for two items inquiring about the usefulness of hearing aids in normal daily routines (H.87) and during listening-conversation activities (H.88). The maximum response (4 in the Likert scale) was chosen in 89% of the items. Apart from the numeric responses, two other response options were provided for each item; “I don't know” and “Not applicable”. If a participant was not a hearing aid user, the items H.87 to H.90 were recorded as “Not required” in the database. Similarly, if the response of a participant to H.41 (inquiring about speech impairment) was “no”, the items H.42 to H.48 were recorded as “Not required”. For each item, the “non-gradable” rate was calculated which includes the percentage of the responses which fall into any of these categories: “I don't know”, “Not applicable”, “Not required”, or “Missing response”.

For items H.87 to H.90, the rate is 67.4% as it was required only for hearing aid users, and for H.42 to H.48, it is 95% as it was only required for participants with speech impairment. Seven additional items had a non-gradable rate ranging from 14.9% to 29.8% (H.64, H.27, H.63, H.82, H.78, H.62, and H.81). Primarily, this high rate was the result of choosing “not applicable” response for the items were addressing concepts such as functionality in the workplace that do not apply across all participants. For a detailed explanation of the non-gradable rate of these items, refer to Supplementary Material S2, Table 2. Excluding the aforementioned items, the average non-gradable rate over the remaining items was 4.2% (SD = 3.5).

#### 3.1.2 Descriptive statistics

Detailed descriptive statistics are provided in Supplementary Material S2, Table 1 including the minimum, maximum, mean, standard deviation, and median values calculated from the responses corresponding to each item. In the following, specific information is provided for some of the items. The reason for including these items is to demonstrate how the participants' responses align with established knowledge about HL and its impact on daily life. This alignment serves as conceptual proof of the tool's validity.

Although the median value is recommended for the statistics of ordinal data, since the median of many items is mostly 0 or 1, here the corresponding mean value is used to sort the items. In general, the highest mean values, ranging between 2.3 and 3.4 on a scale of 0 to 4, were observed in EF, facilitators (H.75 to H.80 representing the overall usefulness of the systems/services or support received from people), and H.87 to H.90 (usefulness of hearing aids). This was expected as in the case of EF, facilitators, the number 4 is assigned to the highest level of support. Among these items, H.87 (usefulness of hearing aids in normal daily routines) and H.88 (during listening-conversation activities) had the highest mean values (3.4). These are followed by H.80 (usefulness of the communication services and systems used daily) and H.90 (usefulness of hearing aids while watching TV) with mean values of 3.1 and 3, respectively. The second highest set of mean values corresponds to EF, barriers (H.83 to H.86) ranging from 1.9 to 2.

Among the items corresponding to BF and AP domains, H.71 (following a conversation in a restaurant) had the highest, and H.10 (problem with taste loss) had the lowest mean values (1.7 and 0.2, respectively). Furthermore, H.37 (understanding speech in a noisy environment), H.40 (understanding the news presenter and someone else), H.39 (understanding what someone is saying while the TV is on), H.34 (hearing a single jumbled sound when hearing more than one sound), and H.69 (carrying on a conversation during a crowded meeting) had the 3rd^,^ 5th, 6th, 7th, and 9th highest mean values. These items describe an auditory scene in which speech or sound in general must be detected in the presence of competing sounds (auditory masking).

Item H.18, having a problem with ringing, beeping, roaring, or buzzing in ears had the second highest mean value of 1.6. Detecting a sound (H.24), and distinguishing the volume (H.23), pitch (H.21), and tone (H.22) of it were ranked as 13th, 15th, 16th, and 19th items with a high mean value. Item H.2 (problem with sleeping, ICF category: b134) had the 4th highest mean value (1.4). The 8th and 12th ranked means (1.3 and 1.1 respectively) correspond to memory functions, remembering things and recalling new information (H.5 and H.6). The 10th highest mean (1.2) belongs to H.4 (maintaining focus on two or more things at the same time).

Floor and ceiling effects were measured for all the items excluding speech-impairment items. If more than 15% of the participants chose the highest possible response (4 on the Likert scale), the ceiling effect existed and similarly when more than 15% of them chose the lowest value (0), the floor effect existed. For all BF and AP items, the floor effect was present, and the ceiling effect was absent. For EF items, the floor effect was present only for items H.81 and H.82. Note that these two items have a non-gradable rate of 29% and 21%, respectively. The ceiling effect was present for all the facilitator items and absent for all the barrier items.

In the subset of aided hearing-impaired participants, the floor effect is absent for H.34 (hearing a single jumbled sound when you are hearing more than one sound at a time), H.37 (understanding the speech of someone you know in a noisy environment), H.39 (understanding what someone is saying while the TV is on at the same time), H.40 (understanding the presenter of the news on the radio or TV and understanding what someone is saying at the same time), and H.71 (following a conversation between five people in a busy restaurant) which all belong to BF and AP domains.

### 3.2 Construct validity

#### 3.2.1 Exploratory factor analysis

EFA was conducted on the outcome of utilizing the tool for the sample population that is the responses provided by normal-hearing individuals and those with mild to moderately severe HL. This was to reveal the ground factors underlying the fundamental relationship between the items based on the patient's perspective.

In this analysis, two groups of items were excluded; Items H.41 to H.48 related to “voice and speech production functions”. Item H.41 is the “filter question” developed for this group; “*H.41: Do you have any health conditions causing speech impairment or producing sounds?*”. Overall, 11 participants (5%) claimed they had speech production impairment of which one had normal hearing, seven were unaided and three were aided hearing-impaired. Six of them were diagnosed with a brain disorder or had a stroke. The other group of excluded items are H.87 to H.90 which correspond to “hearing aid benefits” and which were required solely for the participants who use hearing aids.

The remaining 78 items were responded to by the entire sample population. The calculated KMO value was 0.920 and Bartlett's test of Sphericity resulted in a chi-square of *X^2^* (df:3003) = 14,499.8, *p*-value ≤ 0.001. Table 4 shows the rotated factor matrix by applying Promax rotation. The resulting factors explained 66.7% of the variance.

**Table 4:** The results of performing exploratory factor analysis.

Pattern matrix^a^														
	Factor													
	1	2	3	4	5	6	7	8	9	10	11	12	13	
H.25	0.914													A
H.24	0.905													
H.33	0.824													
H.30	0.811													
H.23	0.806													
H.22	0.770													
H.21	0.755													
H.29	0.749													
H.28	0.666													
H.32	0.655													
H.27	0.618													
H.35	0.587												0.341	
H.31	0.574													
H.38	0.526												0.425	
H.19	0.504													
H.18	0.500													
H.34	0.496				0.405									
H.26	0.416											0.338		
H.39	0.394				0.360								0.309	
H.73	0.381													
H.55		0.892												B
H.64		0.842												
H.65		0.839												
H.51		0.753												
H.60		0.750												
H.52		0.727												
H.53		0.725												
H.59		0.666												
H.56		0.650												
H.50		0.632												
H.63		0.620												
H.66		0.620												
H.58		0.613												
H.54		0.596												
H.61		0.561												
H.7		0.547												
H.49		0.525					0.370							
H.81		0.423												
H.83			0.792											C
H.86			0.790											
H.84			0.741											
H.85			0.725											
H.76				0.833										D
H.77				0.743										
H.78				0.728										
H.79				0.713										
H.75				0.702										
H.80				0.402										
H.71					0.985									E
H.69					0.891									
H.70					0.760									
H.68		0.417			0.637									
H.37					0.570									
H.40					0.486									
H.57					0.209									
H.9						0.972								F
H.8						0.859								
H.20						0.317								
H.1							0.810							G
H.4							0.717							
H.6							0.516							
H.5							0.515							
H.2							0.485							
H.3							0.464							
H.13								0.783						F
H.12								0.767						
H.11									0.850					
H.10									0.840					
H.15										0.655				
H.14										0.610				
H.17											0.836			G
H.16											0.750			
H.62												0.483		E
H.82												0.408		
H.72												0.407		
H.67					0.303							0.310		
H.36	0.452												0.649	
H.74													0.369	

A: Auditory Processing Functionality, B: Interpersonal Interaction Functionality and Infrastructure accessibility, C: Sound quality compatibility, D: Social Determinants and Infrastructure compatibility, E: Listening and Communication Functionality, F: Other Sensory Integration Functionality, G: Cognitive Functionality.

^a^ Rotation converged in 15 iterations.

**Figure 1:**
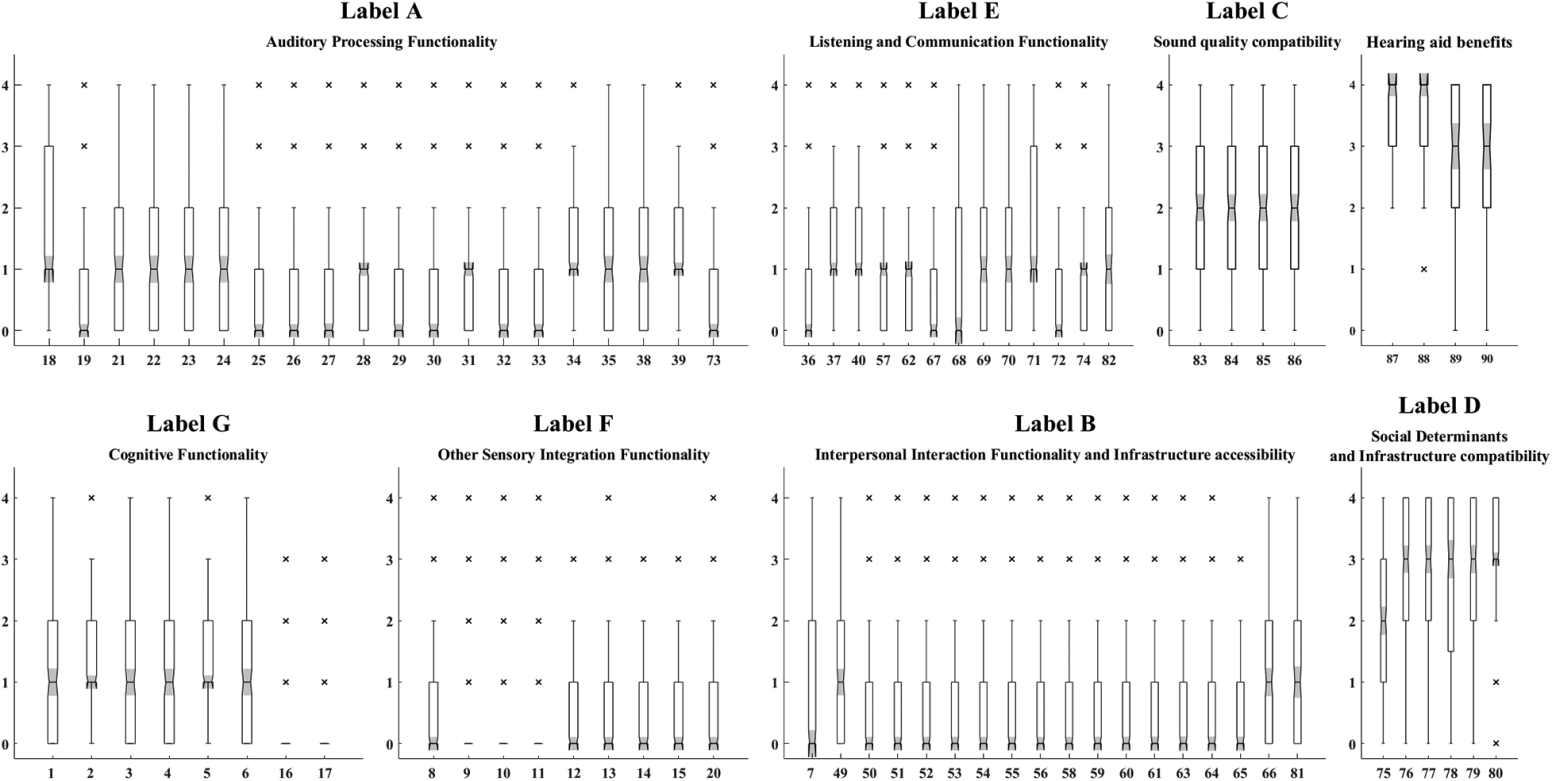
The distribution of responses to the items of each label over all the participants. The numbers on the x-axis show the item numbers. The y-axis shows the responses on the Likert scale. In all items 0, 1, and 2 correspond to “no”, “mild”, and “moderate” degrees respectively. The value 3 corresponds to “severe” in all items except for the facilitators of the environmental factors domain (H.75 to H.80 and H.87 to H.90) which correspond to “substantial”. The value 4 corresponds to “profound or complete” in items H.1 to H.74 and “complete” in items H.75 to H.90. The responses to “voice and speech production functions” (H.41 to H.48) are excluded as the responses are limited to 11participants.

In Table 4, the labels A to G correspond to the names assigned to the factor(s) including items with similar underlying concepts based on experts' opinions. Factor 1 contains the items that are related to the overall capability of detecting sound in the environment, proficiency in sound feature recognition, and the physiological requirements needed for accurate sound perception. This factor is named “A: Auditory Processing Functionality”. Factor 2 encompasses the items referring to the ability to engage in society at various levels and the capability of establishing relationships with familiar and unfamiliar individuals as well as the proficiency of applying the provided services and systems, named “B: Interpersonal Interaction Functionality and Infrastructure accessibility”. It was observed that the items loaded on factor 3 correspond to EF domain, barriers. These items describe how the acoustical features of the surrounding area, and the quality of produced sound can impact the perception of sound. This factor is named “C: Sound quality compatibility”. On the other hand, the items loaded on factor 4 correspond to EF domain, facilitators. These items ask about the influence of received support from social networks and HCPs on overall functioning and the feasibility of deploying the provided services to establish and enhance communication. This factor is named “D: Social Determinants and Infrastructure compatibility”. Items loaded on factor 5 specifically address the ability to conduct a conversation in a noisy environment, in the presence of auditory maskers. Factors 12 and 13 encompass conducting communication in less challenging settings, such as one-on-one conversation or in quiet environments, along with the requirements and communication capabilities. Due to the similarity in the contents targeted in factors 5,12, and 13, they are merged and named “E: Listening and Communication Functionality”. Factor 6 (seeing ability), factor 8 (dizziness and loss of balance), factor 9 (taste and smell loss), and factor 10 (pain), all refer to symptoms or sensing the external triggers, not directly related to hearing. Therefore, they are merged and named “F: Other Sensory Integration Functionality”. Factor 7, referring to mental and psychological functioning, such as attention and memory functions, is merged with factor 11 addressing mental functions of language, and named “G: Cognitive Functionality”. Figure 1 demonstrates the distribution of responses to the items of each label over all the participants.

Table 5 represents the corresponding ICF categories linked to the items included in each label. The extracted factors can include a mixture of ICF categories originating from distinct ICF domains. For instance, categories b1560 (auditory perception), b230 (hearing functions), and d115 (listening) are derived from different ICF domains (BF and AP), yet grouped in the same factor (factor 1/label A). It can be concluded that communication and conversation disability are not solely the results of problems with the body's hearing functioning (included in the BF domain) or difficulty with listening to a sound (included in the AP domain), but rather an interplay of multiple factors. This finding is consistent with the ICF's holistic approach to evaluating health conditions.

**Table 5:** The ICF categories linked to the HEAR-COMMAND tool items, classified based on the exploratory factor analysis extracted factors.

Hearing-related items			Non-hearing-related items			
A	C	E	B	D	F	G
Auditory Processing Functionality	Sound quality compatibility	Listening and Communication Functionality	Interpersonal Interaction Functionality and Infrastructure accessibility	Social Determinants and Infrastructure compatibility	Other Sensory Integration Functionality	Cognitive Functionality
Factor 1	Factor 3	Factors 5, 12, 13	Factor 2	Factor 4	Factors 6, 8, 9,10	Factors 7, 11
b1560 Auditory perception (H.21/H.22/H.23 /H.35) b2300 Sound detection (H.24/H.25) b2301 Sound discrimination (H.26/H.27/H.34) b2302 Localization of sound source (H.28/H.29/H.30/H.31/H.32) b2303 Lateralization of sound (H.33) b2304 Speech discrimination (H.38 /H.39) b240 Sensations associated with hearing and vestibular functions (H.18/H.19) d3503 Conversing with one person (H.73)	e2500 Sound intensity (H.83) e2501 Sound quality (H.84/H.85/H.86)	b1560 Auditory perception (H.36/H.37) b2304 Speech discrimination (H.40) d115 Listening (H.74) d310 Communicating with-receiving-spoken messages (H.57) d3500 Starting a conversation (H.67/H.68) d3501 Sustaining a conversation (H.67/H.68) d3502 Ending a conversation (H.67/H.68) d3503 Conversing with one person (H.67/H.69/H.70) d3504 Conversing with many people (H.68/H.71) d360 Using communication devices and techniques (H.62/H.72) e240 Light (H.82)	b152 Emotional functions (H.7) d220 Undertaking multiple tasks (H.63/H.64/H.65/H.66) d240 Handling stress and other psychological demands (H.49) d355 Discussion (H.56) d710 Basic interpersonal interactions (H.50) d720 Complex interpersonal interactions (H.61) d730 Relating with strangers (H.52) d740 Formal relationships (H.53) d750 Informal social relationships (H.51/H.54/H.55) d760 Family relationships (H.58) d820 School Education (H.63/H.64/H.65/H.66) d830 Higher education (H.63/H.64/H.65/H.66) d850 Remunerative employment (H.63/H.64/H.65/H.66) d855 Non-remunerative employment (H.63/H.64/H.65/H.66) d910 Community life (H.59) d920 Recreation and leisure (H.60) e150 Design construction and building products and technology of buildings for public use (H.81)	e310 Immediate family (H.77) e320 Friends (H.77) e355 Health professionals (H.79) e410 Individual attitudes of immediate family members (H.76) e420 Individual attitude of friends (H.76) e460 Societal attitudes (H.75) e535 Communication services, systems, and policies (H.80) e580 Health services, systems and policies (H.78)	b210 Seeing functions (H.8/H.9) b240 Sensations associated with hearing and vestibular functions (H.12/H.13/H.20) b250 Taste function (H.10) b255 Smell function (H.11) b280 Sensation of pain (H.14/H.15)	b126 Temperament and personality functions (H.1) b134 Sleep functions (H.2) b140 Attention functions (H.3/H.4) b144 Memory functions (H.5/H.6) b167 Mental functions of language (H.16/H.17) d160 Focusing attention (H.3/H.4)

The same applies to labels B, D, F, and G where, for instance, communication with others becomes more challenging, because of HL, the patient has difficulty interacting with people (included in the AP domain) and others are not supportive and enduring with the patient (included in EF domain). Similarly, categories b140 (attention functions) of the BF and d160 (focusing attention) of the AP domains correlated with items H.3 and H.4 were included in label G.

#### 3.2.2 Outcome score

To analyze the overall outcome of the HEAR-COMMAND tool, based on the extracted labels, three outcome scores were defined, which summarize the overall responses. As the responses to “voice and speech production functions” and “hearing aid benefits” items are needed only for a specific target population (speech-impaired participants and hearing aid users), they are excluded from the general score calculation.

To calculate the scores, 37 items included in labels A, C, and E, directing related to hearing and audiological aspects, were merged and named “hearing-related” items. The remaining 41 items included in labels B, D, F, and G which target non-audiological concepts and are not directly related to hearing, combined and named “non-hearing-related” items. Furthermore, to ease a direct comparison between the speech audiometry results performed by HCPs in the clinic with the self-assessment of speech perception, a subset of the hearing-related items including 32 items were selected to calculate a speech perception score. Included are items in which the target sounds are either exclusively speech (such as conversation in a restaurant) or sound in general with the following exceptions: Five items in label A in which the target sounds are non-speech are excluded including household noise, like running water or a washing machine (H.25), a car or a bus (H.26), music (H.27), a bus or a truck (H.29 and H.31).

Prior to calculating the outcome scores, the following mathematical adjustment was applied to the responses. In all items, the highest degree of responses is defined as profound/complete with a numeric value of 4 on the Likert scale. For items H.81 to H.86 corresponding to the impact of the environmental barriers, a profound/complete response reflects the most difficult or problematic situation which is in line with the BF and AP domains representation. However, for items H.75 to H.80 and H.87 to H.90 corresponding to the environmental facilitators, a profound/complete response means that the target concept, for instance, social support from family and friends, eases the situation which is in contrast with other items. Therefore, to conceptually align the responses of the environmental facilitators with all other items in the score mathematical calculation, the responses' numeric values to items H.75 to H.80 are “mirrored”. This means for these items, solely for the sake of the score calculation, a “profound/complete” response is assigned to 0, a “substantial” response is assigned to 1, a “mild” response is assigned to 3, and a “no” response is assigned to 4. Naturally, a “moderate” response remains as 2. By applying this numeric alternation, the response value of 4 always corresponds to the highest level of hardship in functioning.

As the “hearing-related” group includes 37 items, the highest possible hearing-related score (which represents the response of “4” to all the items) would be 148. Similarly, the highest possible non-hearing-related score corresponding to 41 items would be 164, and for 32 speech perception items, the score would be 128. The raw scores measured for each group were then normalized and represented out of 10. Normalization was performed by multiplying the raw score by the corresponding weighting coefficient. Representing the responses with normalized values has multiple advantages. 1. Normalization and representation of the responses out of 10 accommodate a straightforward comparison between the three calculated scores. 2. For participants, an overall view of their provided responses is easier to understand when presented out of 10 rather than raw scores out of 148, 164, and 128. 3. A research conductor (where the tool is used for research purposes) or an HCP (where the tool is applied in clinical evaluation) can more easily compare the performance of different participants and measure scores within the same normalized range. 4. Representing the tool's outcome with three values out of 10 facilitates decision-making processes, such as defining disability degree classifications.

The maximum possible normalized score (10 out of 10) represents the case that the participant chose the response option “4” on the Likert scale for all the items in a group. The minimum normalized score (0 out of 10) corresponds to the case that the response option “0” on the Likert scale was chosen for all items. All other raw scores fall within the range of 0–10. The coefficients are as follows: 0.067 for hearing–related, 0.061 for non-hearing-related, and 0.0782 for speech perception scores.

**Figure 2:**
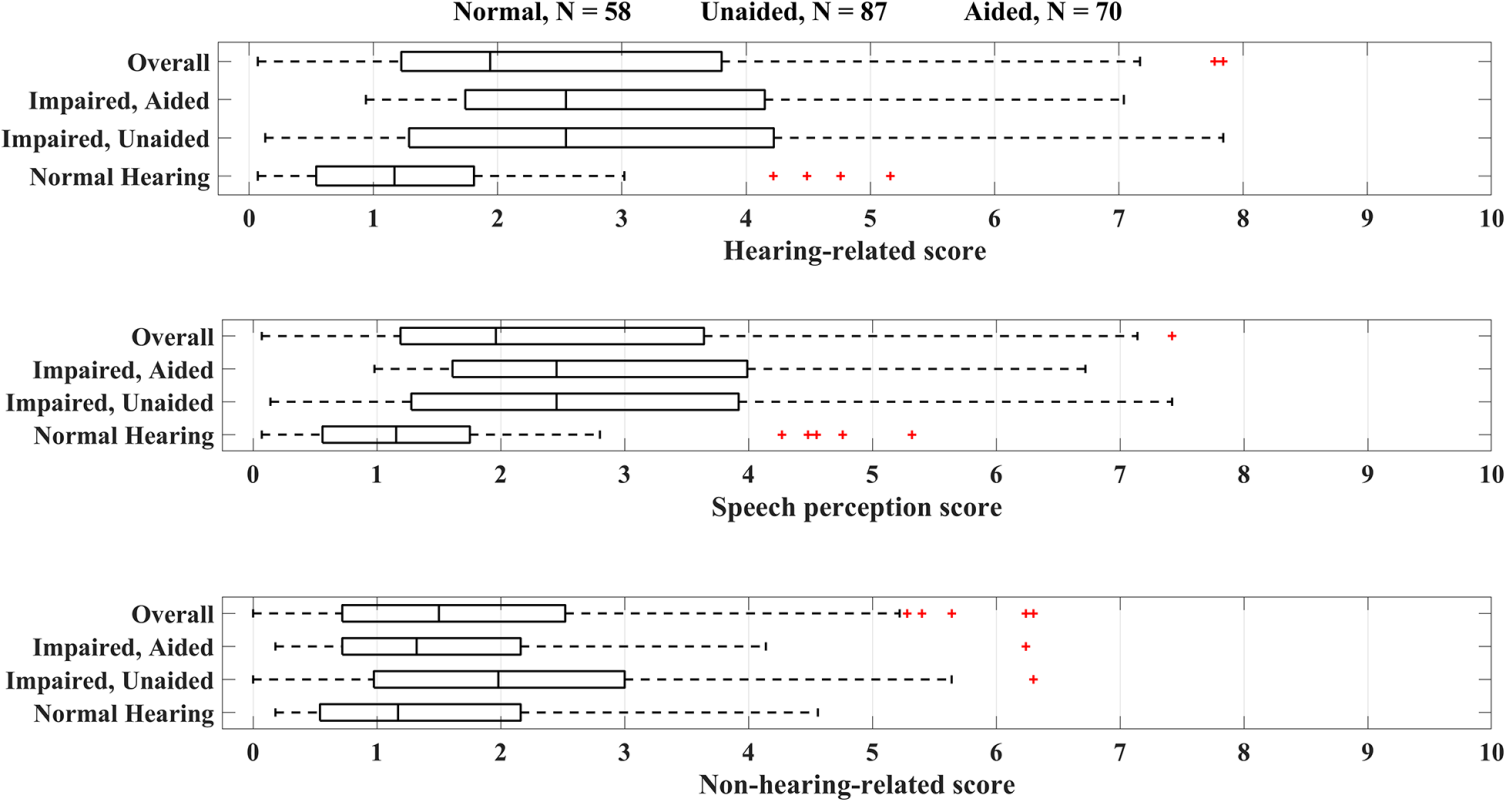
The distribution of the HEAR-COMMAND tool scores separated based on the PTA and hearing aid provision.

**Figure 3:**
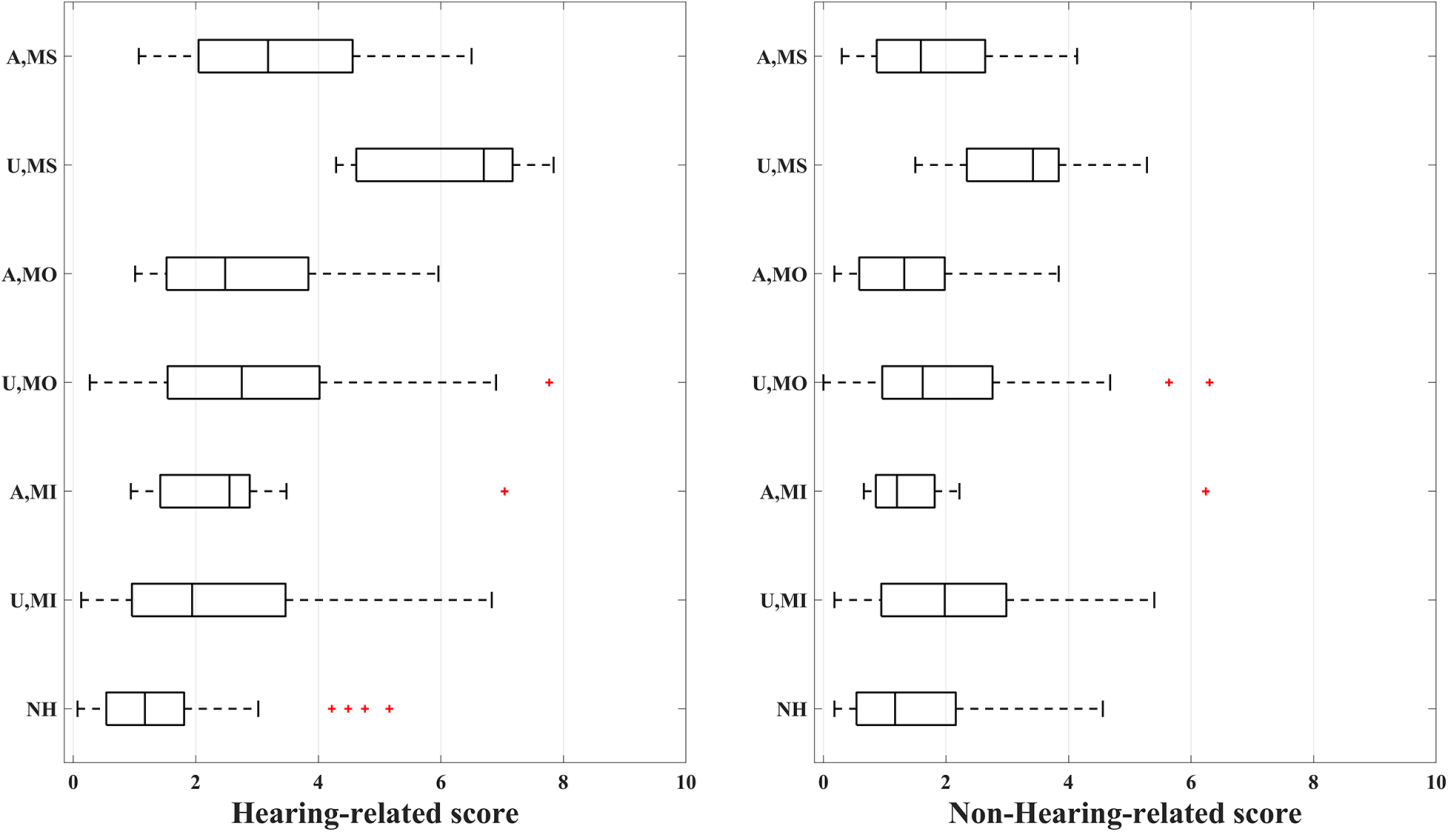
Hearing-related and non-hearing-related scores distribution for 7 subsets of the population, shown on y-axis. NH: normal hearing (*N* = 58), U,MI: unaided, mild (*N* = 47), A,MI: aided, mild (*N* = 9), U,MO: unaided, moderate (*N* = 34), A,MO: aided, moderate (*N* = 37), U,MS: unaided, moderately severe (*N* = 6), A,MS: aided, moderately severe (*N* = 24).

Figure 2 compares the scores measured for normal hearing, hearing-impaired unaided, and aided groups. As expected, normal hearing participants have significantly different speech perception and hearing-related scores in comparison with both unaided and aided groups at 95% Confidence Interval based on the Mann–Whitney *U* test (*p*-value = 0.001). Figure 3 shows the distribution of the hearing-related and non-hearing-related scores for each subset of the sample population based on their hearing status and HL degree. Analyses regarding the comparison within the hearing-impaired were not carried out, as it is difficult to compare patient collectives cross-sectionally due to confounding factors, e.g., type of device, treatment, and patient characteristics.

#### 3.2.3 Disability degree classification

The hearing-related and non-hearing-related scores can be further used to calculate the assigned HEAR-COMMAND tool disability degree following the WHO's recommendation for disability degree determination using a performance metric score as in ICF-based WHO's Model Disability Survey (MDS) (42, 43). MDS items are presented in distinct modules corresponding to different ICF domains as follows; Modules 1,000 (socio-demographic characteristics) and 2,000 (work history and benefits) which correspond to PF, module 3,000 corresponds to EF, module 4,000 corresponds to functioning, and 5,000 relates to health conditions. The response options are provided on a 5-point Likert scale. The wide range of EF categories found essential to include in MDS to determine the disability degree provides further proof that to broadly evaluate an individual's disability, using a brief Core set is not sufficient.

When interpolating the disability degree according to MDS and HEAR-COMMAND tools, two fundamental points must be carefully considered. 1. The concept of disability in both tools is defined according to a broad spectrum of influential parameters, rather than a single measurement of one aspect, such as defining the degree of hearing loss solely based on tone detection in quiet (as performed in pure tone audiometry). Therefore, it is expected that the disability degree defined based on the outcome of these tools does not necessarily align with the one derived from other methodologies which consider limited factors, commonly directly associated with physical conditions. 2. Mathematically, the suggested disability classification highly depends on the sample population's distribution, and the combination of the mean and standard deviation of the scores of the population is used to define the score range for each degree (No, Mild, Moderate, and Severe degree).

Table 6 shows the defined disability degree according to the HEAR-COMMAND tool measured scores using the mean and standard deviation derived from the distribution of the scores in this sample population. As highlighted, in this classification, the disability degree is defined by including the entire sample regardless of their critical differences, most importantly hearing status and age. The exclusion of a specific group of participants, such as participants below a certain age, potentially varies the distribution of the scores and consequently changes the derived cutoff values (mean and standard deviation). Furthermore, in the current evaluation, normal hearing participants, aided and unaided individuals with mild to moderately severe HL were included. As this population does not include all groups of individuals with HL (such as those with severe to profound HL or CI users), the results can potentially vary when samples of all individuals with HL are included.

**Table 6:** Disability degree thresholds based on the measured questionnaire outcome scores for normal hearing individuals and individuals with mild to moderately severe HL.

Disability degree	Measure	Speech perception score range	Hearing-related score range	Non-hearing-related score range
No	0 ≤ Score < M - SD	0≤ Score <0.7	0≤ Score <0.7	0≤ Score <0.5
Mild	M - SD ≤ Score < M	0.7≤ Score <2.4	0.7≤ Score <2.5	0.5≤ Score <1.8
Moderate	M ≤ Score < M + SD	2.4≤ Score <4.1	2.5≤ Score <4.3	1.8≤ Score <3.1
Severe	M + SD ≤ Score	4.1≤ Score	4.3≤ Score	3.1≤ Score

**Figure 4:**
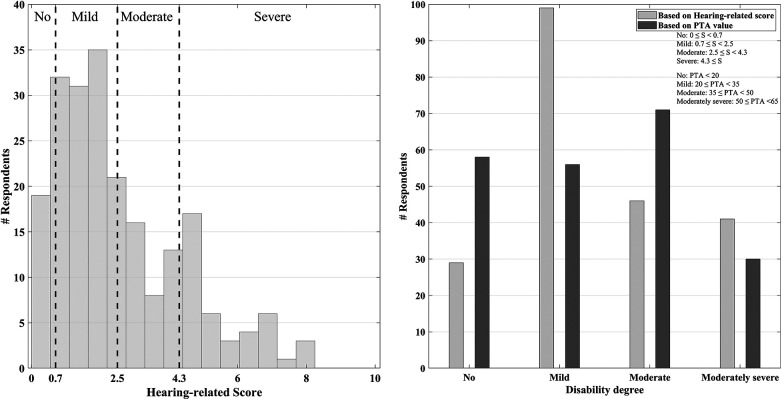
Left: the distribution of the absolute value of the hearing-related scores included in each disability degree. Right: the measured disability degree according to the hearing-related score and PTA values.

The hearing-related score and the derived corresponding disability degree can reveal the potential match and mismatch between the self-reported hearing impairment and an impairment classification solely based on pure-tone audiometry outcomes (PTA value) as a common measure of HL classification. This was observed for participants in all three countries at the individual level. For instance, the hearing-related score for a participant with a PTA of 60 dB HL was 4.5. In this case, self-reported and audiometry-based disability degrees are in line with each other. Another participant with a PTA of 30 dB HL is classified as an individual with mild HL according to the PTA value; however, his speech perception score was measured as 0.56, which is within the normal range. More such examples with detailed comparisons are provided in Supplementary Material S2, Table 3. Figure 4 demonstrates the distribution of the hearing-related score corresponding to each disability degree (left) and compares these degrees with those measured based on the PTA values over all the participants (right).

It was observed that according to the hearing-related scores, the number of participants with no HL is half of the number of participants with no HL, as indicated by the PTA value. Furthermore, the count of participants with mild HL according to hearing-related scores is twice the number of participants categorized as having mild HL based on the measured PTA values. This could mean this tool is more sensitive than the PTA value when revealing HL-related disabilities. The fundamental reason behind this mismatch is that, as mentioned above, while PTA represents an averaged value based on pure tone thresholds in quiet in a laboratory-controlled situation, the HEAR-COMMAND tool is founded based on a broad spectrum of influential factors as it's an ICF-based measure. The tool consists of multiple diverse scenarios in which the participant is required to consider hearing difficulty in conditions not simulated in PTA measurements, such as while localizing a sound or engaging in a conversation in a noisy auditory scene in real life. As the methods are based on different auditory contexts, the direct comparison of the assigned disability degree between the two methods may be prone to inaccuracies. This aligns with the concern expressed in the literature regarding the lack of widely accepted clinical methodologies capable of revealing hidden HL. This specifically applies to the individuals who experience hearing difficulties in daily life despite being classified as having no HL, solely based on the pure-tone audiometry results (44–46).

Furthermore, the comparative analysis of the PTA value and speech perception score revealed that there were participants who claimed high speech intelligibility despite their low measured PTA value, and on the contrary, some participants claimed low speech intelligibility while having a high PTA. This approach can be further expanded by comparing the outcome scores with additional laboratory-based assessments including speech reception audiometry with different combinations of target and masker sounds. This would allow the HCPs to discover and compare the difficulties claimed by the patient with the clinical approaches, potentially resulting in hearing aid fitting with higher efficiency.

### 3.3 Reliability

Table 7 summarizes Cronbach's alpha variable considering six different combinations of items. To include the “Hearing aid benefits” items and reach the maximum number of inclusive items, Cronbach's alpha was also measured once only based on the responses of the aided participants. As “voice and speech production functions” items only target speech-impaired participants and were responded to only by 5% of the population, it was excluded from this evaluation. As in all six proposed combinations, the alpha coefficient stood at 0.9, it can be concluded that a high level of internal consistency among the ICF-based items at different levels exists. This encompasses the overall reliability (combinations 5 and 6), when an ICF domain is solely evaluated (combinations 1 and 2), and when a group of items is considered (combinations 3 and 4). This further indicates that HCPs can highly rely on the outcome of employing the HEAR-COMMAND tool in the patient's disability assessment as it is derived from consistent and dependable responses to ICF-based items.

**Table 7:** The measured Cronbach's alpha variable in multiple item selection.

Combination #	Items selection	# items	*α*
1	Body functions (excluding voice and speech production functions)	40	0.958
2	Activities and participation	26	0.962
3	Hearing-related items	36	0.968
4	Non-hearing-related items	42	0.922
5	Hearing-related and non-hearing-related items combined	78	0.969
6	All items (excluding voice and speech production functions) *Based on the responses of aided participants	82	0.969

## 4 Discussion

This study was to validate the HEAR-COMMAND tool, recently developed in three languages for normal hearing individuals and individuals with mild to moderately severe HL. The validation and reliability assessment revealed that the tool was quantitatively and qualitatively a suitable measure of self-reporting hearing impairment and the corresponding daily functioning difficulties.

The low average non-gradable rate (4.2%) reveals that it was feasible for the participants to grade the content of the items on a scale of 0–4. The high mean responses of EF, facilitator items suggest a generally high satisfaction level with the necessary systems/services needed for daily functioning. Yet, the presence of the ceiling effect suggests that a broader scale, such as 0–10, might offer a chance to further differentiate the satisfaction level.

### 4.1 Contextual factors: personal factors

In the adult population, research has substantiated that HL typically emerges around middle age and progresses gradually over the years leading to hearing disability. However, hearing disability as comprehended by the ICF is the outcome of the interplay between an individual's health condition(s) (e.g., HL and co-morbid health conditions) and the physical, human-built, attitudinal, and socio-political environment in which they reside. It is not solely attributed to an internal characteristic resulting from impairments or health conditions. Accordingly, hearing disability on a continuum may range from no disability (or full functioning) to very high levels of disability, indicating that the nature and extent of disability cannot be directly deduced from health conditions alone. This neutrality ensures equality between disabilities arising from physical and mental health conditions, promoting a balanced perspective on the diverse manifestations of disability.

In our diverse cohort, the prevalence of diagnosed health conditions among participants varied, with hypertension and Diabetes Mellitus being the first and second most reported conditions, respectively. The lower hypertension prevalence rate in Egypt can be potentially the result of the sample's lower average age (40 years old). Furthermore, pain and tension in the neck area were reported by a noticeable number of the participants (16%). The impact of these co-morbid health conditions on hearing-related outcomes is well-documented in the literature (47–53). This is in line with the 2019 report on global hypertension morbidity rate in adults (32% in women and 34% in men) (54). According to the reports by the Robert Koch Institute (RKI), currently, 7.2%–9.9% of adults in Germany are diagnosed with Diabetes Mellitus (55) and 15.6% with neck and back pain (56). Therefore, to maximize full functioning, the management of these problems needs to be considered.

The existence of a history of excessive noise exposure, asymmetrical HL, and a track record of ear infection and middle ear surgery may serve as supplementary factors contributing to hearing disability. The potential impact of these factors during the development and validation of the Speech, Spatial, and Qualities of Hearing Scale (SSQ) questionnaire was reported (57, 58).

Furthermore, hearing aid users wait 10 years on average before getting help for experience of HL. But during that time, their communication and social interaction became more difficult, and isolation and psychological problems increased. Lack of awareness, limited access to healthcare services, and cost might be some of the barriers (59). Hence, the distribution of the sample population HL degree must be considered when interpolating the comparison between unaided and aided participants. In this sample, there are 47 unaided and 9 aided participants with mild HL, and 6 unaided and 24 aided with moderately severe HL. This unbalanced distribution of HL degrees directly impacts the hearing-related score and presents challenges in the comparative analysis of the hearing-impaired participants' scores. Furthermore, the direct comparison of the unaided and aided responses for an individual becomes relevant when the tool is used to evaluate treatment efficiency by comparing the responses prior to and after a treatment such as hearing aid fitting or cochlear implant operation.

### 4.2 Hearing-related items

The absence of floor effect for aided participants for the H.34, H.37, H.39. H.40, and H.71 which all correspond to hearing in a noisy environment potentially reflect a low subjective benefit and efficacy of hearing aids in such auditory scenes. These auditory events such as listening to someone in a busy restaurant (H.71) are examples of the so-called “Cocktail party effect” (60, 61), the instances where intentional listening becomes challenging in the existence of multiple auditory streams also for normal-hearing listeners. The participants' responses to items evaluating speech intelligibility in noise and auditory masking reflect the difficulties we hypothesized the tool could reveal. This strong alignment between expected and observed difficulties supports the validity of the HEAR-COMMAND Tool. The tool's ability to accurately capture these known challenges faced by individuals with HL confirms its utility and reliability as an effective measure in both clinical and research settings.

### 4.3 Non-hearing-related items

The prominently high mean values observed in the mental functions (chapter 1 of the BF domain) underscore the significance of specific items. Notably, item H.18, indicating a problem with ringing, beeping, roaring, or buzzing in the ears (ICF category: b240, sensations associated with hearing and vestibular functions), stands out with a relatively high mean of 1.6, securing the second position in the rankings among BF and AP items.

Subsequently, item H.2 (problem with sleeping), corresponding to ICF category b134 (Sleep functions) holds the fourth-highest mean value at 1.4. This finding aligns with the study conducted by Alfakir, et al. (6), which revealed that information on sleep functions was frequently identified in the records of patients with HL and tinnitus. Often, individuals with HL and distressing tinnitus may experience greater difficulty falling asleep. The transition from a relatively noisy daytime environment to the quietness of the bedroom can amplify the perception of tinnitus noises, making them more noticeable and potentially disrupting the sleep onset process. Like HL, it is well-established that sleep disruption negatively impacts cognitive functionality like attention and memory (62, 63).

The 8th and 12th ranked means (1.3 and 1.1 respectively) correspond to memory functions; remembering things and recalling new information (H.5 and H.6, ICF category: b144). The 10th highest mean (1.2) belongs to H.4 (maintaining focus on two or more things at the same time, ICF category: b140). Accordingly, considerable attention should be given to sleep problems in people with HL and tinnitus.

The results from the EFA unveiled that item H.49, focusing on dealing with stressful situations, exhibits a loading of 0.55 on Factor 2 (labeled as B: Interpersonal Interaction functionality and infrastructure accessibility) and 0.36 on Factor 7 (labeled as G: Cognitive Functionality). These loadings suggest a relationship between cognitive factors and the challenges individuals face when dealing with stressful situations during the individual's social life. The loading on these two factors underscores a more significant association of individuals' ability to manage stress with social and cognitive functioning. This finding provides valuable insights, contributing to a more nuanced understanding of the impact of cognitive factors on social interactions and coping mechanisms within the studied population.

### 4.4 Countries differences

Hypothesis testing or statistical data analysis, e.g., for efficacy studies, requiring power analysis and sample size considerations has not been performed. The focus of this study is on content reliability and factor analysis to determine how the responses represent the performance of the participants. As a result, power analysis and minimum sample size calculation are unnecessary for these analyses. However, in comparison with similar studies, as stated in Table 1, the HEAR-COMMAND Tool was validated with the largest sample size with an overall of 324 participants. Furthermore, the items were answered by the participants in their native language, ensuring a higher level of reliability and content validity.

A straightforward and precise comparison between the responses of different countries requires highly similar population characteristics including the sample size, age, hearing status, and PTA value distribution. This study was conducted to validate the tool in multiple languages and did not intend to use the tool to reveal cultural differences based on the ICF framework. However, in general, some items of the tool are more likely to reveal cultural differences than others, especially those categorized in label B (Interpersonal Interaction functionality and infrastructure accessibility). For instance, the responses to H.59 (joining in community activities) had a similar distribution in Germany and the USA (90% and 86% no or mild difficulty, respectively). However, 38% of the participants in Egypt claimed that they have no or mild difficulty engaging in the community. A comparable pattern was observed in the responses to H60 (engaging in any hobby or pleasurable activity) and H.61 (continuing relationships in an appropriate manner). The current validation serves as a starting point and a roadmap for the developers in each country to further correlate the outcome of the tool with the cultural characteristics of the nation and ecological elements. A detailed comparison of the responses to each personal factor item can be found in Supplementary Material 2, Figures A.1–A.30.

### 4.5 Potential application extensions and future directions

#### 4.5.1 The electronic version of the HEAR-command tool

For further adaptability and accessibility of the HEAR-COMMAND tool, an electronic version of the tool has been developed to be applied in future studies. The healthcare provider can send the questionnaire link to the patient upon scheduling the appointment, allowing the patient to complete it before coming to the clinic to ensure that the completion of the questionnaire does not lengthen the duration of the clinical visit. Filling the electronic adaptive version reduces the time needed to fill out the tool as a negative response to filter questions (H.41, A.9, A.11, A.14, A.16, A.23, and A.27) shortening the number of required items for each participant. The electronic version allows the experts to target a larger population and focus on a broader demographic for further studies and collect feedback from participants from other countries. This also eliminates the missing responses as skipping an item is not allowed while responding to the items. In addition, an electronic version would automatically calculate the outcome scores including hearing-related, non-hearing-related, and speech perception.

#### 4.5.2 HEAR-COMMAND tool in other languages

Given that the tool is built on a global standard and has already been validated in three languages, there is a persistent interest and request to make the tool available in additional languages. This development underscores the ongoing commitment to expanding the tool's accessibility to diverse linguistic communities. The online platform hosting the tool is anticipated to seamlessly integrate these translations, ensuring that individuals from different language backgrounds can benefit from a standardized and comprehensive assessment of hearing health-related factors. This initiative aligns with the commitment to inclusivity and global applicability, recognizing that hearing health is a universal concern that transcends linguistic and cultural boundaries. By providing the tool in multiple languages, the aim is to enhance its usability and relevance for a more extensive and diverse user base, ultimately contributing to a more comprehensive understanding of the impact of HL on individuals worldwide (64). Recently, the tool was translated into Korean at Auburn University's ABILITY Research Lab and the validation process is currently underway.

#### 4.5.3 HEAR-COMMAND tool and other ICF core sets

To enhance the holistic approach to HL and further assess the validity of the HEAR-COMMAND tool items, contributing to a more comprehensive understanding of the impact of other related health conditions including dizziness and vertigo is essential. To do so, an ongoing data collection procedure is occurring at the ABILITY Research Lab at Auburn University. This process involves evaluating an additional 13 categories, represented by 15 items, adopted from the ICF Core Set for Dizziness and Vertigo (65, 66).

#### 4.5.4 Further validation and applications of the HEAR-command tool

The tool can be further validated by targeting other patients including those with profound or complete HL and cochlear implant users. As a result, it is expected that some items might be found unnecessary for a certain group of patients or have different correlation degrees and can be removed from the body of the tool, reducing the time that it takes to fill out the tool. The validation of the tool among cochlear implant users is currently ongoing and involves comparing the hearing and functioning status before and after the cochlear implantation operation.

The reported validity analyses are based on a cross-sectional approach to show the holistic facets of the ICF concept as a starting point for future applications and studies. Firstly, it was essential to show that normal-hearing people differed from hearing-impaired people as proof of content validity. For the usage in ENT and hearing acoustics with audiological and rehabilitation-specific questions relating to PRO, studies are needed that demonstrate the change sensitivity of the HEAR COMMAND tool. This could be established with prospective, longitudinal measurements. Future areas of application could include, e.g., acclimatization to hearing aids during the course of rehabilitation, evaluation of the achievement of rehabilitation goals, and benefits of fitting hearing aids and cochlear implants due to defined features of pre-processing.

Furthermore, the tool would be suitable for measuring the progress in the context of aural and non-aural rehabilitation of CI fitting or upgrades with speech processors. For the latter, the usage of the HEAR-COMMAND tool as a reference in procedures of Ecological Momentary Assessment (EMA, see 67) would be fruitful to show the effectiveness in contrast to efficacy measurements. In ongoing studies, an ICF-based short survey is implemented in an EMA mobile app (see 68 as an example) aiming at collecting subjective data in real-life scenarios for individuals with HL. This would allow real-time functioning and communication assessment. In similar scenarios, such as speech intelligibility in noise, the outcome of the HEAR-COMMAND tool can serve as a reference and be compared with multiple times that the EMA app was initiated over a specific period.

Performing incremental validity is beyond the scope of this publication. However, since the items of the shortened version (12 items) of the SSQ questionnaire (57, 58) are included in this tool (with terminology modification and adjustment where needed), a direct comparison of the hearing-related items and the SSQ score would be feasible (20).

### 4.6 Study limitations

In this study, 73% of the included participants were individuals with mild to moderately severe HL. It is important to note that this study represents the results of the first step in validating the tool, and it was not intended to encompass a sample representing the entire population of patients with HL. However, patients with mild to moderately severe HL who currently use or are candidates for hearing aids constitute a substantial portion of the HL population. Nevertheless, it is essential to include patients with severe to profound HL and those utilizing other hearing assistive devices including CI or Bone-Anchored Hearing Aids (BAHA) to broadly represent the tool's capabilities. To accurately compare cultural differences across the countries using the tool, it is crucial to have a harmonized sample across the countries, particularly in terms of age distribution and the number of participants in each HL category.

Patient categorization was based on the PTA values, however, incorporating additional assessments to define the degree of HL, including speech reception tests, can lead to a more precise classification. A Speech reception test was performed for 73 of the participants in Germany; however, as the outcome of such a test was not available for the entire sample, it could not be encountered as an additional classification factor.

A person's attitude toward hearing aids is closely linked to their usage. With modern hearing aids, the usage pattern can be obtained from the data logging feature. This data has not been recorded in this study. Future studies should examine the effect of hearing aid usage habits on functioning and hearing improvement.

## 5 Conclusion

This study aimed to validate the HEAR-COMMAND tool across three languages and evaluated the responses of the sample population in three countries to demographic questions and ICF-based items. The resultant scores including hearing-related, non-hearing-related, and speech perception scores can be directly used by the HCPs to derive the hearing and functioning disability degree based on the patient's perspective. The high reliability, the feasibility of grading the items, and the validation outcome implicate the compatibility of the usage of the tool with the intended design purpose to be used as a tool that reveals HL and the corresponding daily functioning challenges. The robustness of the tool design and its broad perspective allows researchers and HCPs to systematically evaluate a wide spectrum of an individual's needs and desires, enhancing the everyday effectiveness of the provided medical services including the treatment and rehabilitation procedure.

## Data Availability

The original contributions presented in the study are included in the article/Supplementary Material, further inquiries can be directed to the corresponding author.

## Footnotes

^1^HEAR-COMMAND stands for HEARing and COMMunication AND conversation disability.

^2^According to this classification, one of them can be included in the "Unilateral" category as in this case, the PTA of the better ear was less than 20 dB HL, and the PTA of the worse ear was above 35 dB HL. In the remaining cases, the PTA value of the better ear was above 20 dB HL, therefore they cannot be included in this category, and they were included in a category according to the PTA of the better ear.
