# Supplementary material for: ICF-based hearing and functioning assessment: validation and research outcomes of utilizing the HEAR-COMMAND tool for patients with mild to moderately severe hearing loss and individuals with normal hearing
Source: Front Rehabil Sci. 2024 Aug 26;5:1389653. doi: 10.3389/fresc.2024.1389653 (PMC11381501; doi:10.3389/fresc.2024.1389653)
Supplement: Supplementary file 2 [file Datasheet2.pdf]

frontiers

Supplementary Material (2)

File content: Pages 2 to 32 demonstrate the responses to Personal Factors and demographic items of the HEAR-COMMAND tool (A.1 to A.30) for each country (Germany, the USA, and Egypt) as well as the overall population. Pages 33 to 37 summarize the outcome of performing pure-tone audiometry. Pages 38 to 40 correspond to the overall responses to ICF-based items. Page 41 corresponds to detailed explanation of items with large non-applicable rate and the sample outcome of the tool for individuals.

List of content:

Item #	Concept		Page #
Figure A.1:	Gender	………………………………	2
Figure A.2:	Age	………………………………	3
Figure A.3:	Marital status	………………………………	4
Figure A.4:	Current occupation	………………………………	5
Figure A.5:	Years of education	………………………………	6
Figure A.6:	School for deaf students	………………………………	7
Figure A.7:	Current living situation	………………………………	8
Figure A.8:	Medical diagnosis	………………………………	9
Figure A.9:	Firearm usage	………………………………	10
Figure A.10:	Wearing hearing protection	………………………………	11
Figure A.11:	Exposure to noise at work	………………………………	12
Figure A.12:	Time duration	………………………………	13
Figure A.13:	Wearing hearing protection	………………………………	14
Figure A.14:	Exposure to noise out of work	………………………………	15
Figure A.15:	Wearing hearing protection	………………………………	16
Figure A.16:	knowing cause of hearing loss	………………………………	17
Figure A.17:	Main cause of hearing loss	………………………………	18
Figure A.18:	Sudden hearing loss	………………………………	19
Figure A.19:	Surgical treatment	………………………………	20
Figure A.20:	Middle ear infection	………………………………	21
Figure A.21:	Having runny ear	………………………………	22
Figure A.22:	Time hearing was tested	………………………………	23
Figure A.23:	Family history	………………………………	24
Figure A.24:	Family side	………………………………	25
Figure A.25:	Exact relationship	………………………………	26
Figure A.26:	Ears comparison	………………………………	27
Figure A.27 (1):	Wearing hearing aid	………………………………	28
Figure A.27 (2):	Which ear	………………………………	29
Figure A.28:	Hearing aid type	………………………………	30
Figure A.29:	Usage history	………………………………	31
Figure A.30:	Usage time	………………………………	32
Figure P.1:	PTA better ear	………………………………	33
Figure P.2:	PTA worse ear	………………………………	34
Figure P.3:	PTA difference	………………………………	35
Figure P.4:	PTA average	………………………………	36
Figure P.5:	Hearing status	………………………………	37
Table 1:	ICF-based items responses summary	………………………………	38
Table 2:	Non-gradable rate explanation	………………………………	41
Table 3:	Samples of tool outcome	………………………………	41

Figure A.1: Question 1: Gender

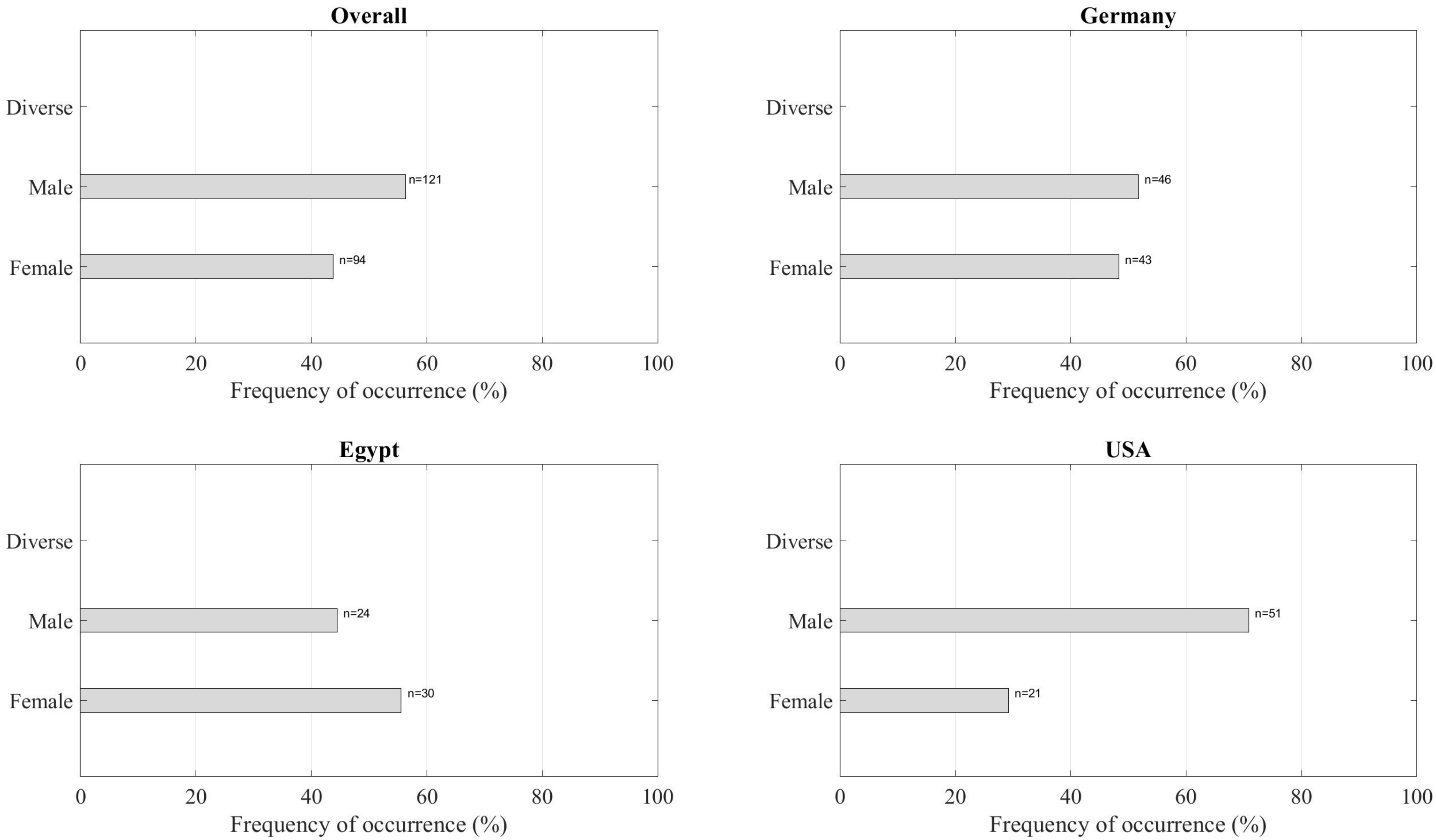

Figure A.2: Question 2: Age

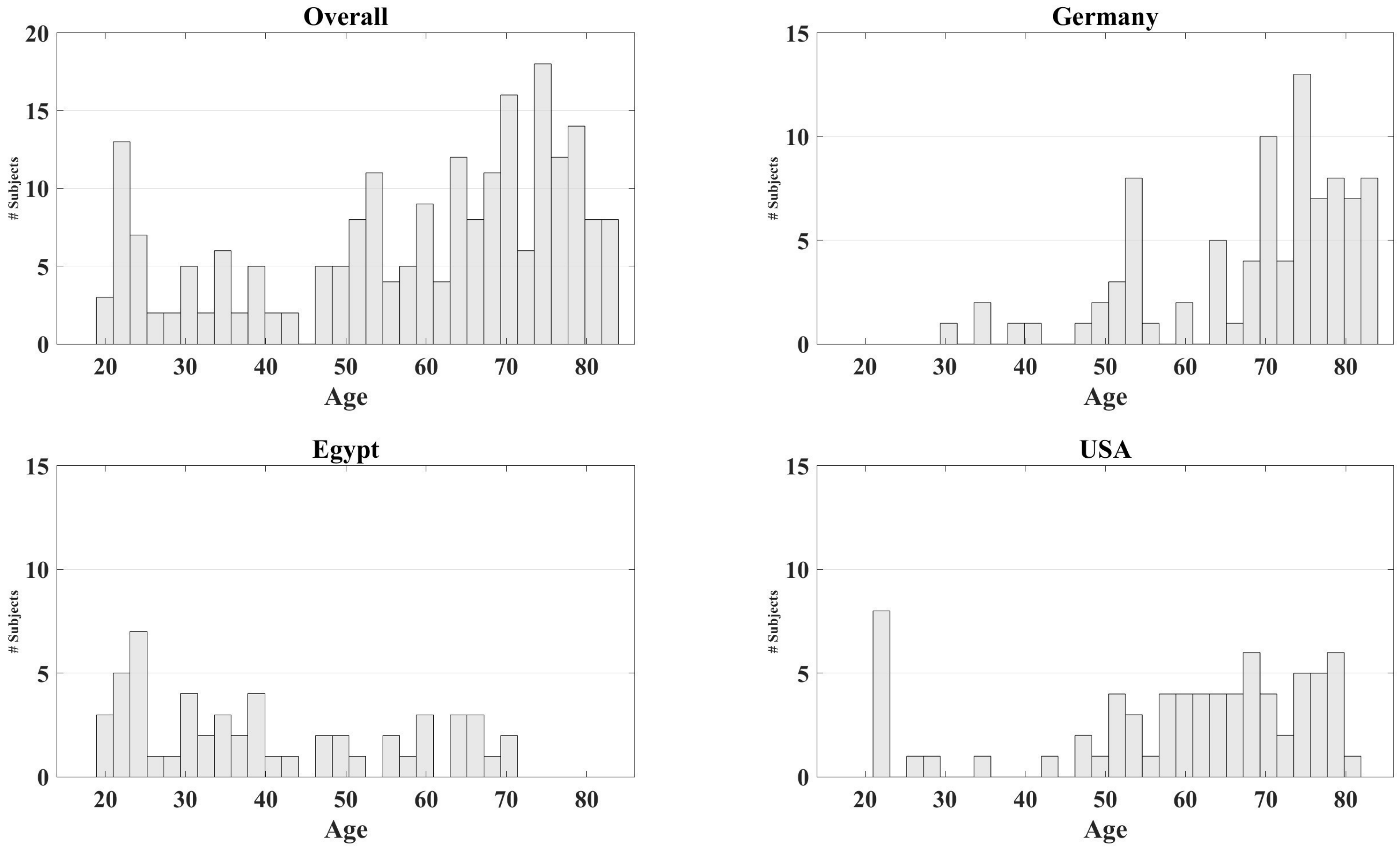

Figure A.3: Question 3: Marital status

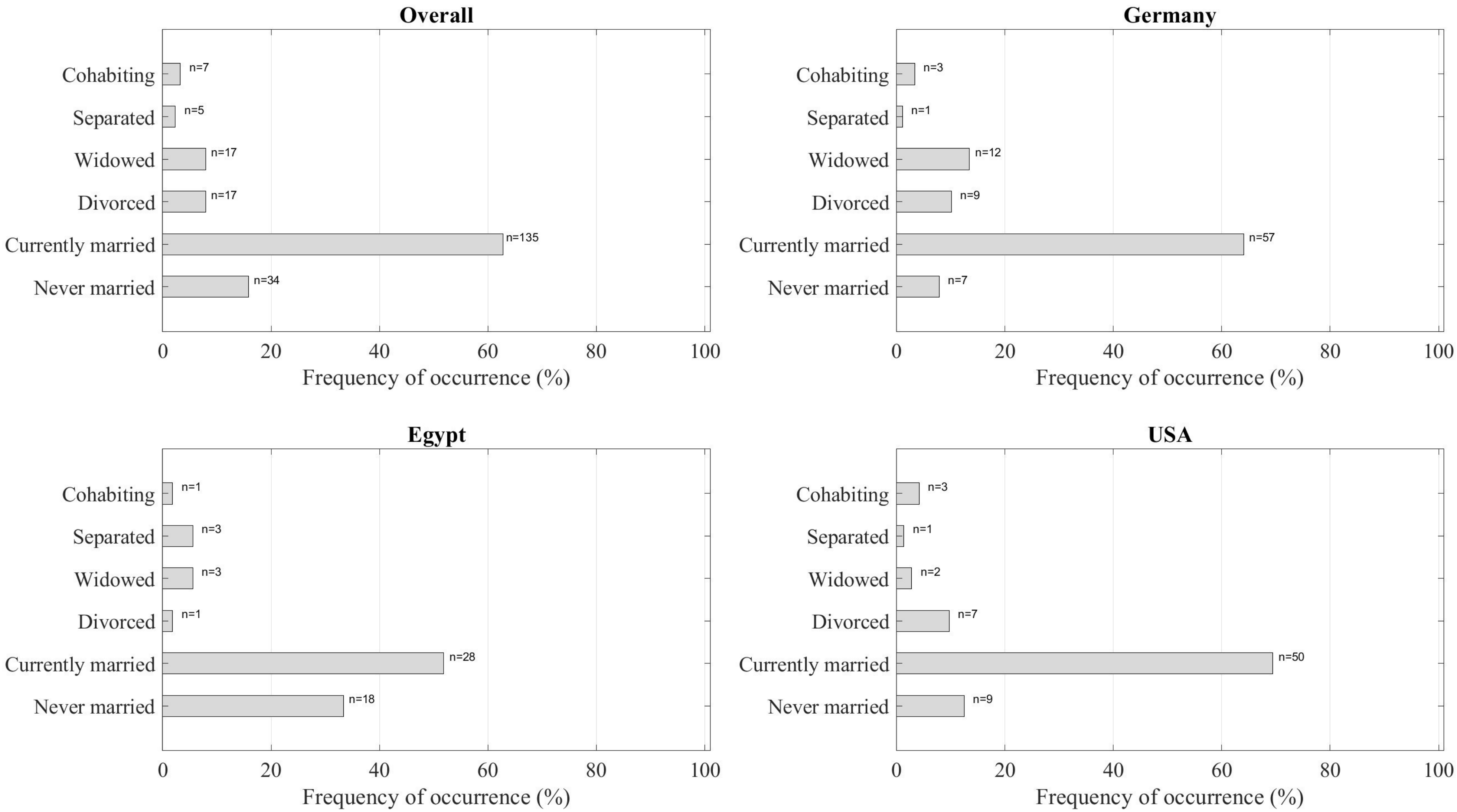

Figure A.4: Question 4: Current occupation

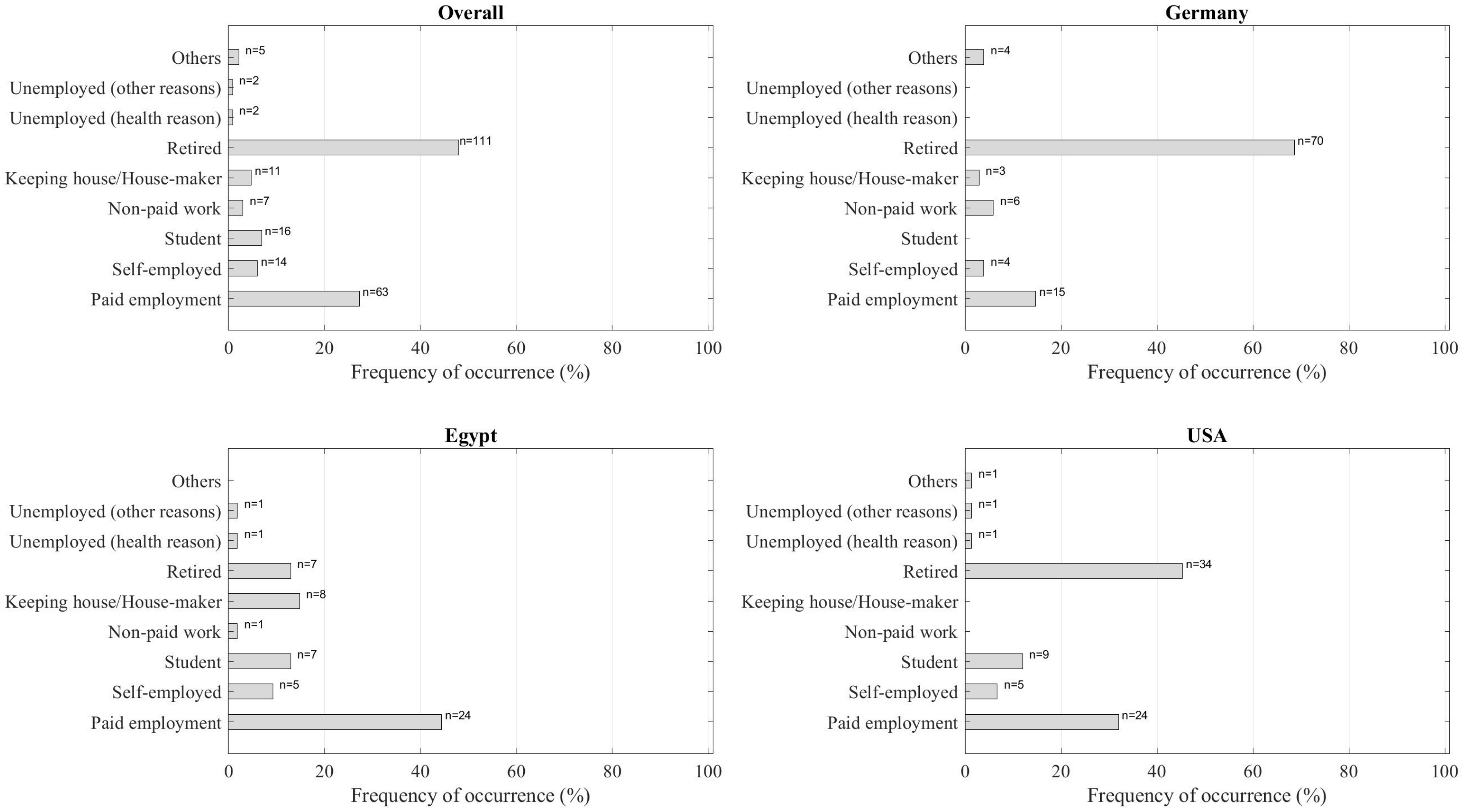

Figure A.5: Question 5:Years of education

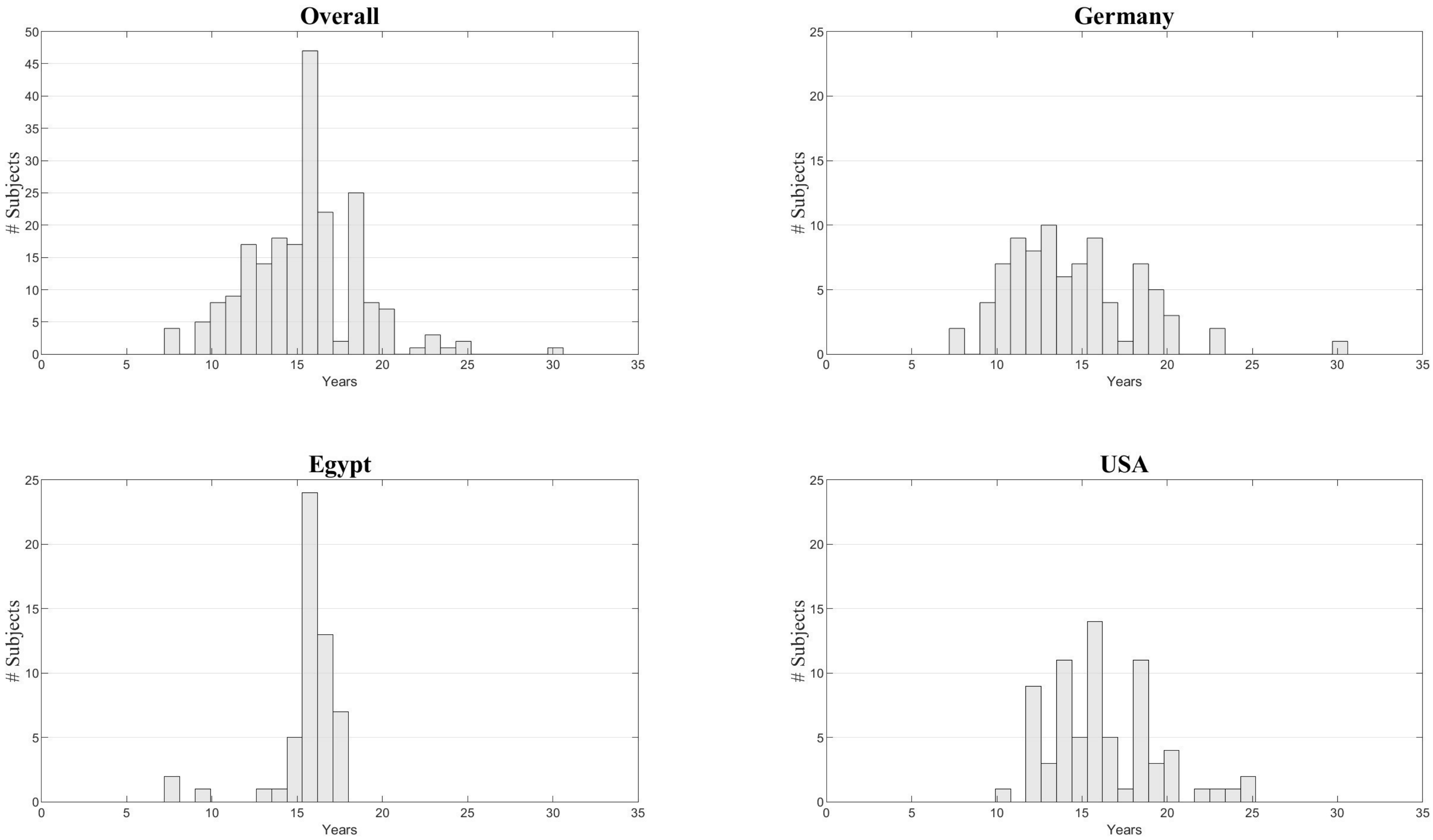

Figure A.6: Question 6: Attended a school for the deaf / hearing impaired?

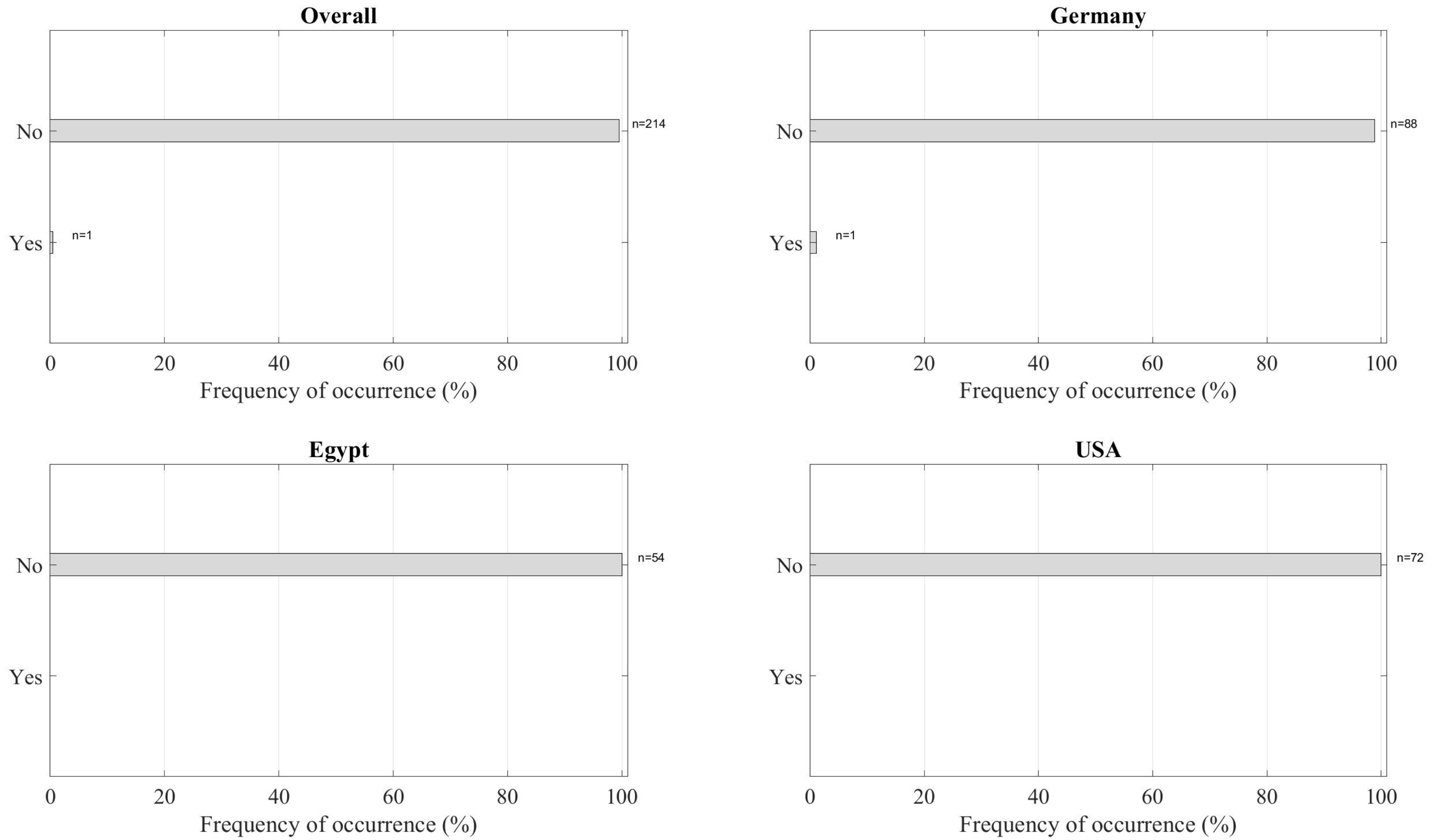

Figure A.7: Question 7: Current living situation

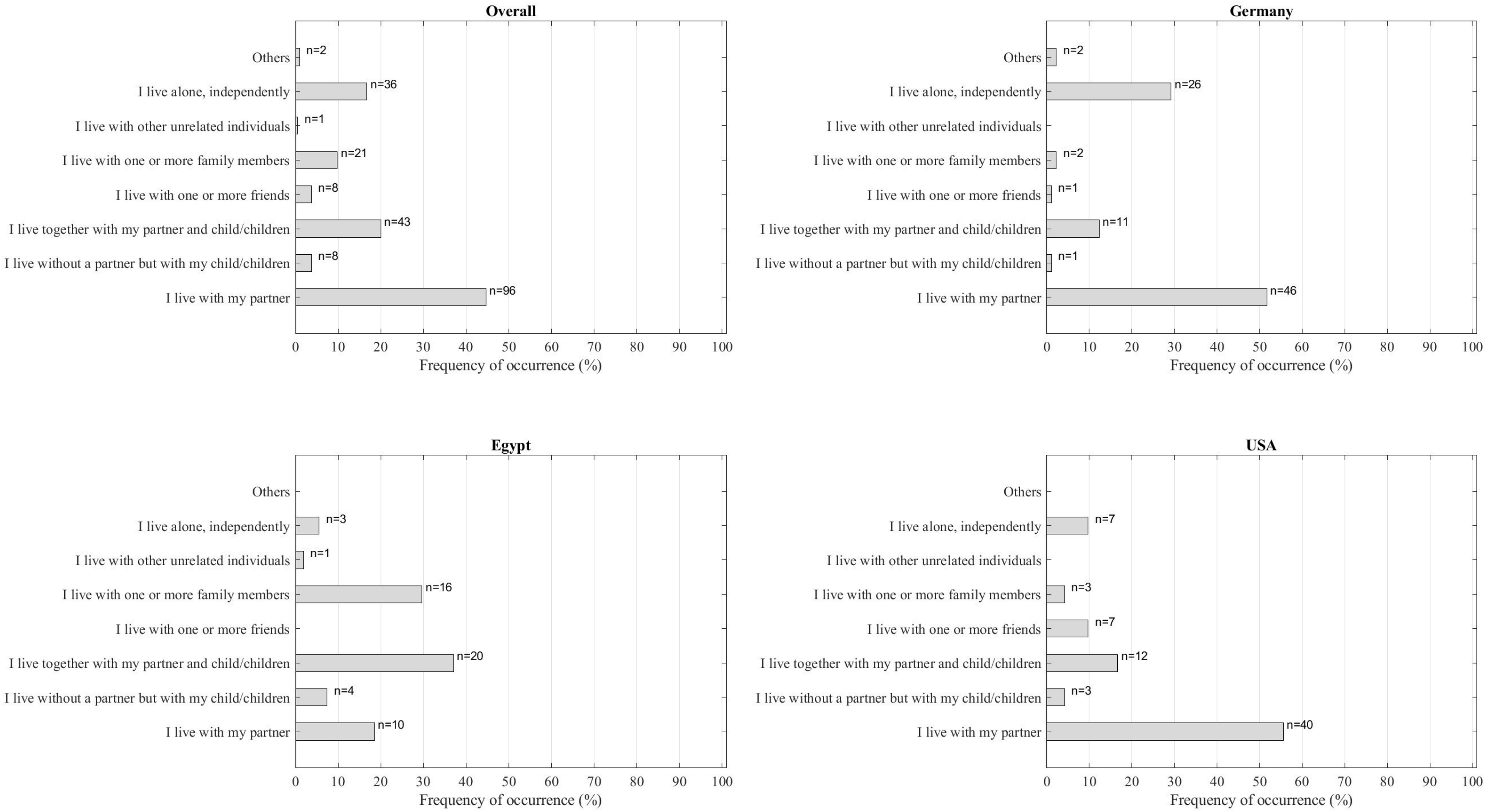

Figure A.8: Question 8: Medical diagnoses

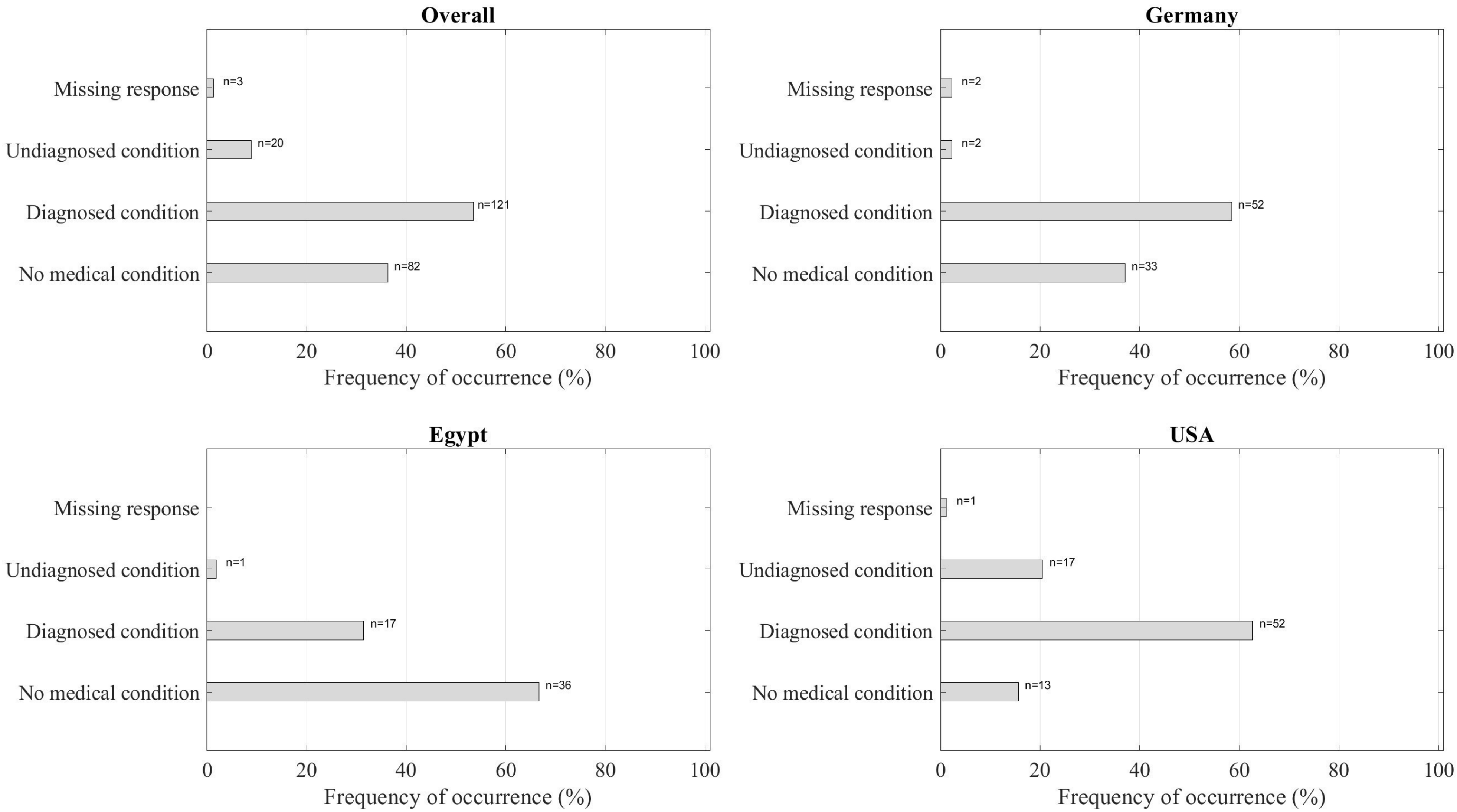

Figure A.9: Question 9: Firearms usage

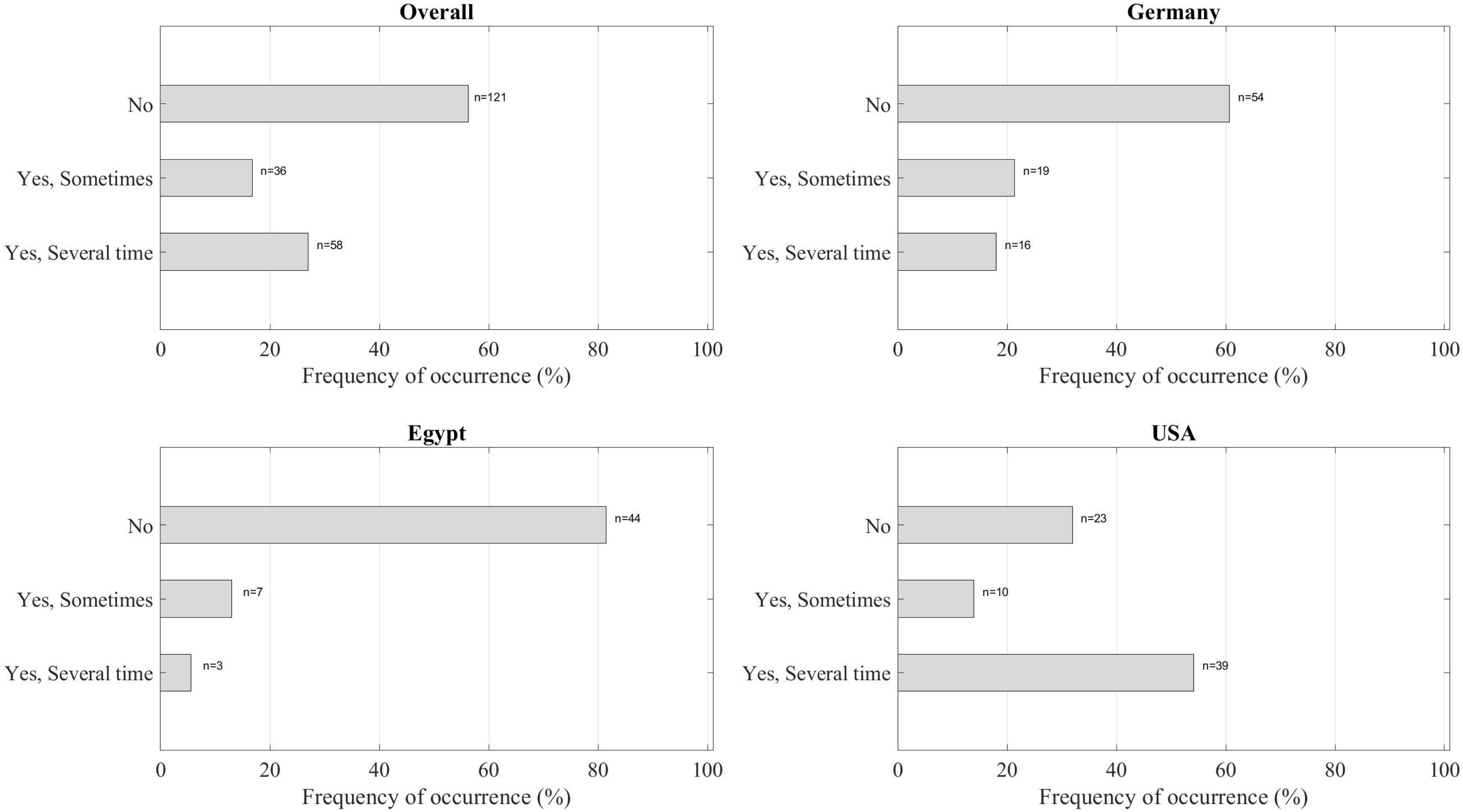

Figure A.10: Question 10: Wearing hearing protection while firearms usage?

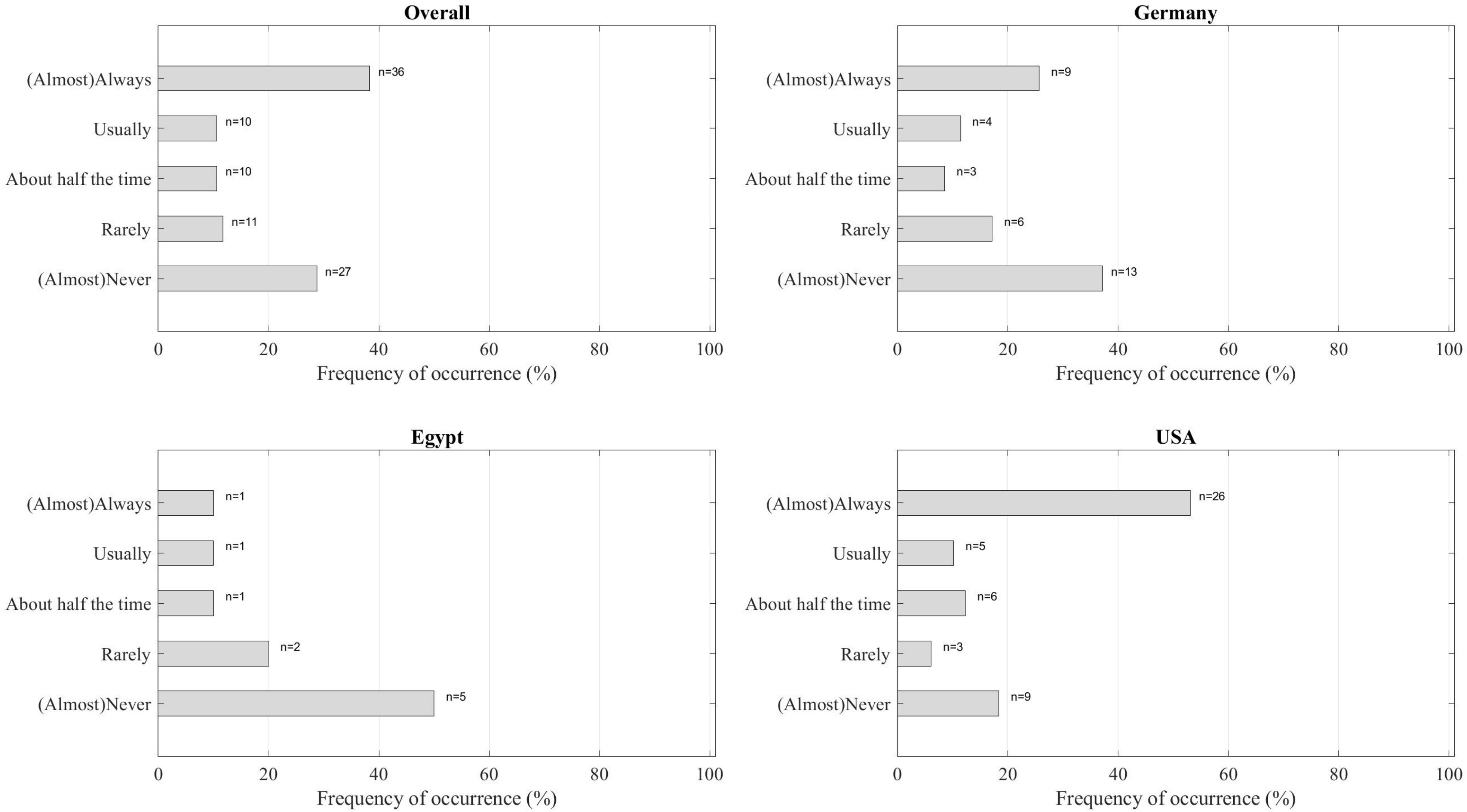

Figure A.11: Question 11: Exposure to loud noises at work?

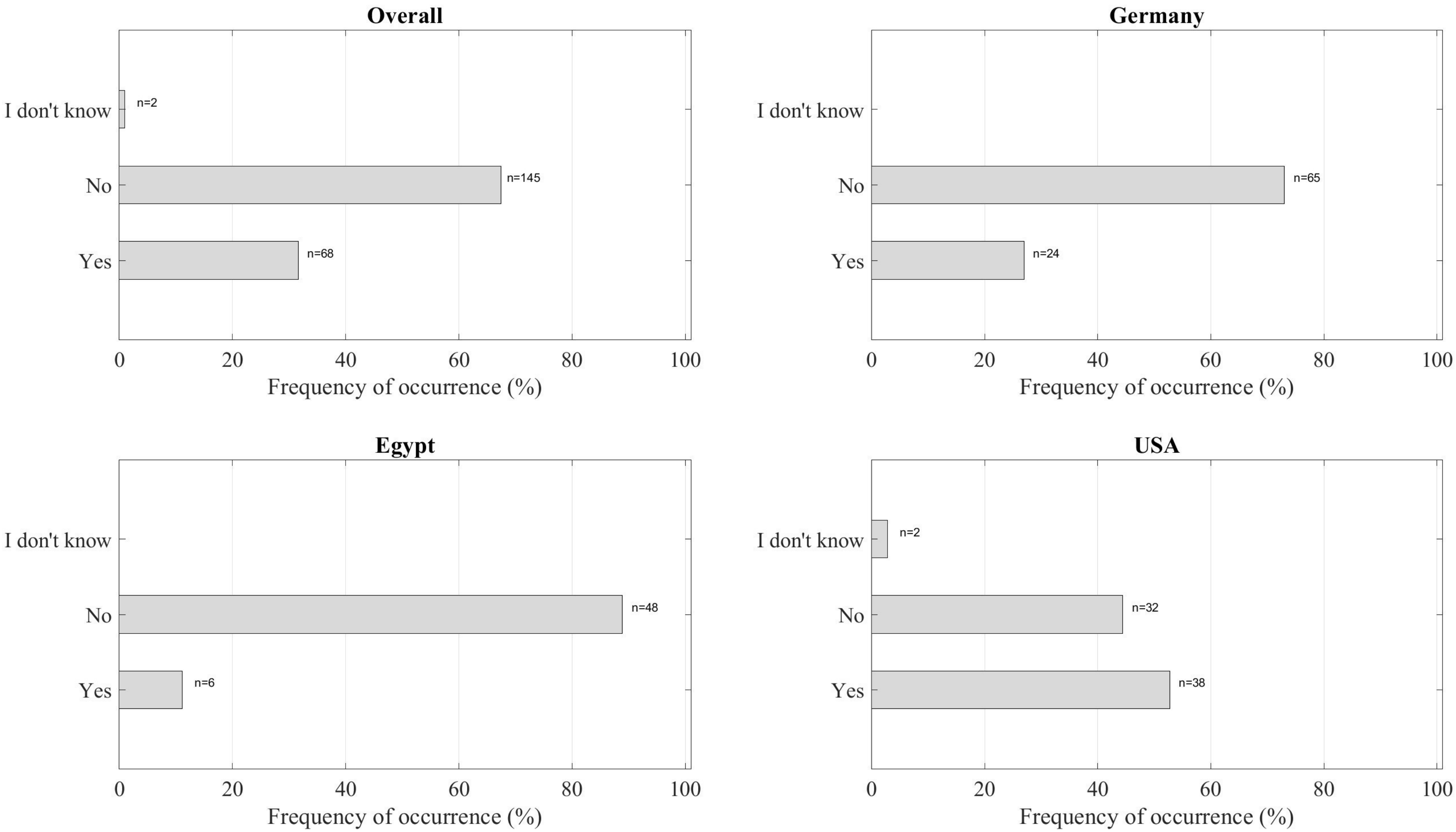

Figure A.12: Question 12: Loud noises at work / Time duration?

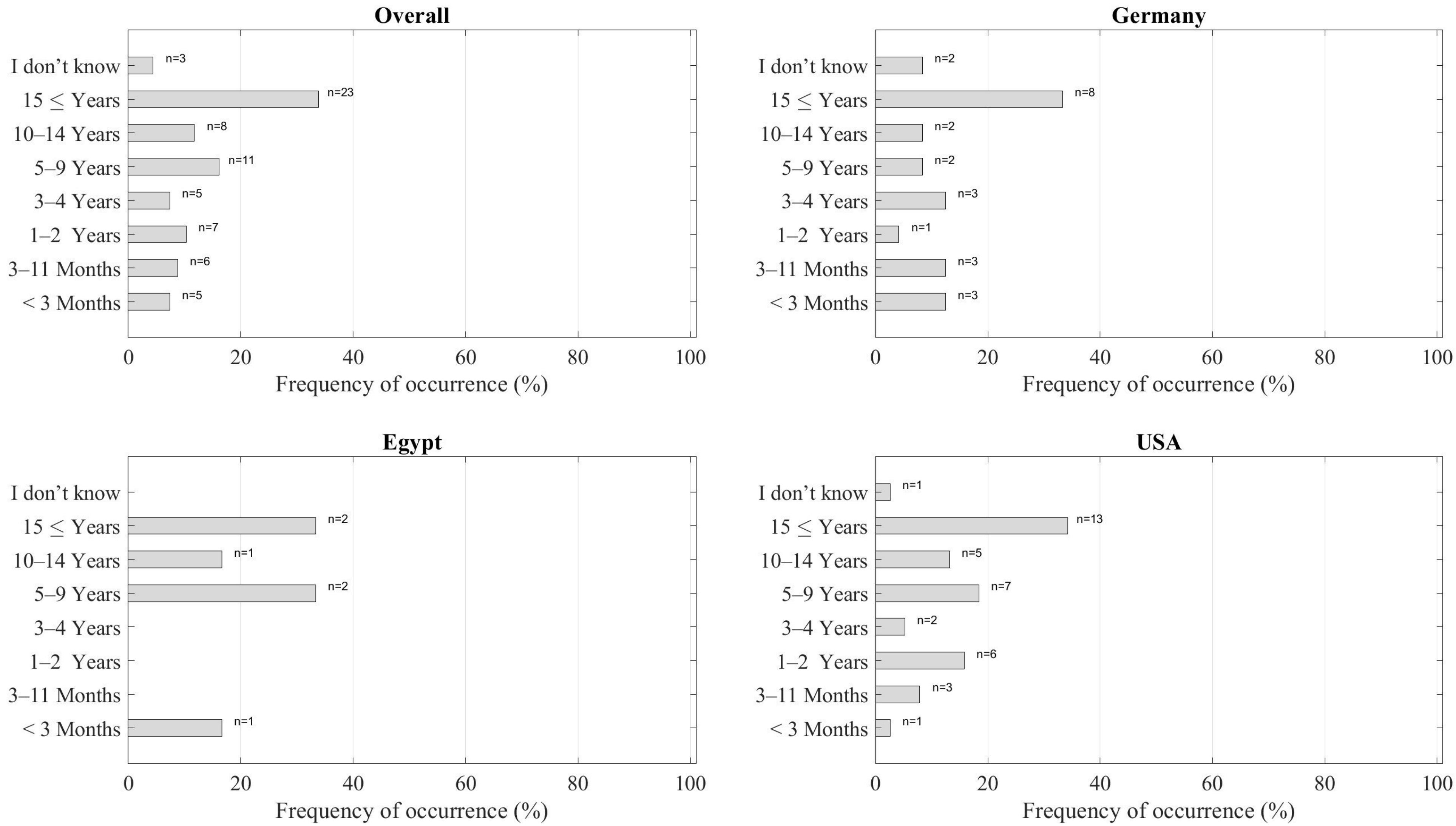

Figure A.13: Question 13: Loud noises at work / Worn hearing protection?

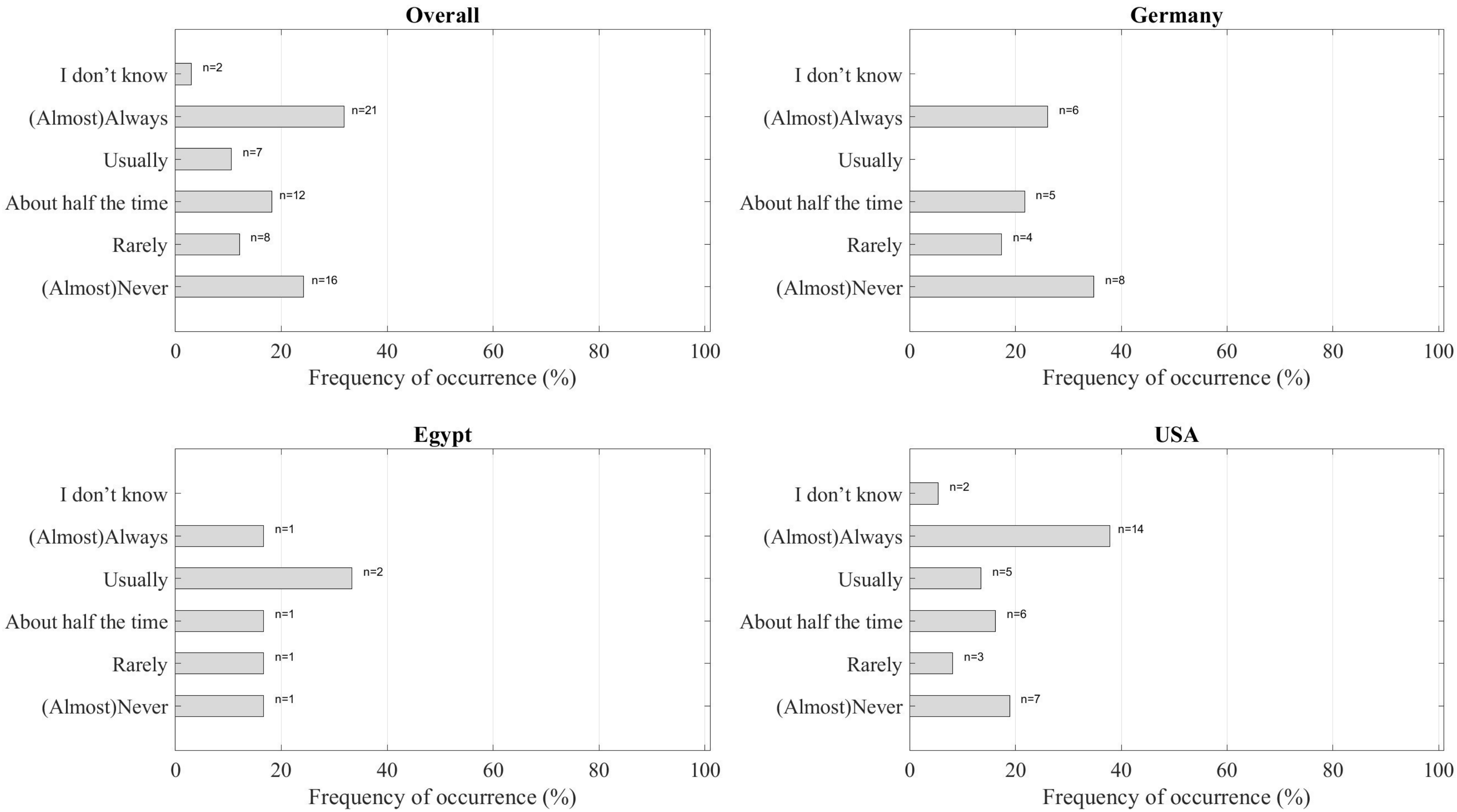

Figure A.14: Question 14: Exposure to loud noises outside of your job

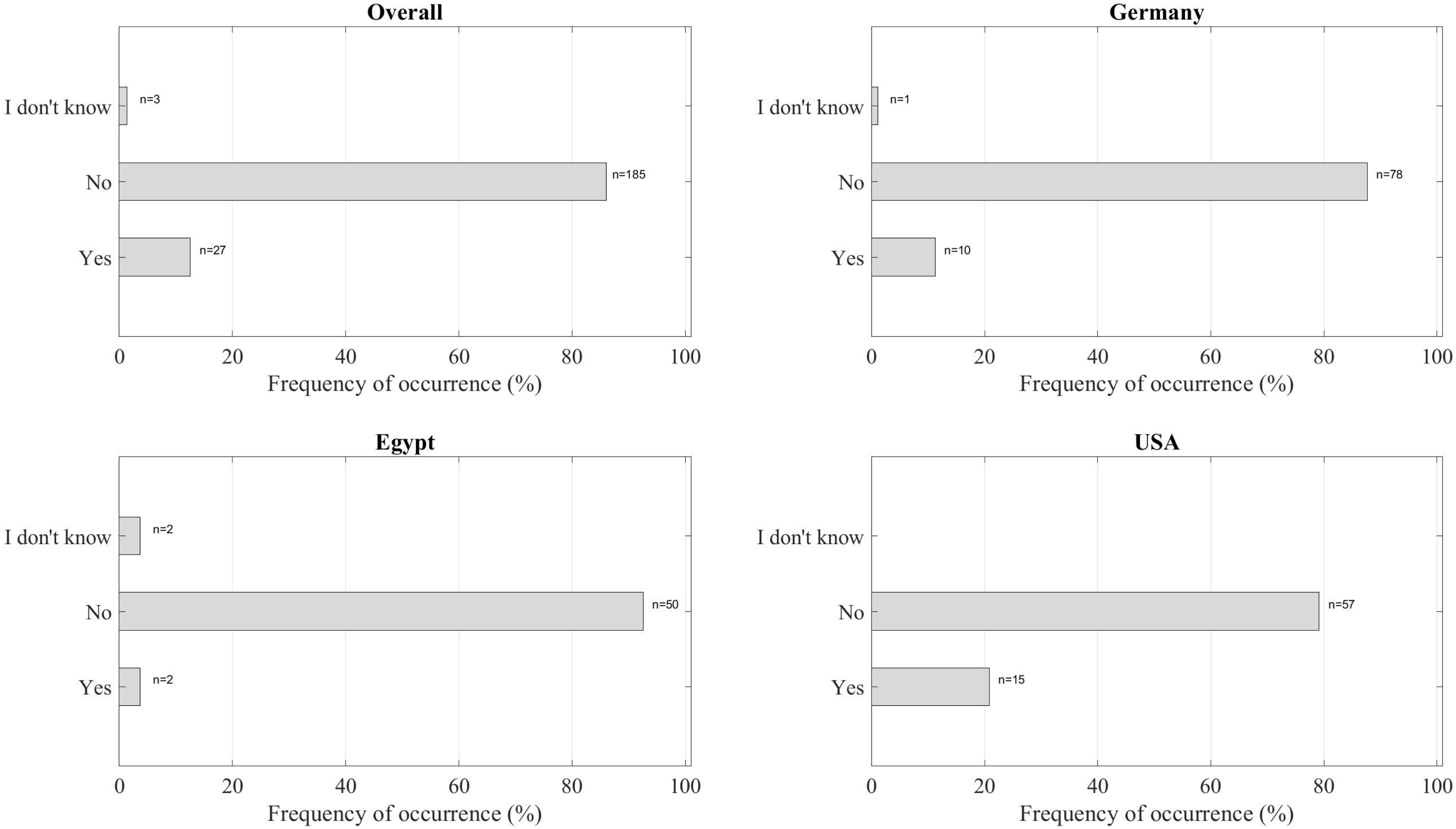

Figure A.15: Question 15: Loud noises outside of job / Worn hearing protection?

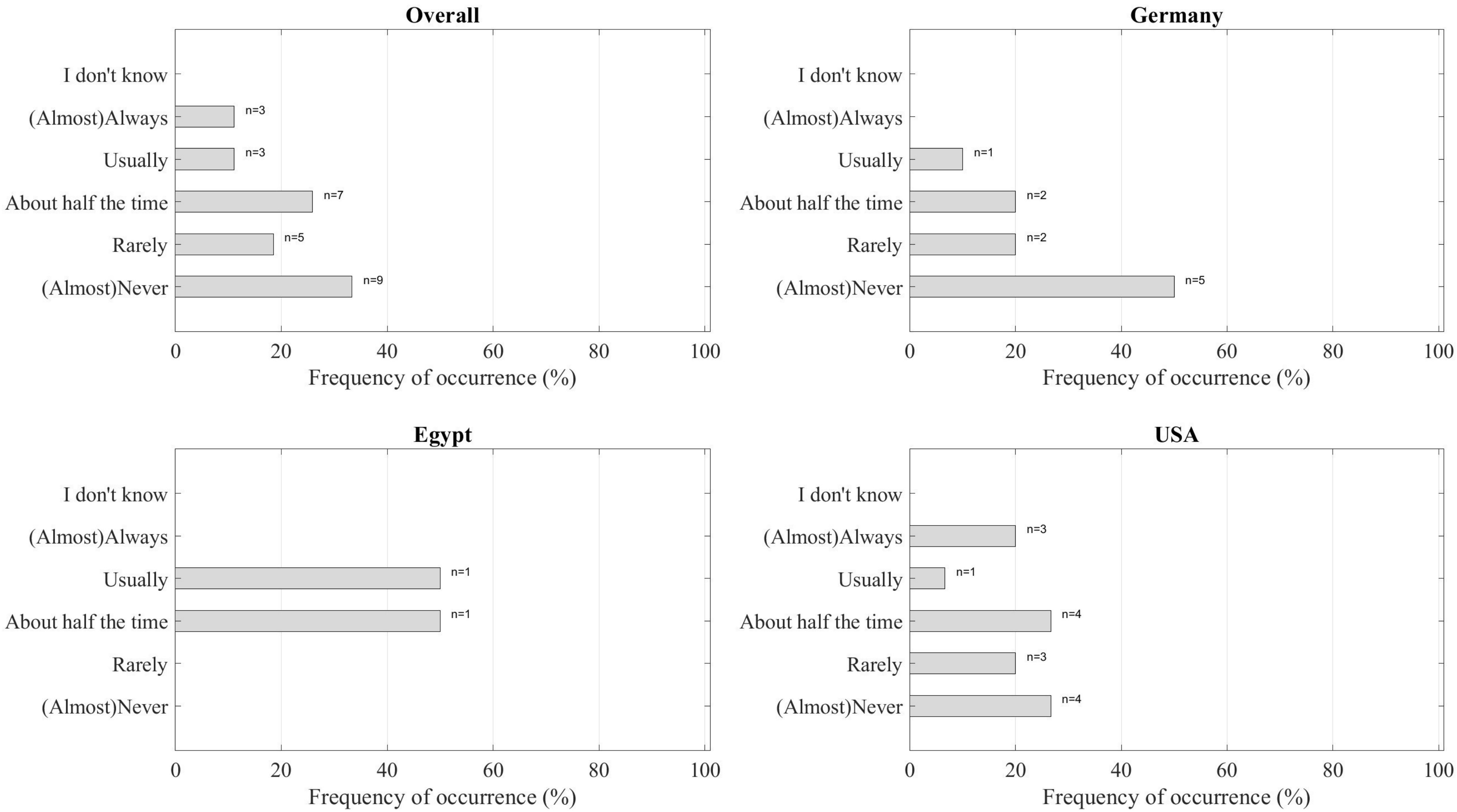

Figure A.16: Question 16: Knowing cause of hearing loss?

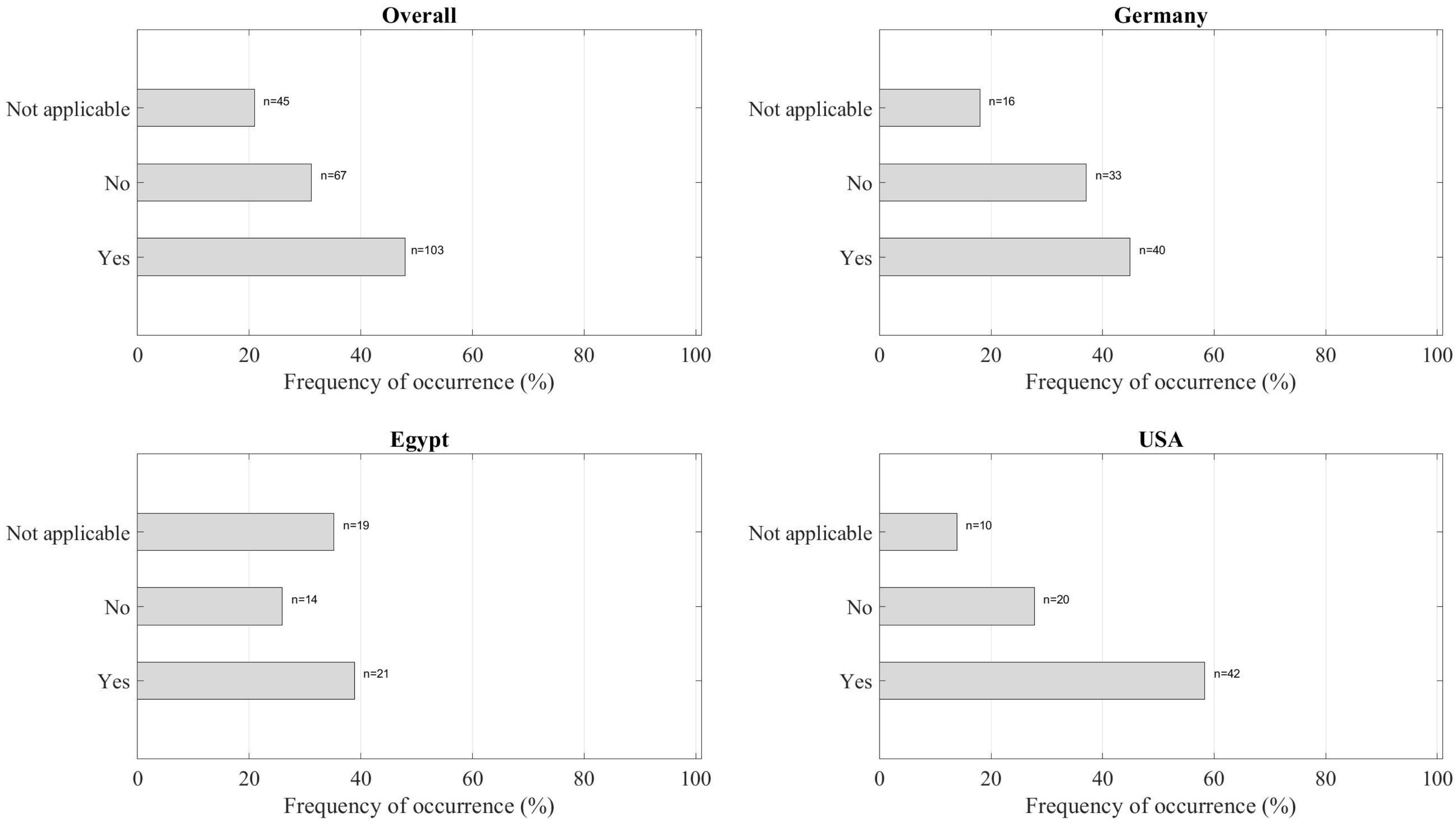

Figure A.17: Question 17: Main self-reported cause of hearing loss

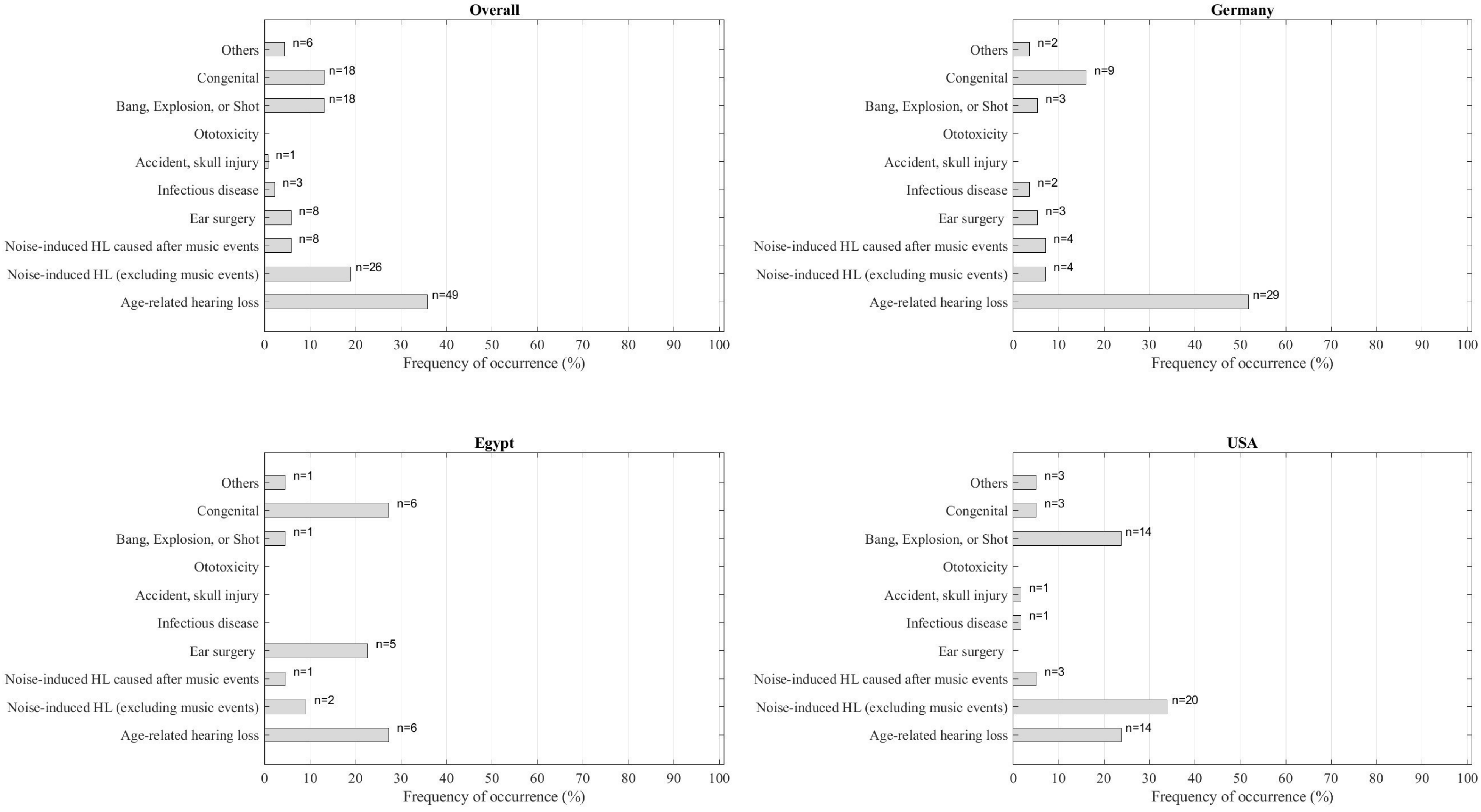

Figure A.18: Question 18: Sudden hearing loss?

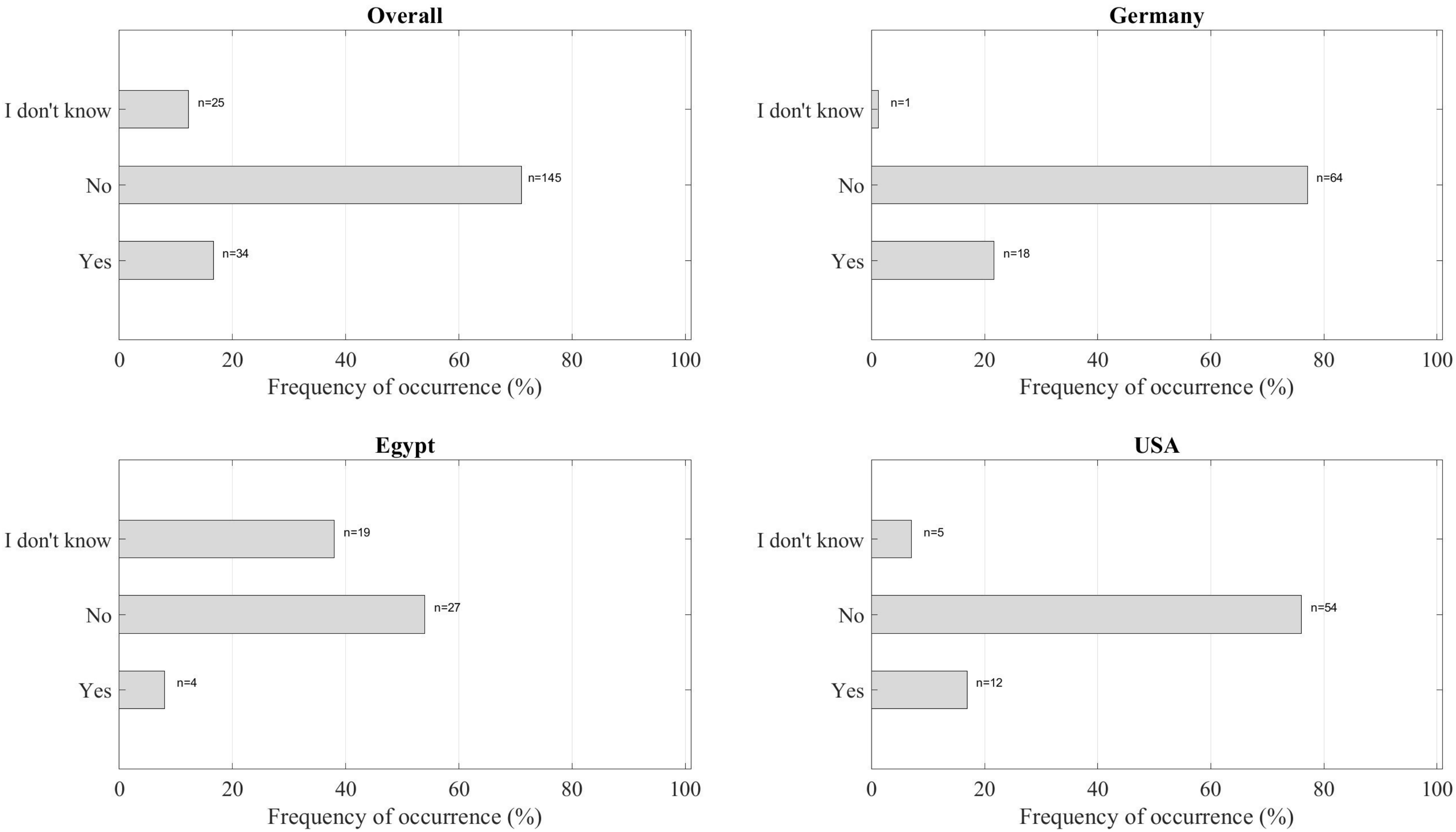

Figure A.19: Question 19: Surgical treatments of ear

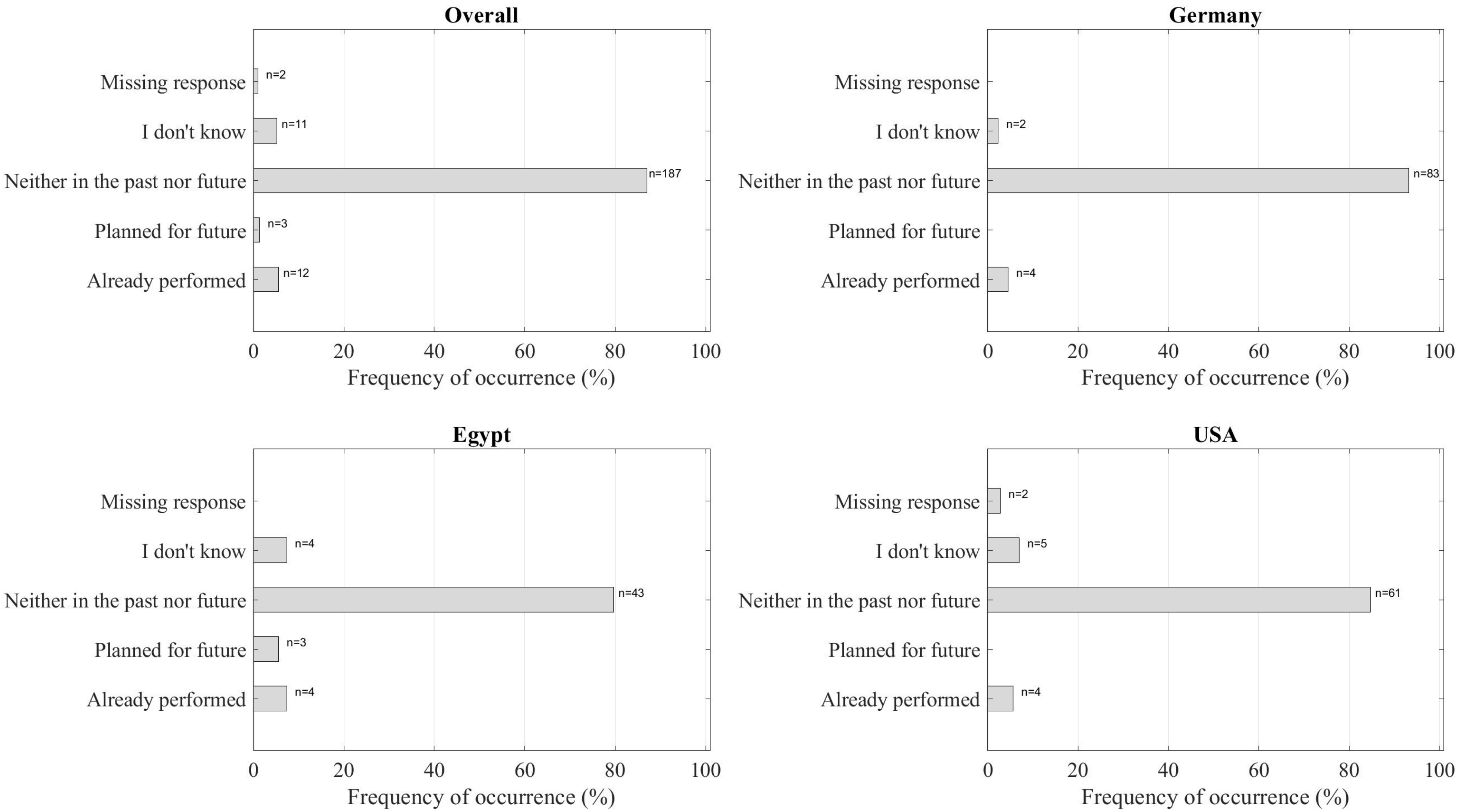

Figure A.20: Question 20: Middle ear infection

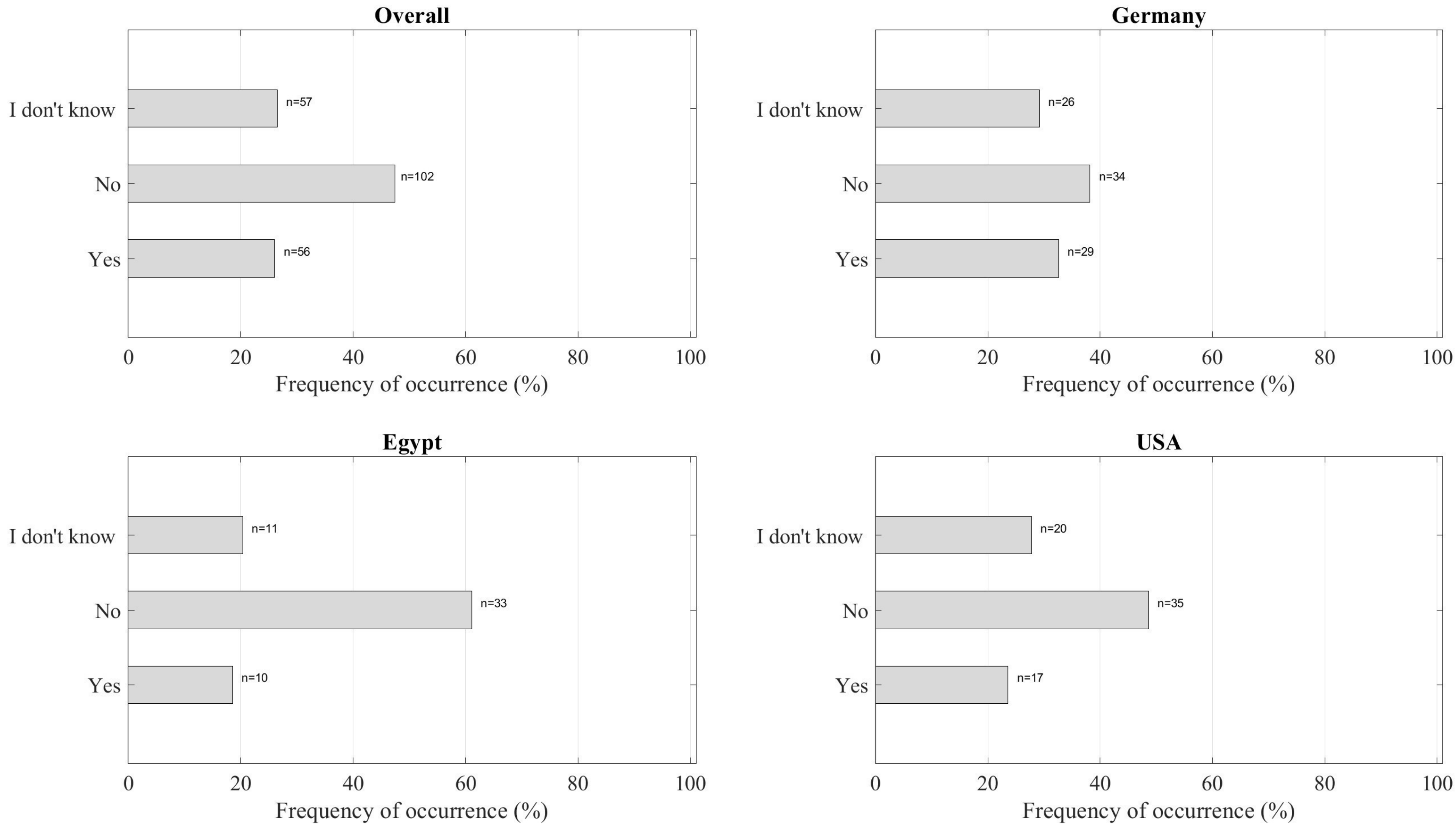

Figure A.21: Question 21: Having runny ear?

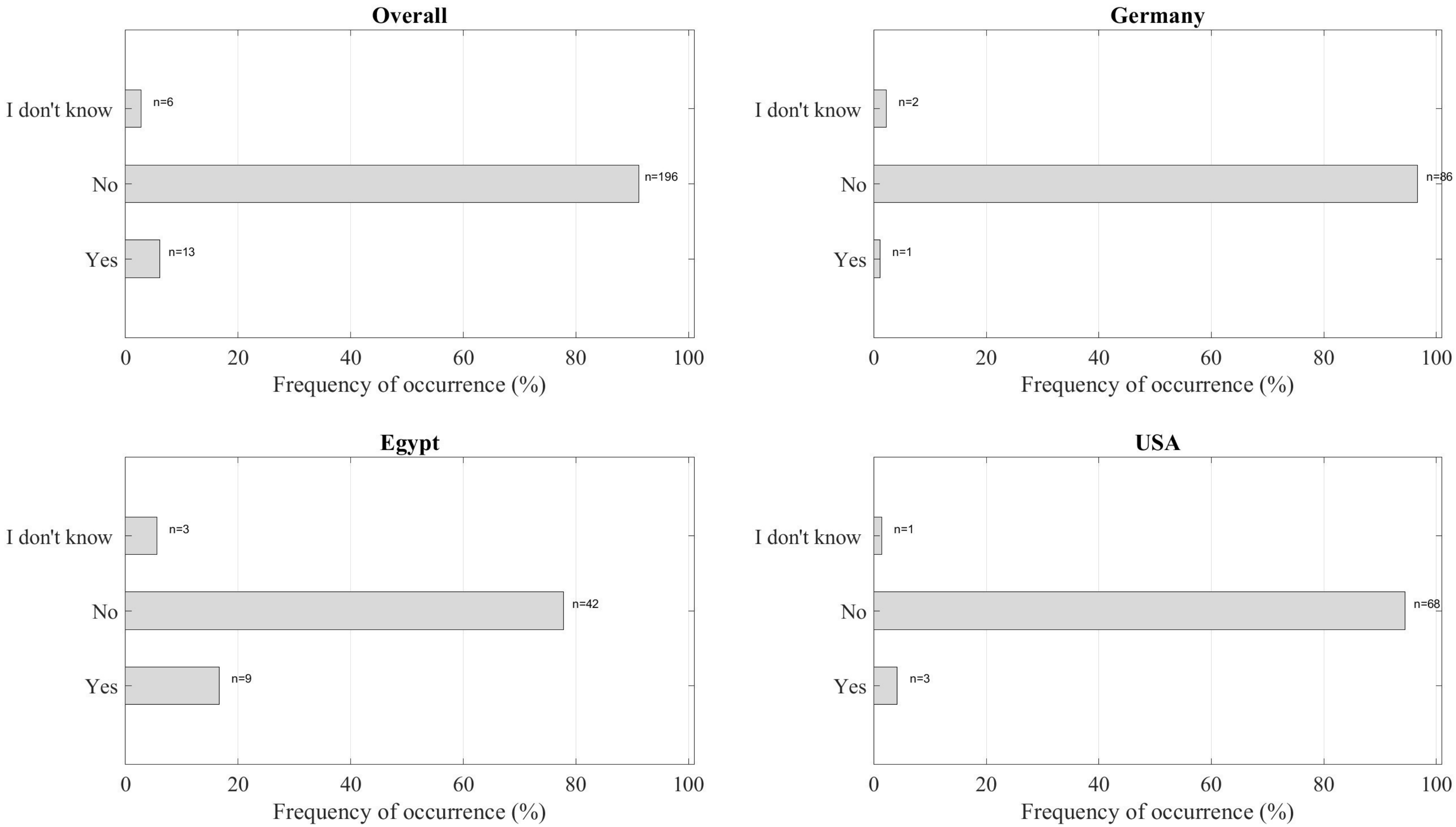

Figure A.22: Question 22: Last time hearing was tested?

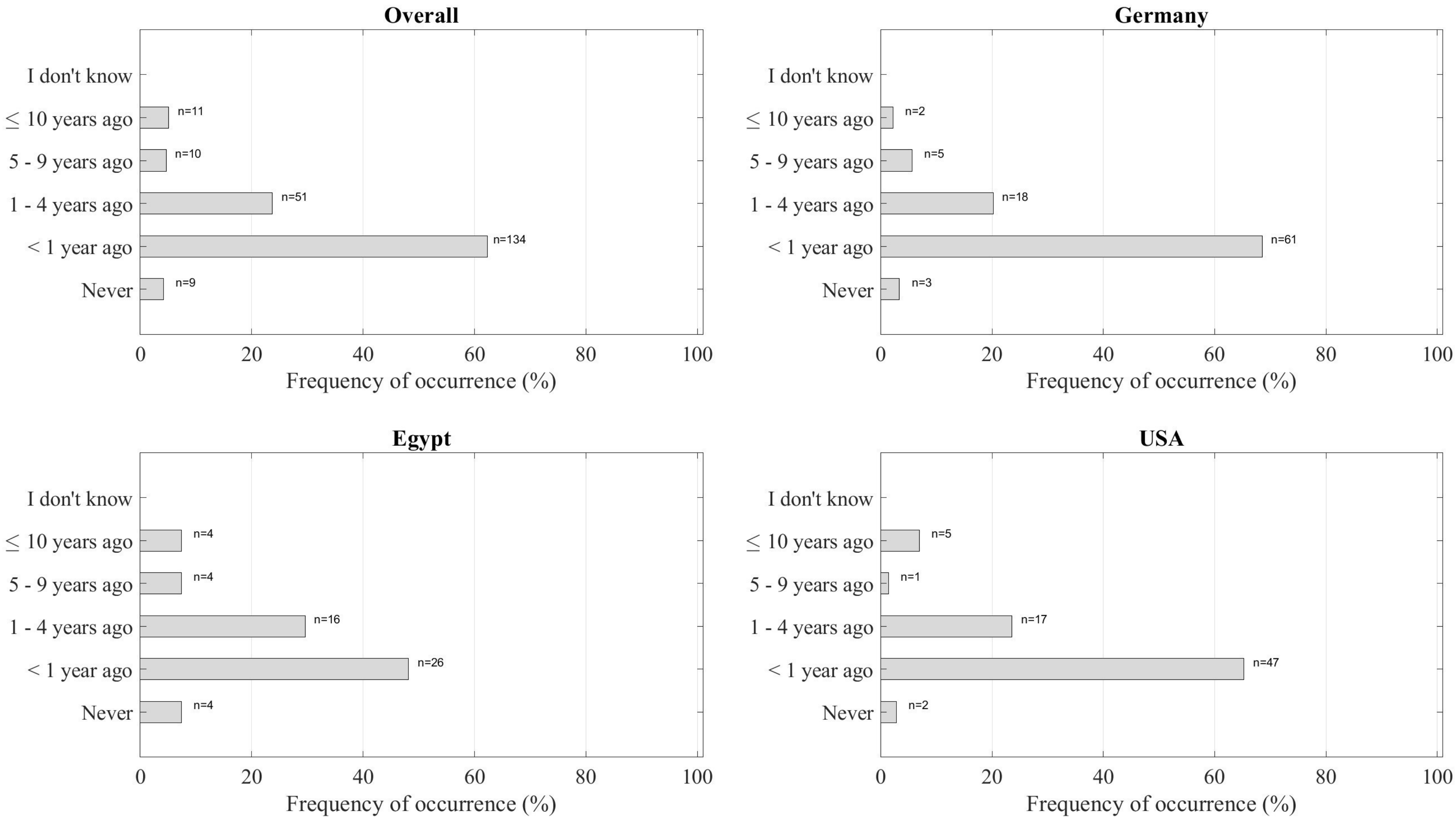

Figure A.23: Question 23: Family history of hearing problem

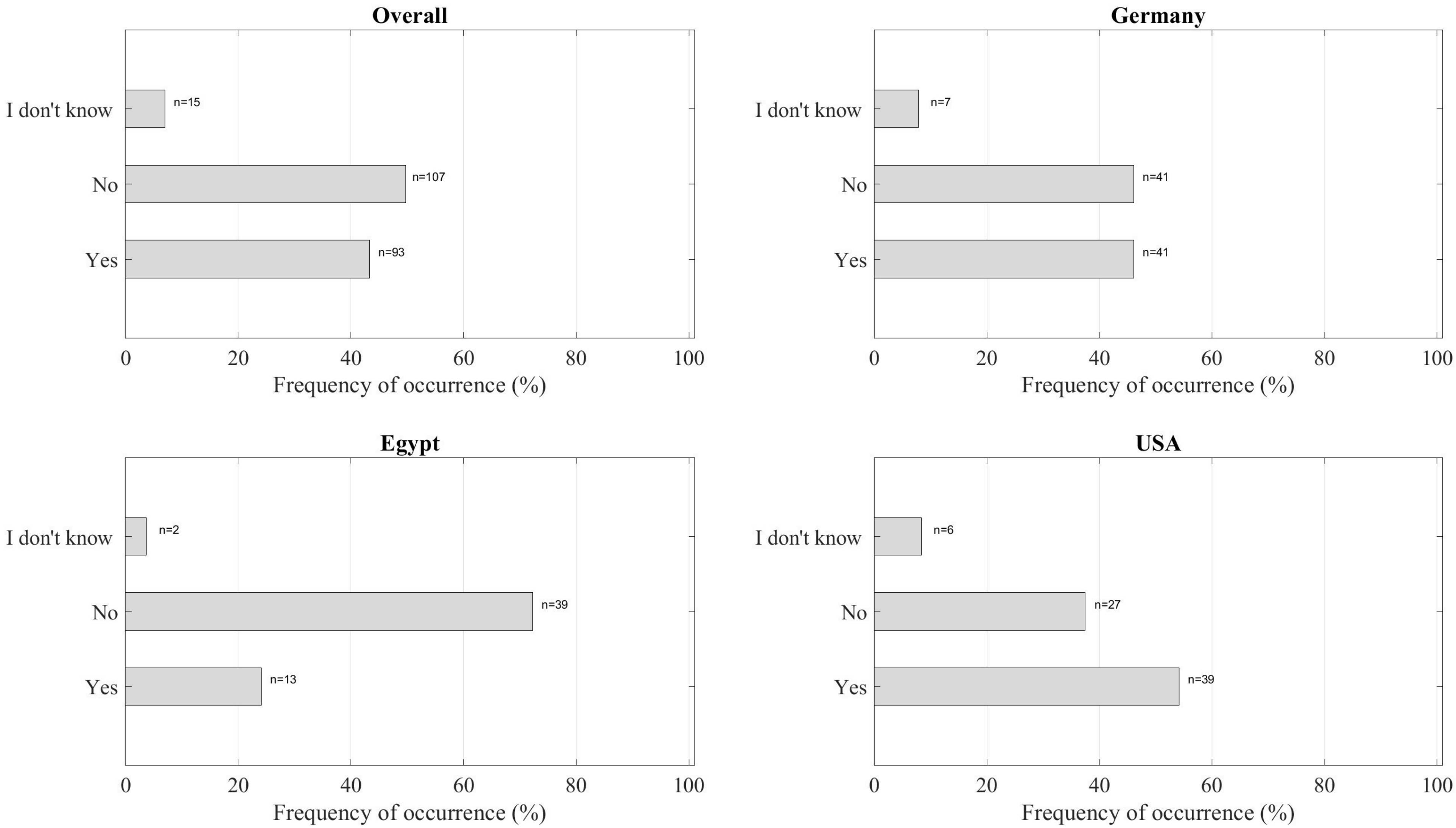

Figure A.24: Question 24: Hearing loss family history / Family side

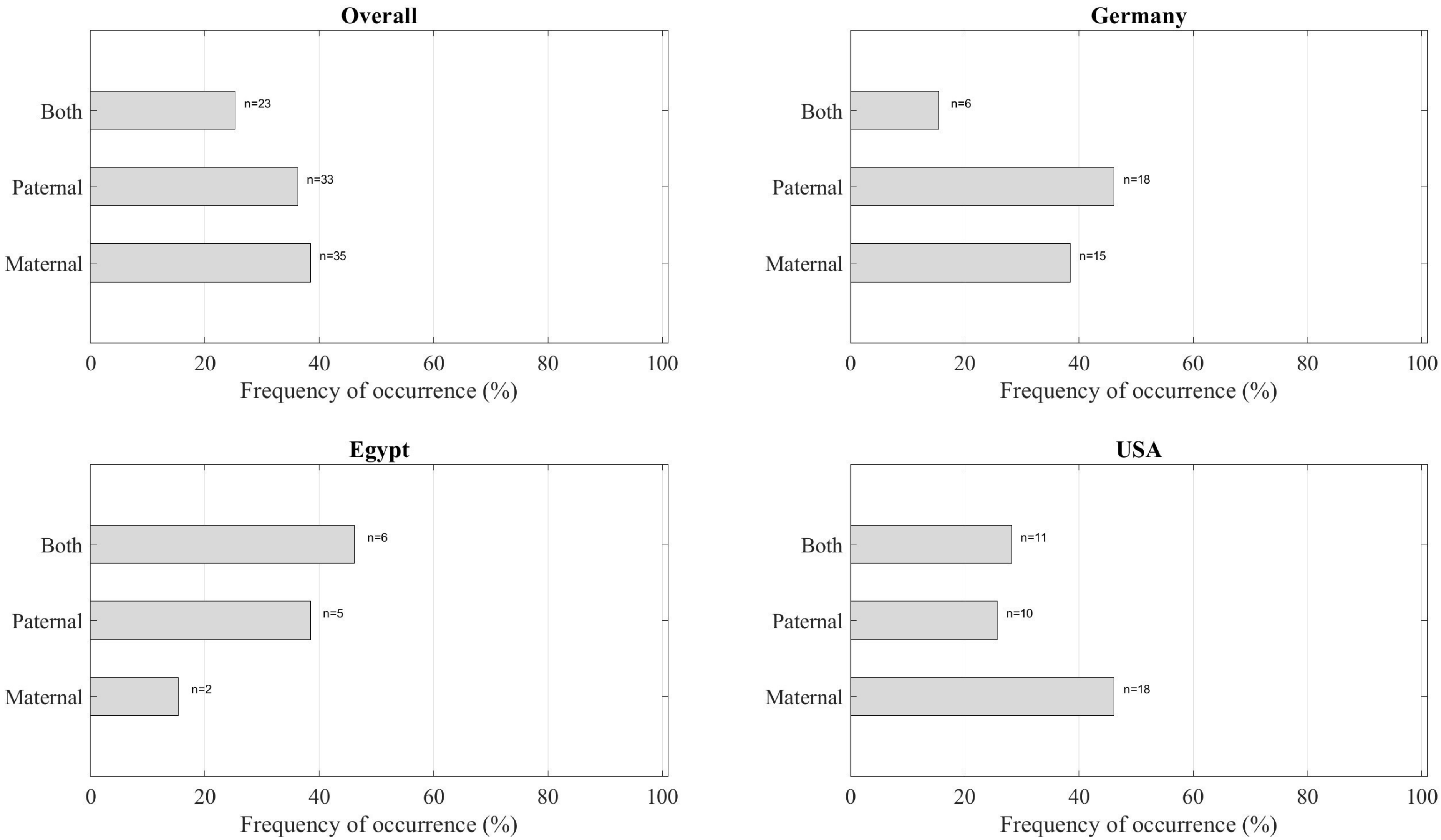

Figure A.25: Question 25: Hearing loss family history / Exact Relationship

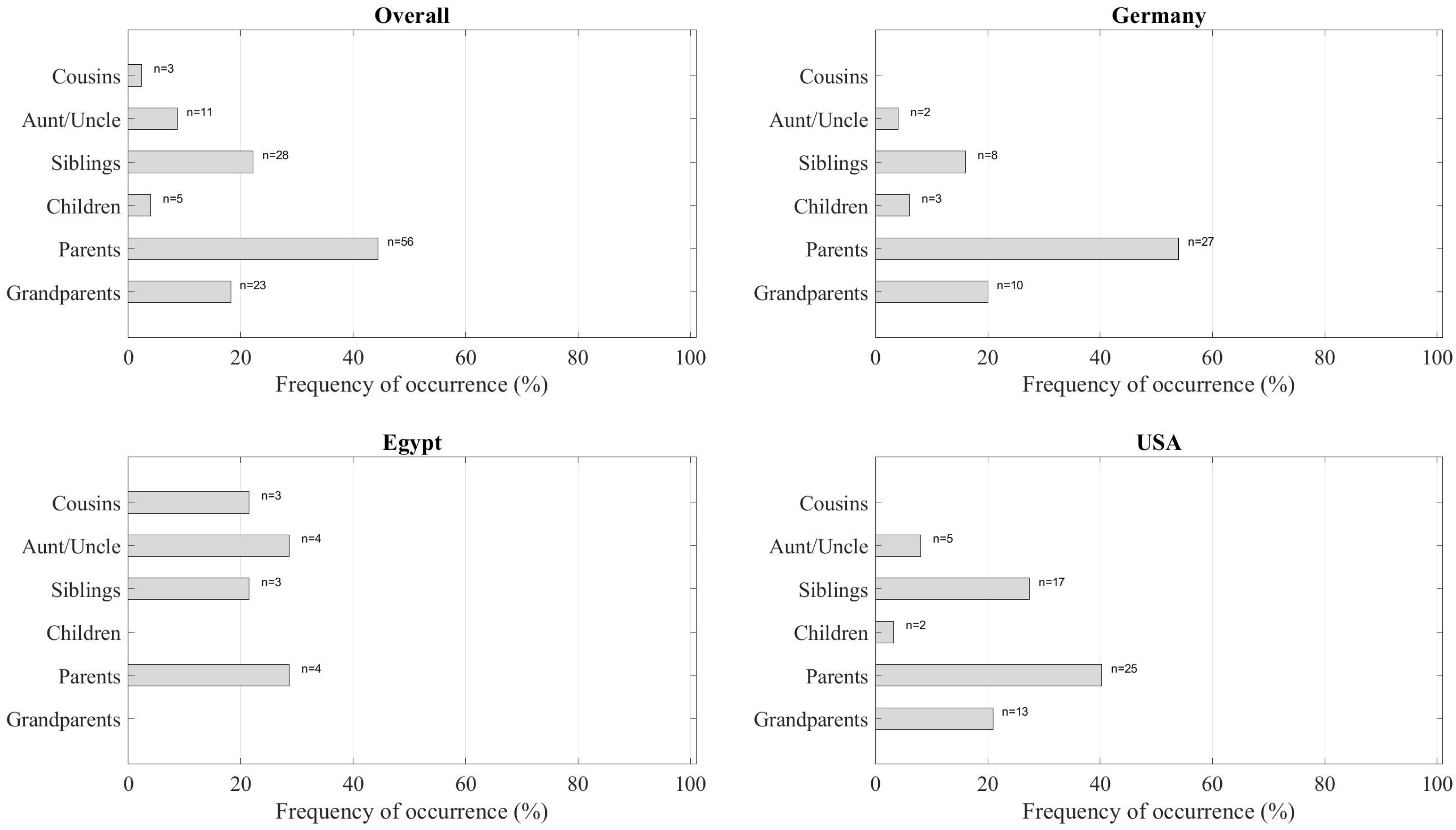

Figure A.26: Question 26: One ear hears better than the other?

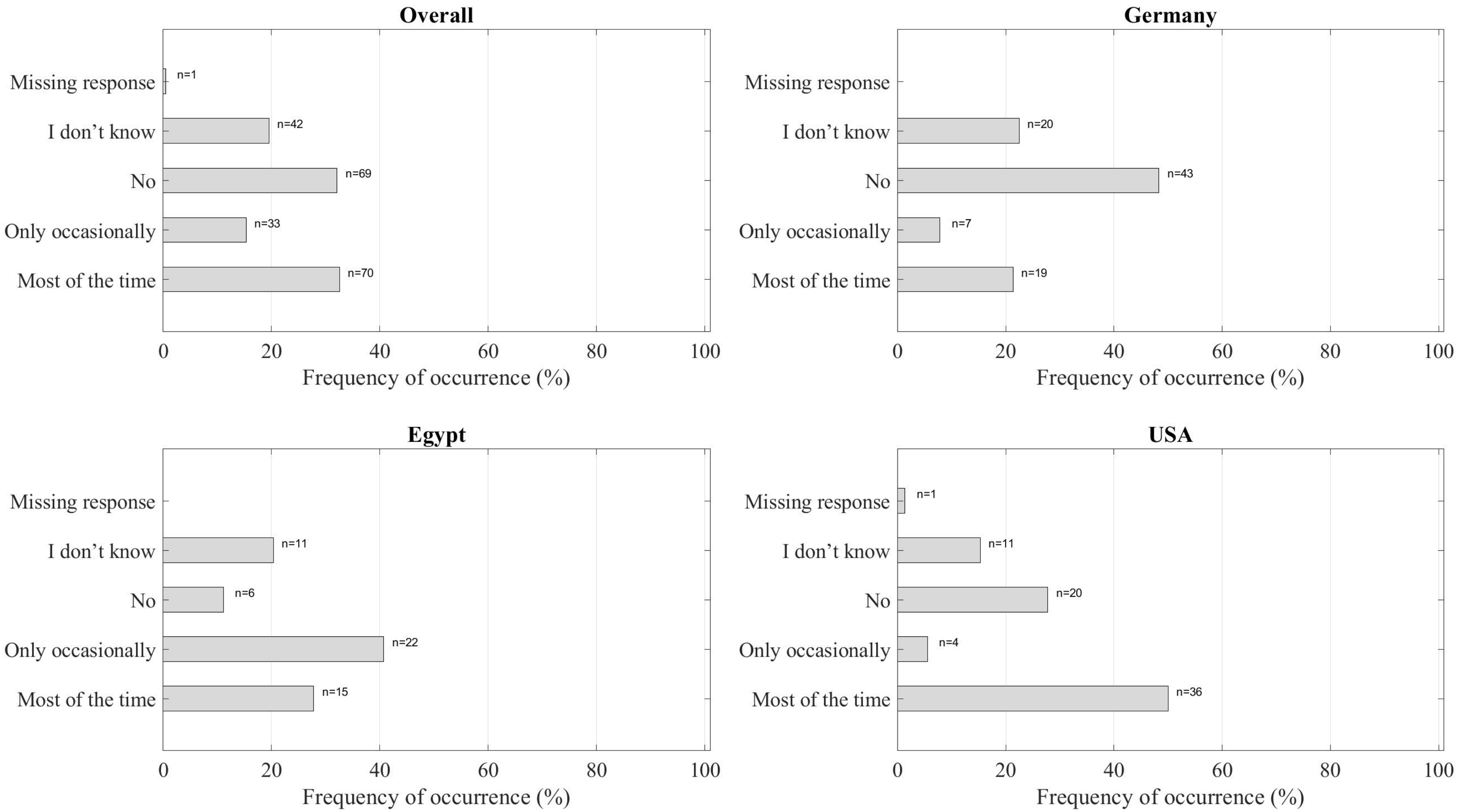

Figure A.27 (1): Question 27: Wearing hearing aid?

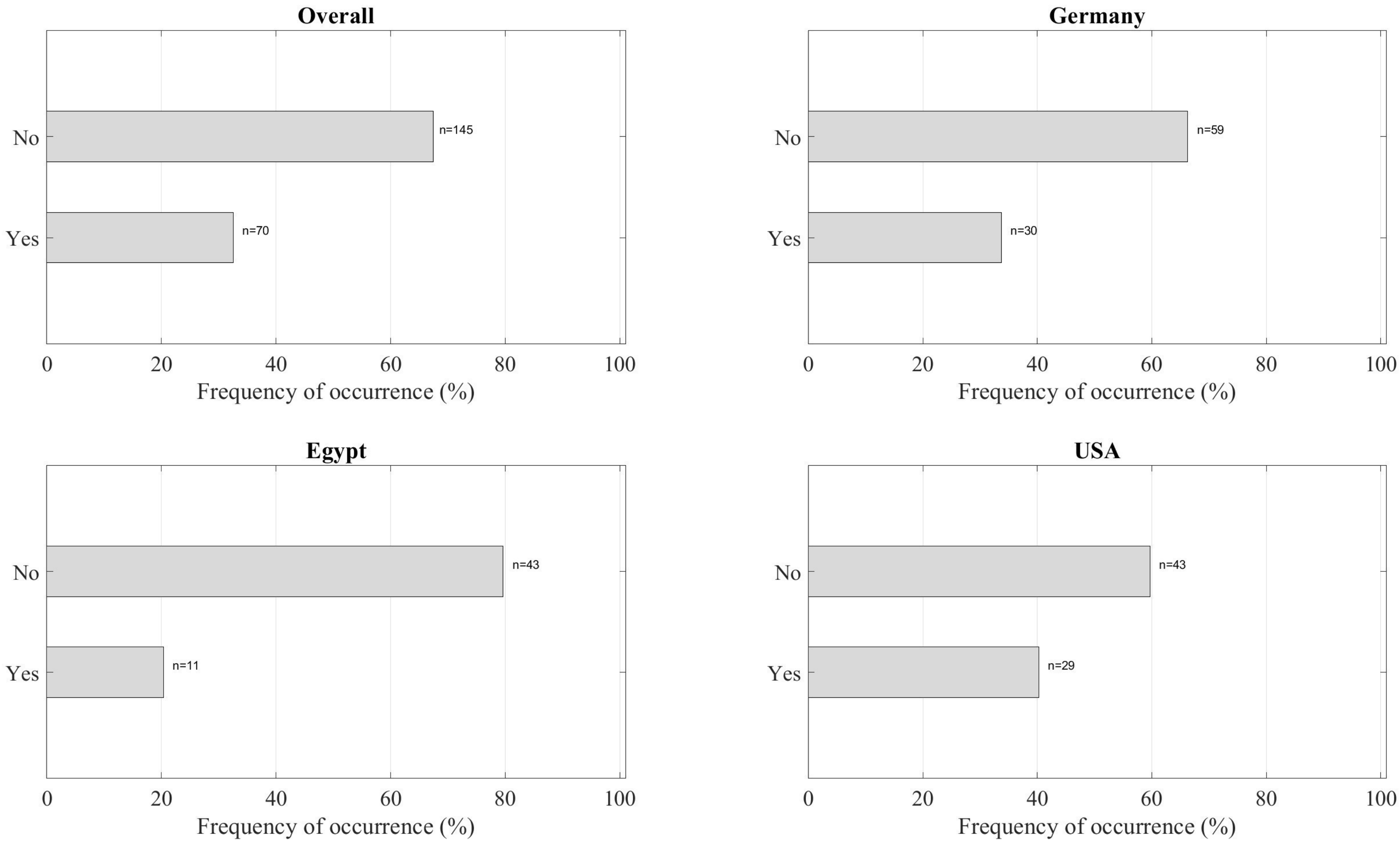

Figure A.27 (2): Question 27, In which ear?

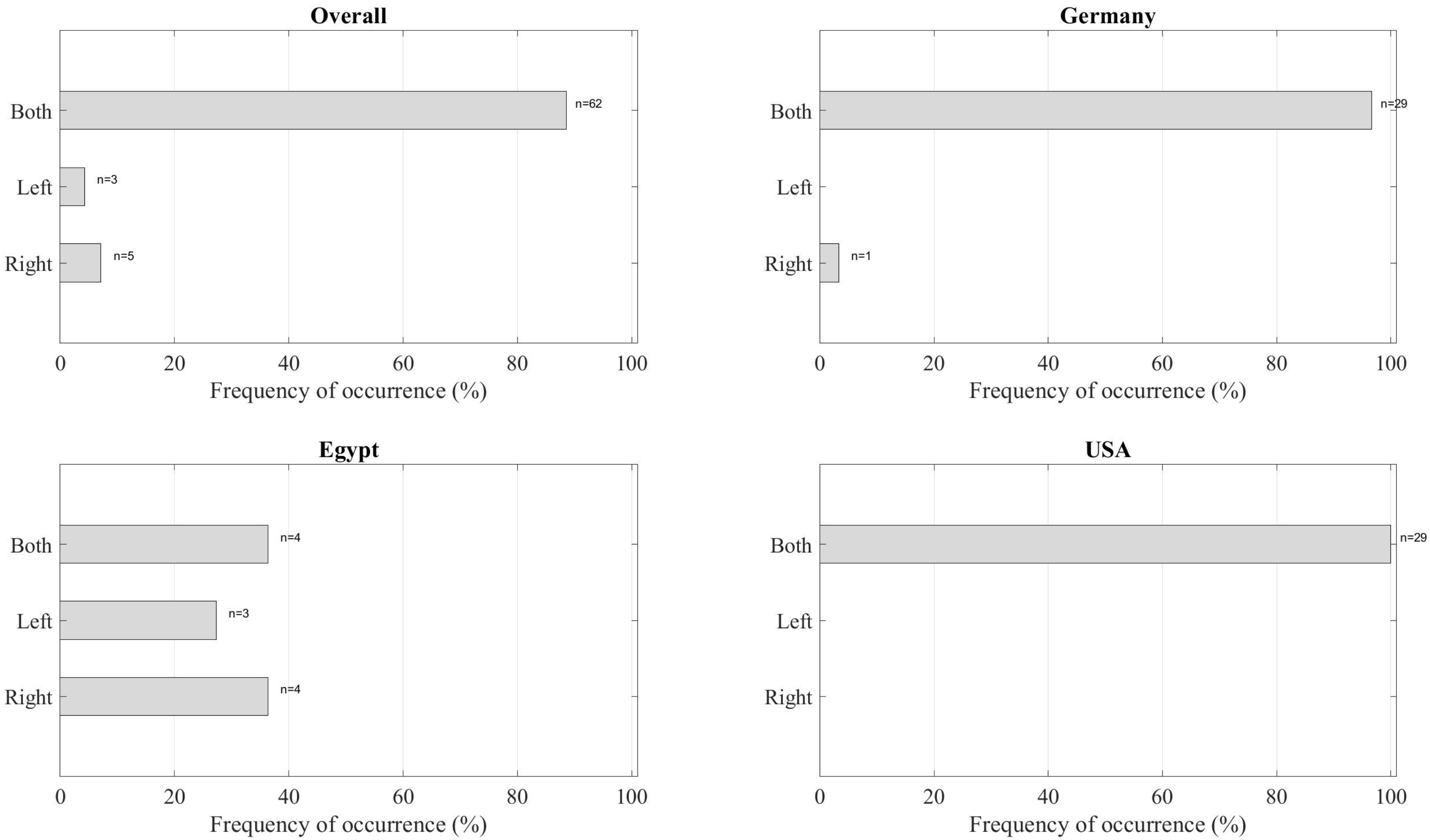

Figure A.28: Question 28: Which type?

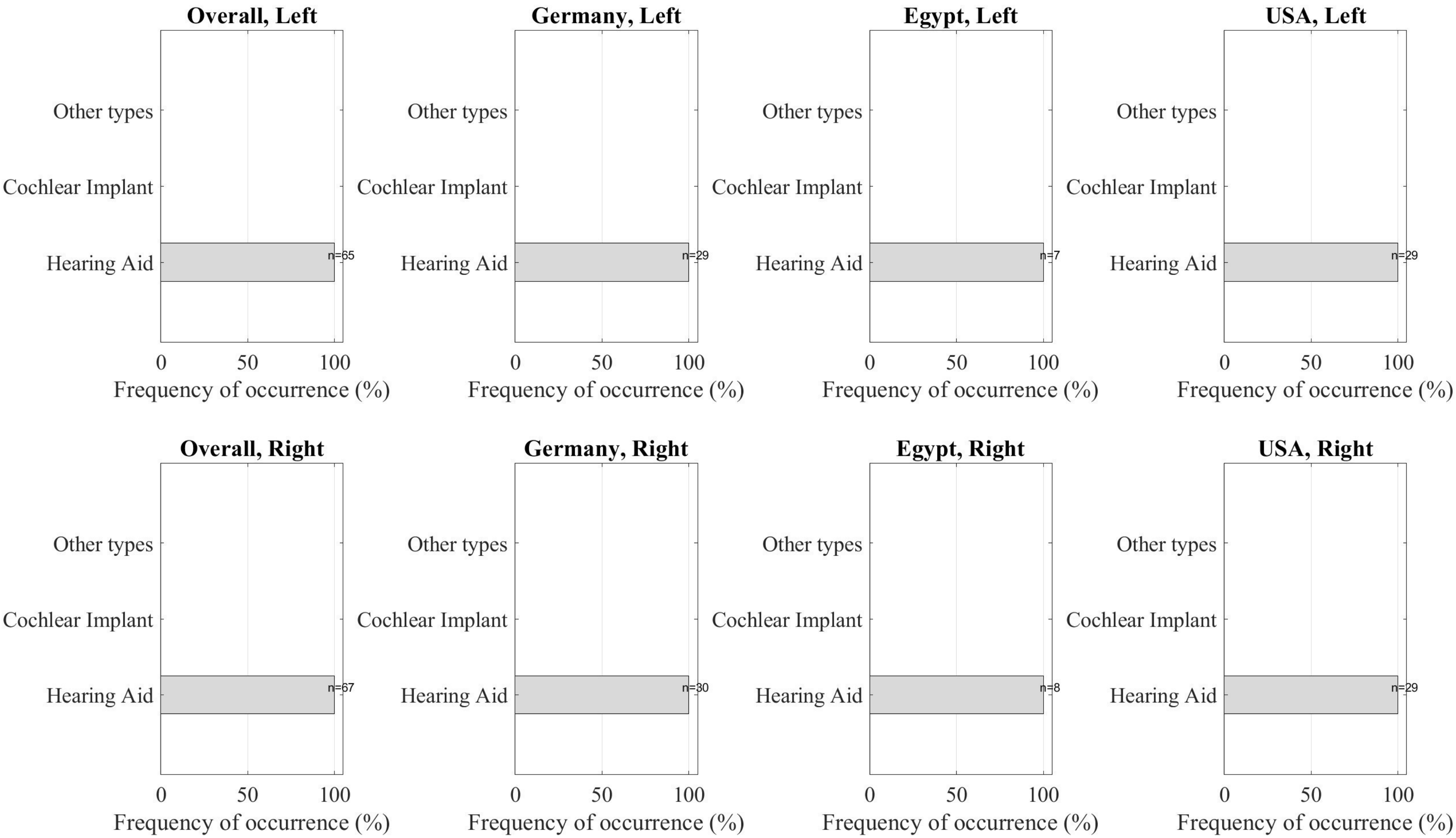

Figure A.29: Question 29: Used since ...

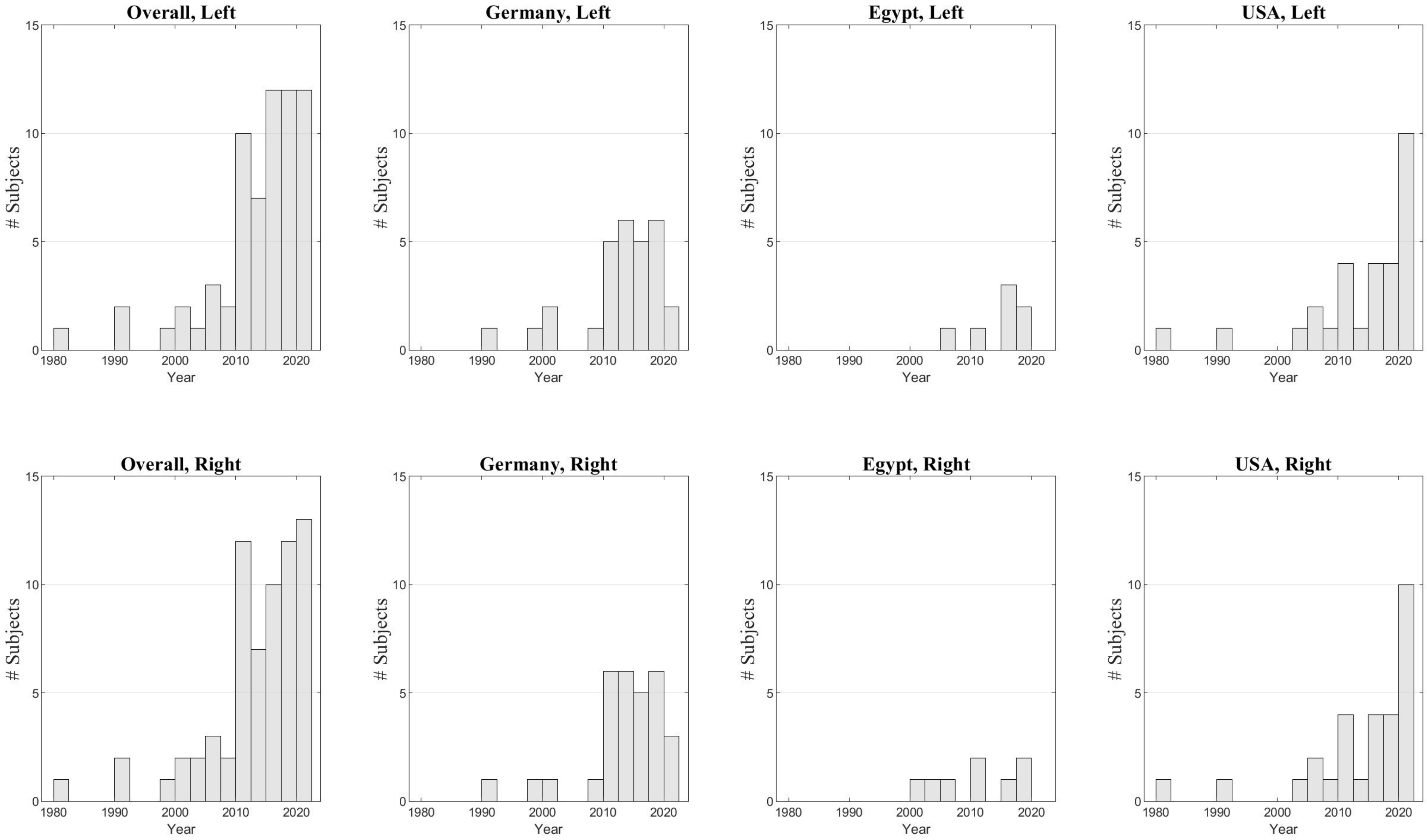

Figure A.30: Question 30: Usage time (hours/day)?

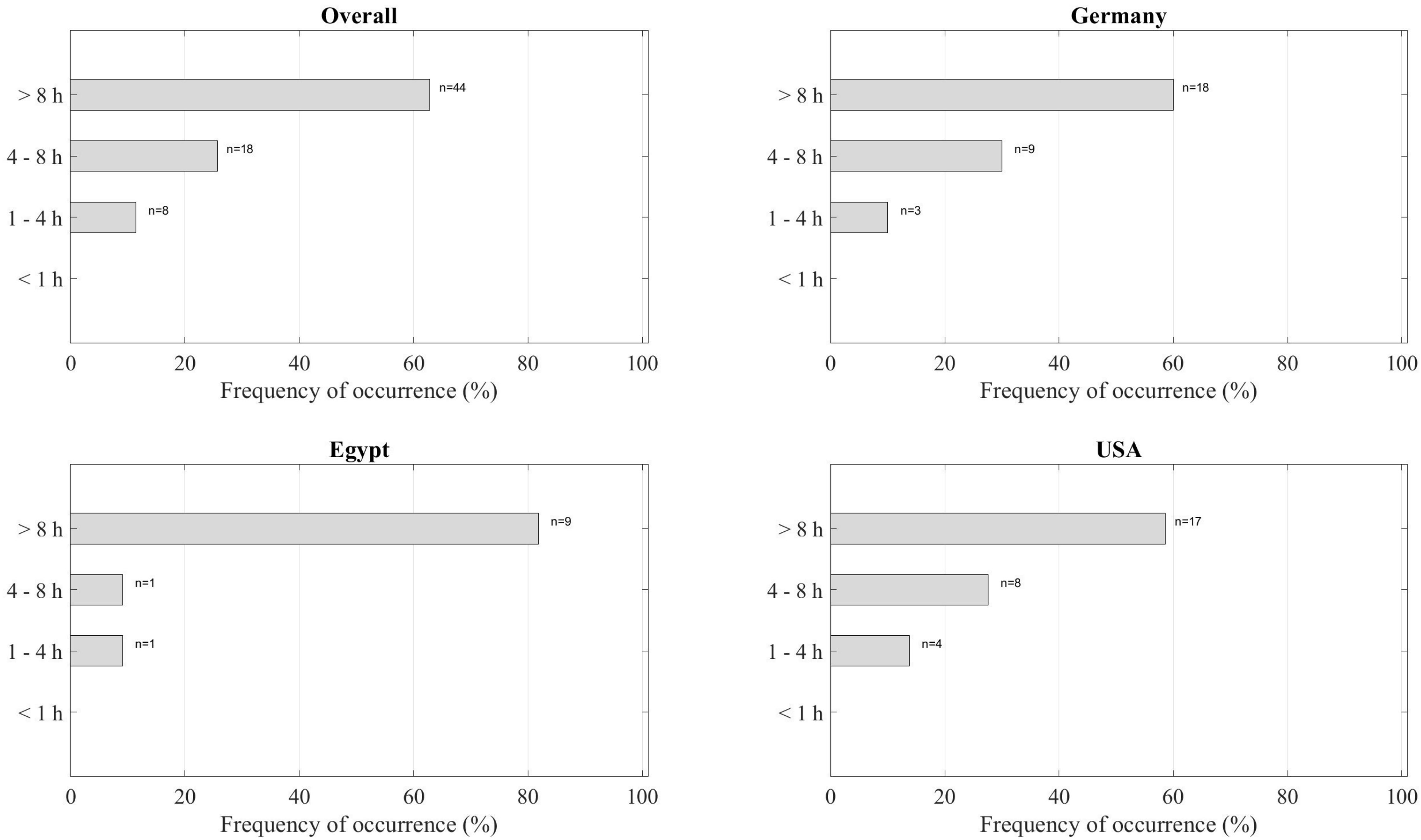

Figure P.1: PTA (better ear)

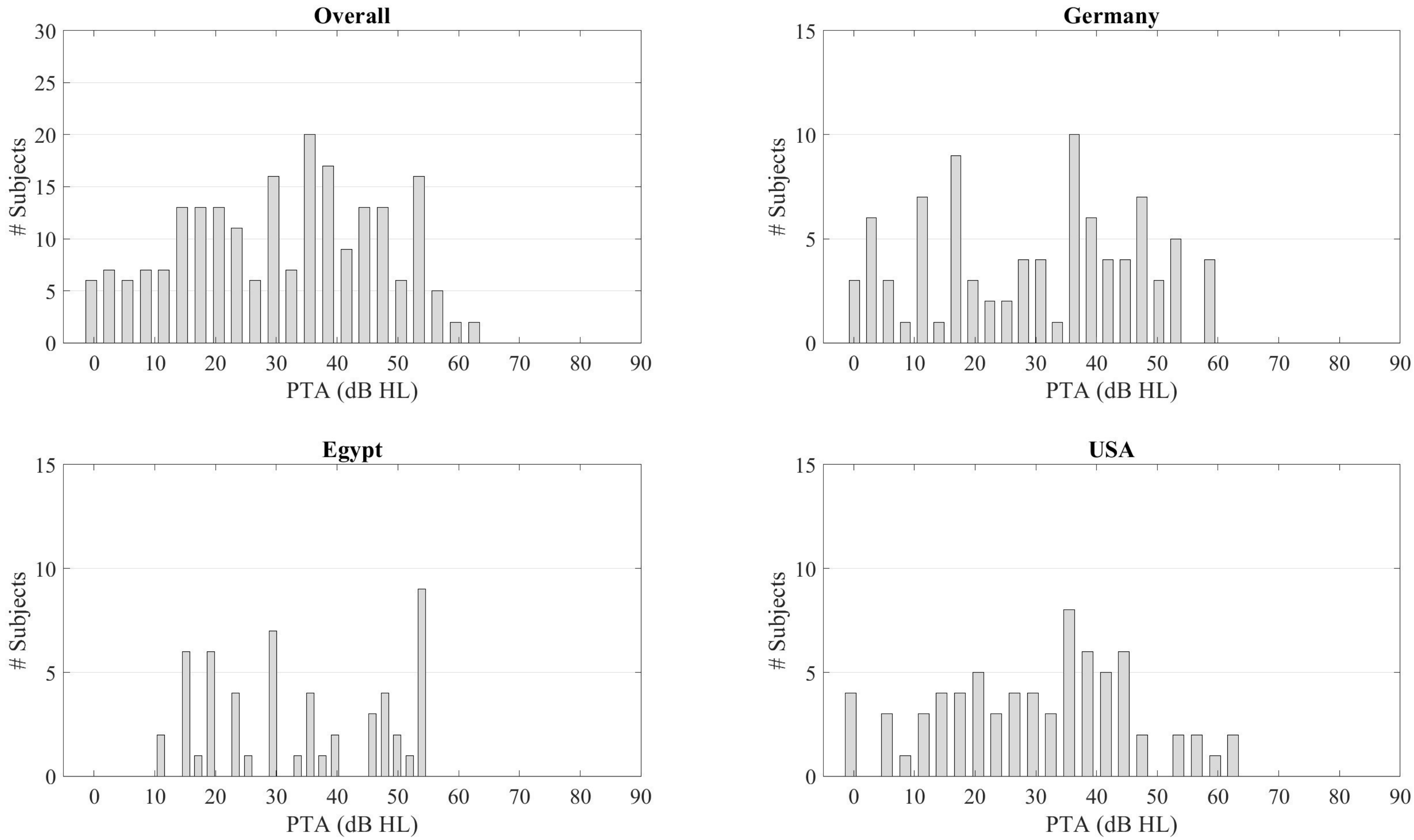

Figure P.2: PTA (worse ear)

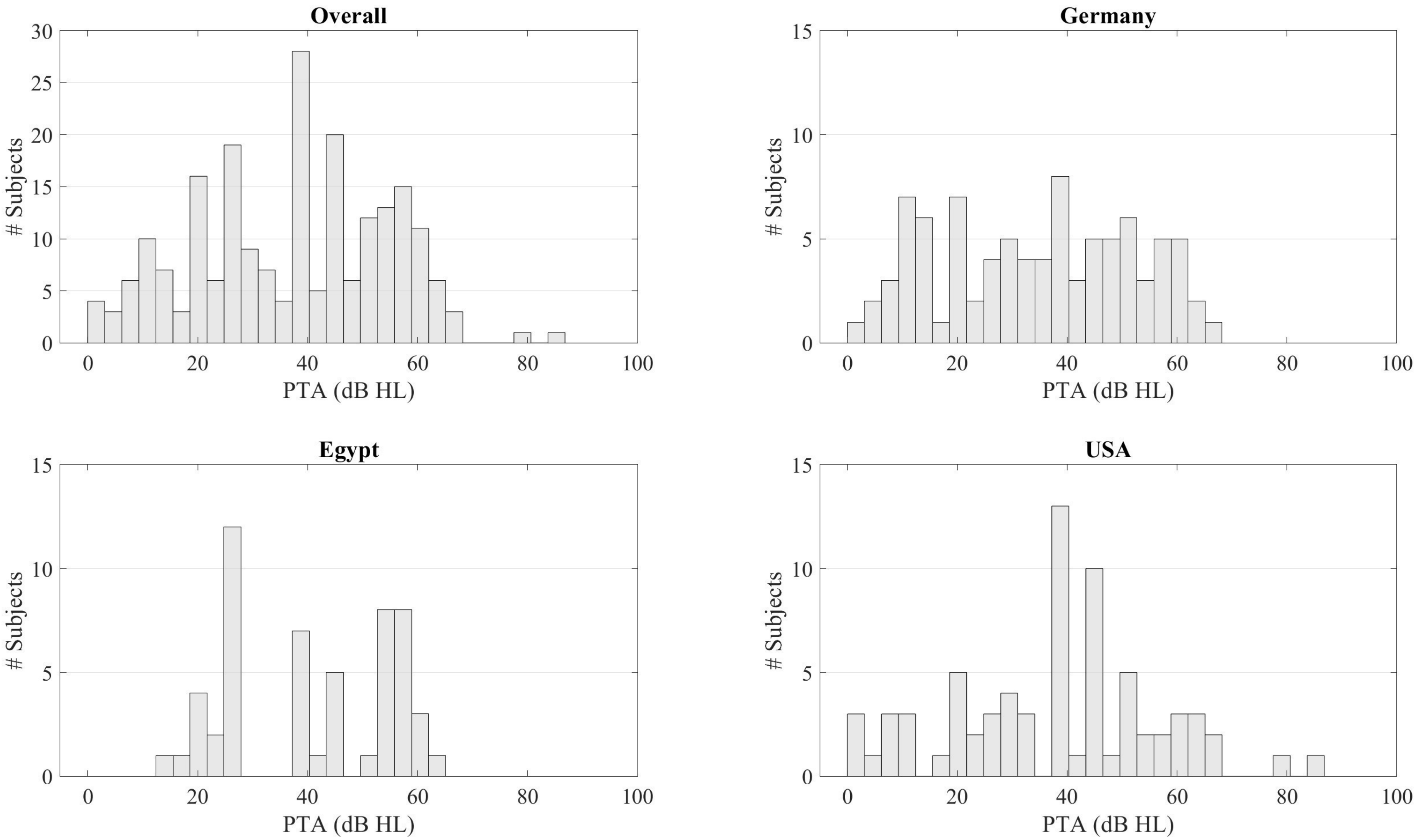

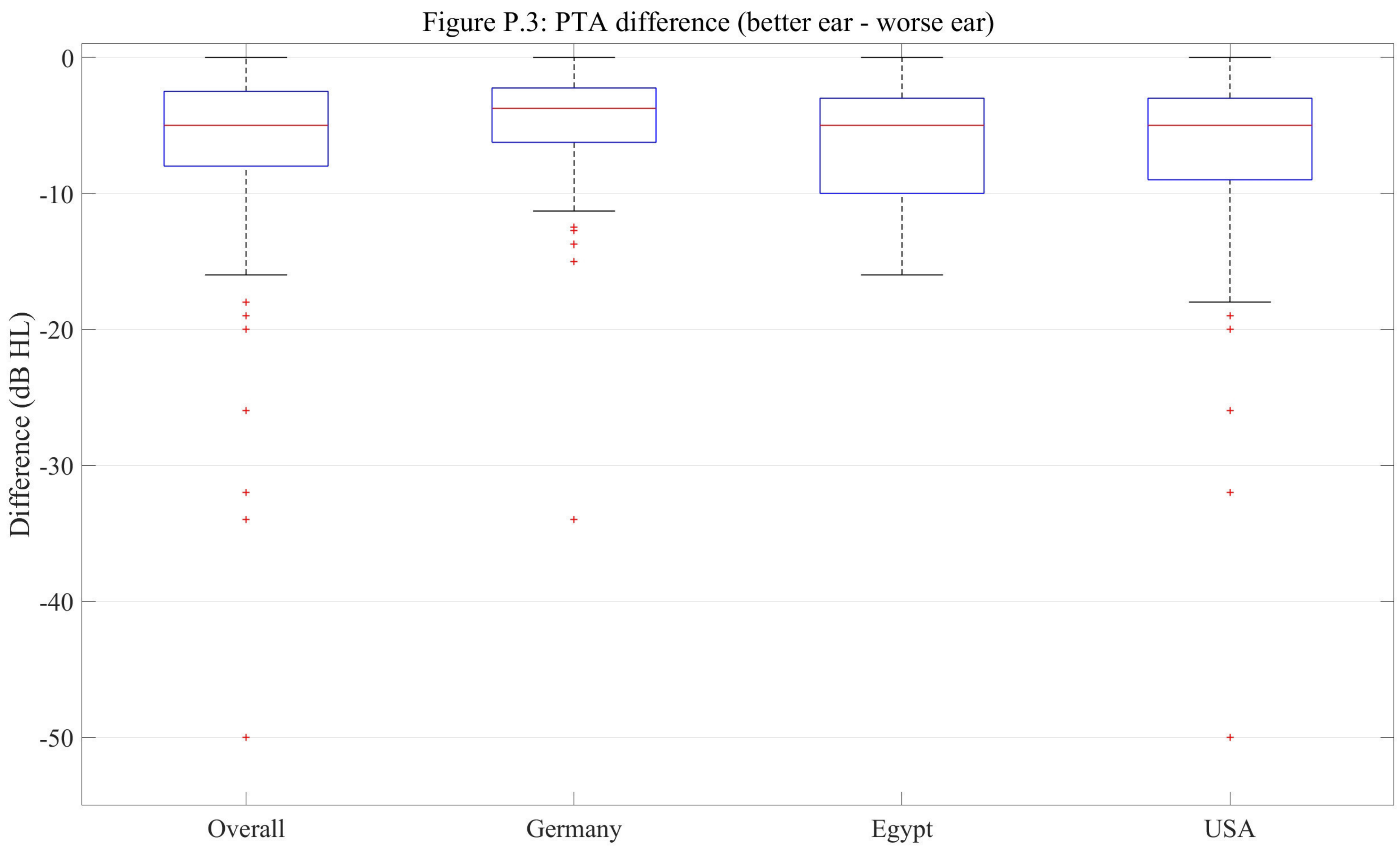

Figure P.3: PTA difference (better ear - worse ear)
Difference (dB HL)
0
-10
-20
-30
-40
-50
Overall
Germany
Egypt
USA

Figure P.4: PTA (Average)

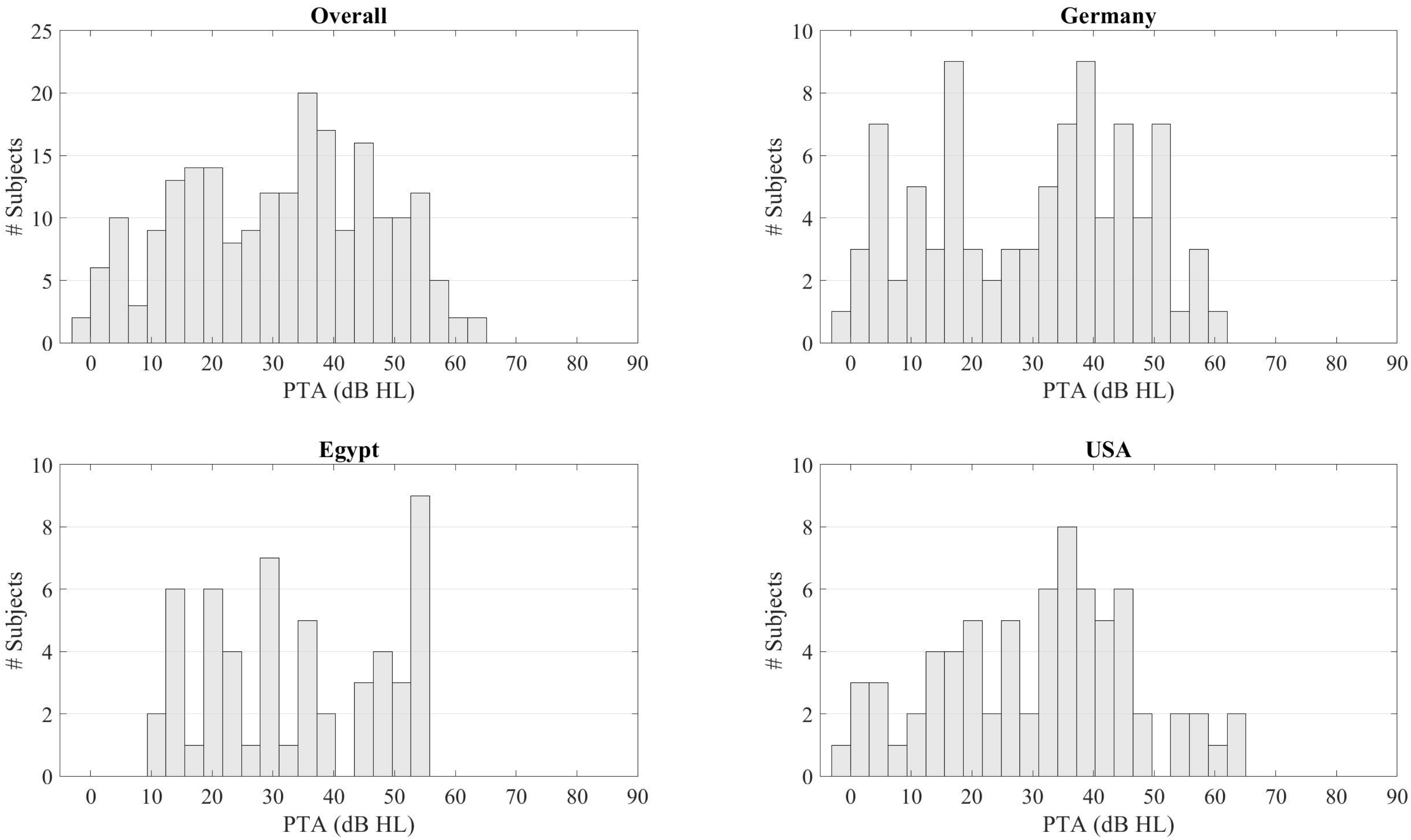

Figure P.5: Hearing status

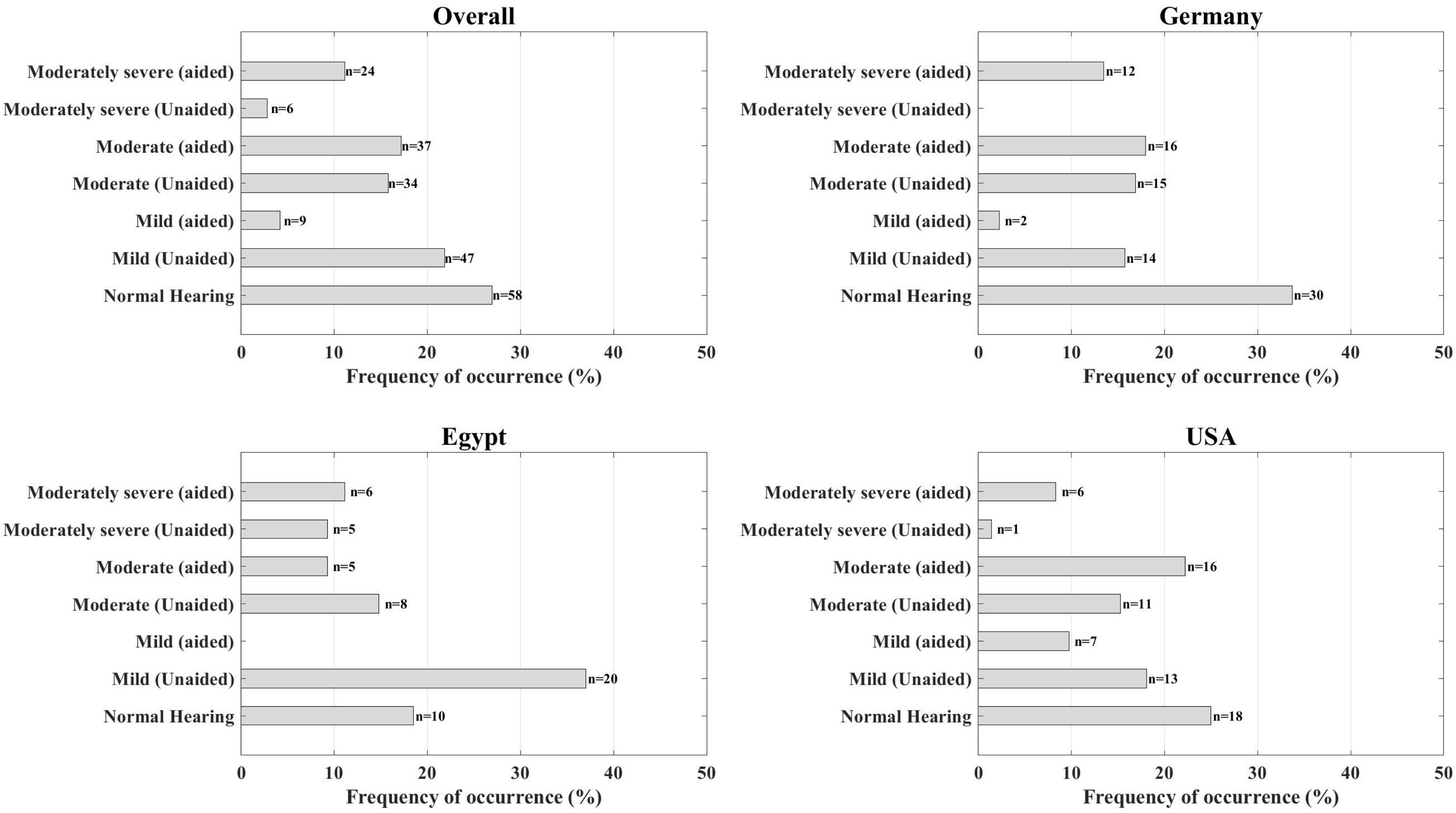

Table 1 The responses and descriptive statistics of the ICF-based items.

Item #	0 (%)	1 (%)	2 (%)	3 (%)	4 (%)	Non-gradable rate (%)	Min	Max	Median	Mean	Standard deviation
1	43.3	23.7	17.2	5.6	2.3	7.9	0	4	1	0.91	1.1
2	24.2	30.2	28.4	12.1	3.7	1.4	0	4	1	1.4	1.1
3	43.7	27.4	19.1	7	1.4	1.4	0	4	1	0.93	1
4	31.2	32.1	23.3	7.4	3.3	2.8	0	4	1	1.17	1.1
5	22.8	38.1	24.7	10.7	2.3	1.4	0	4	1	1.31	1
6	39.5	27.4	18.6	9.3	2.8	2.3	0	4	1	1.06	1.1
7	47.9	19.1	15.8	8.4	3.7	5.1	0	4	0	0.96	1.2
8	66.5	15.8	7.9	3.3	2.3	4.1	0	4	0	0.53	1
9	80.5	6	6	2.8	2.3	2.4	0	4	0	0.37	0.9
10	85.1	9.3	2.3	0.9	0.5	1.9	0	4	0	0.19	0.6
11	82.8	9.8	3.3	1.4	1.4	1.4	0	4	0	0.26	0.7
12	66	21.9	8.8	1.9	0	1.4	0	3	0	0.46	0.7
13	62.8	23.3	10.2	2.3	0.9	0.5	0	4	0	0.55	0.8
14	56.7	21.9	16.7	3.7	0	0.9	0	3	0	0.67	0.9
15	61.4	23.3	11.6	1.9	0	1.9	0	3	0	0.53	0.8
16	76.3	14.4	4.2	0.5	0	4.7	0	3	0	0.25	0.6
17	75.3	13	6.5	0.5	0	4.7	0	3	0	0.29	0.6
18	23.7	29.3	17.7	17.2	10.7	1.4	0	4	1	1.61	1.3
19	54	25.6	9.3	6	3.3	1.9	0	4	0	0.77	1.1
20	56.3	30.7	6.5	1.9	3.3	1.4	0	4	0	0.63	0.9
21	40.5	24.2	19.1	9.3	1.4	5.6	0	4	1	1.01	1.1
22	40.5	27.4	15.8	8.4	0.9	7	0	4	1	0.95	1
23	36.3	30.7	14.4	9.8	1.4	7.4	0	4	1	1.02	1.1
24	35.8	36.7	15.8	8.4	2.3	0.9	0	4	1	1.04	1
25	56.3	21.9	13	6	1.9	0.9	0	4	0	0.74	1
26	70.7	10.7	7.9	7	0.9	2.8	0	4	0	0.53	1
27	45.6	22.8	7.9	6	1.4	16.3	0	4	0	0.74	1
28	43.3	31.2	13	8.4	2.3	1.9	0	4	1	0.93	1.1
29	64.7	14	10.7	3.3	2.3	5.1	0	4	0	0.57	1
30	48.8	24.2	9.8	6.5	0.9	9.8	0	4	0	0.74	1
31	44.7	26	13	5.1	0.9	10.3	0	4	1	0.79	1
32	51.6	21.4	7.9	6.5	1.9	10.7	0	4	0	0.72	1
33	59.1	18.6	9.3	7.4	0.9	4.7	0	4	0	0.66	1
34	22.8	34.4	23.7	12.6	2.8	3.8	0	4	1	1.36	1.1
35	36.3	28.4	22.8	8.8	2.3	1.4	0	4	1	1.11	1.1
36	55.3	24.7	13.5	4.2	0.9	1.5	0	4	0	0.69	0.9
37	16.7	34.9	24.7	16.3	6.5	0.9	0	4	1	1.61	1.1
38	45.6	27.4	18.6	7	0.5	1	0	4	1	0.88	1
39	22.8	35.8	21.9	14	3.3	2.4	0	4	1	1.38	1.1
40	19.1	38.6	25.6	11.2	3.3	2.3	0	4	1	1.4	1

41	5.1	94.9									
42	1.9	0.5	0.9	0	0	96.8	0	2	0	0.71	1
43	1.4	0.5	1.4	0.9	0	95.8	0	3	2	1.44	1.2
44	1.4	0.9	0.5	0.9	0	96.3	0	3	1	1.25	1.3
45	1.4	0.9	0.9	0.9	0	95.8	0	3	1	1.33	1.2
46	0.5	1.9	1.4	0.5	0	95.8	0	3	1	1.44	0.9
47	2.3	0	0.9	0.5	0	96.3	0	3	0	0.88	1.2
48	0.9	1.4	0.9	0.5	0	96.3	0	3	1	1.25	1
49	36.3	35.3	18.6	7.9	0.5	1.4	0	4	1	1	1
50	62.8	16.7	9.3	5.1	0.5	5.6	0	4	0	0.56	0.9
51	60.5	19.1	13	2.3	0.5	4.7	0	4	0	0.57	0.9
52	54.9	19.5	6.5	8.4	6.5	4.2	0	4	0	0.87	1.3
53	61.4	11.2	13	6	1.4	7	0	4	0	0.66	1
54	64.7	15.8	11.2	5.1	1.9	1.4	0	4	0	0.62	1
55	51.6	21.9	10.7	8.8	1.4	5.6	0	4	0	0.8	1.1
56	48.4	25.1	12.1	7	2.8	4.7	0	4	0	0.85	1.1
57	46	28.8	15.8	4.2	2.3	2.9	0	4	1	0.85	1
58	69.8	16.3	7.4	4.2	0.9	1.4	0	4	0	0.48	0.9
59	57.7	18.1	12.6	3.7	0.9	7.1	0	4	0	0.63	0.9
60	64.2	14	7.9	3.3	1.4	9.4	0	4	0	0.5	0.9
61	57.7	14.9	7.4	4.7	1.9	13.5	0	4	0	0.59	1
62	35.3	18.6	9.8	3.7	3.7	28.8	0	4	1	0.9	1.1
63	51.6	16.3	11.2	3.3	0.9	16.7	0	4	0	0.63	0.9
64	52.6	18.1	9.3	4.7	0.5	14.9	0	4	0	0.62	0.9
65	47	21.4	12.6	7	0	12	0	3	0	0.77	1
66	41.4	24.7	14.4	7.4	0.5	11.6	0	4	1	0.88	1
67	60.5	21.9	8.4	6.5	1.4	1.4	0	4	0	0.65	1
68	50.9	21.5	12.1	9.3	3.3	2.8	0	4	0	0.89	1.2
69	34.9	26.5	18.6	13.5	6	0.5	0	4	1	1.29	1.2
70	42.3	24.7	19.5	7.9	3.3	2.4	0	4	1	1.03	1.1
71	15.8	34.4	23.7	17.7	7.4	0.9	0	4	1	1.66	1.2
72	71.2	16.7	6.5	3.7	1.4	0.5	0	4	0	0.47	0.9
73	48.4	26.5	13	6	1.4	4.7	0	4	0	0.8	1
74	43.3	31.6	16.3	5.1	0.5	3.3	0	4	1	0.84	0.9
75	7.9	18.6	19.1	21.9	19.1	13.6	0	4	2	2.3	1.3
76	4.2	16.3	13.5	26	32.1	7.9	0	4	3	2.71	1.2
77	2.8	18.1	14.9	26	26.5	11.7	0	4	3	2.63	1.2
78	7	11.6	11.6	23.3	20.9	25.6	0	4	3	2.53	1.3
79	3.3	10.2	16.7	32.1	25.1	12.5	0	4	3	2.75	1.1
80	1.4	7.9	11.6	32.1	41.9	5.1	0	4	3	3.11	1
81	31.6	17.7	10.2	7	3.7	29.8	0	4	1	1.05	1.2
82	28.8	21.9	13.5	9.8	4.7	21.4	0	4	1	1.23	1.2
83	13.5	21.9	25.1	23.7	11.2	4.7	0	4	2	1.97	1.2
84	8.8	23.3	28.4	24.7	10.7	4.2	0	4	2	2.05	1.1
85	11.6	26.5	17.7	26.5	10.7	7	0	4	2	1.98	1.2

86	7.4	26.5	25.6	23.7	9.8	7	0	4	2	2.02	1.1
87	0	0	4.2	11.2	17.2	67.4	2	4	4	3.4	0.7
88	0	0.9	2.8	10.7	18.1	67.4	1	4	4	3.41	0.8
89	2.3	2.8	7.9	8.8	10.7	67.4	0	4	3	2.7	1.2
90	0.5	1.9	7.4	9.3	13.5	67.4	0	4	3	3.03	1

Table 2 An overview of the ICF-based items with a non-gradable rate above 15%.

Rate (%)	H.	Question	Remarks
**14.9**	64	Do you have difficulty with doing your most important tasks well?	Not Applicable: 13.7%. As there was a note before this item saying, "Answer these items regarding the task you assigned to at school, university, paid or unpaid work", the respondents who were not occupied with these tasks chose "not applicable". To reduce this rate, a task can be defined in a more general matter including household tasks.
**16.3**	27	Do you have a problem with recognizing which instruments are playing when you are listening to music?	It is expected that differentiation of the musical instruments for people with no musical training "untrained ears" would be difficult.
**16.7**	63	Do you have difficulty with your day-to-day occupation/tasks?	Not Applicable: 15.5%. Refer to remark of H.64.
**21.4**	82	What is the extent to which darkness or insufficient light can be considered a barrier (e.g., in lip-reading)?	I don't know: 9.1%, Not applicable: 1.9%. Insufficient light might be mainly important only for unaided hearing-impaired persons or aided with a moderate to severe HL.
**25.6**	78	What is the extent to which you rate the general support received from the main health services and systems offered in relation to your hearing aids and medical services (e.g. ear specialist)?	Not applicable: 16%. The usage of the term hearing aids could potentially imply that the item is only needed for aided subjects. Besides, those that are not actively using medical services could find this item not applicable.
**28.8**	62	Do you have difficulty with performing communication techniques such as lip-reading?	I don't know: 16.4%, Not applicable:11.4%, see H.82.
**29.8**	81	What is the extent to which the design and construction of your workplace/task place can be considered a barrier? Think about video conferencing, as an example!	Not Applicable: 22.8%. Similar to H.63 and H.64, those who are not currently occupied chose not applicable.

Table 3 Sample scores for individuals.

Age, Gender, Country	PTA better / worse ears (dB HL)	Speech perception score	hearing-related score	non-hearing-related score	Health condition(s)	Remarks
**69, M, Germany**	54 / 56	1.47	1.41	1.08	Pain in neck / Paranasal and frontal sinus	He is classified as an individual with moderately severe hearing loss according to the PTA value. Based on the hearing-related and speech perception scores, he is classified in the mild group.
**71, M, Germany**	46 / 49	2.73	2.81	1.38	Hypertension and Circulatory disorders	He is classified as an individual with moderately severe hearing loss according to the PTA value. Based on the hearing-related and speech perception scores, he is classified in the moderate group. In this case, both classifications match.
**25, F, Egypt**	30 / 40	0.56	0.60	1.44	-	She is classified as an individual with mild hearing loss according to the PTA value. Based on the hearing-related and speech perception scores, he is classified in the normal group.
**51, M, USA**	12 / 12	4.76	5.16	4.38	Subjective hearing loss, pain in neck area, Hypertension, Tinnitus	He is categorized as having normal hearing based on the PTA value, despite having elevated scores in speech perception, as well as high scores in both hearing-related and non-hearing assessments. Here, a mismatch of the disability degree was observed.
**58, M, USA**	34 / 38	6.72	7.04	6.24	Hearing impaired, aided for one year, Hypertension	Even with the use of a hearing aid, he had high speech perception, hearing-related, and non-hearing-related scores.
